# Comparative efficacy of traditional and modern mind-body exercises in middle-aged and older adults with knee osteoarthritis: a network meta-analysis of randomized controlled trials

**DOI:** 10.3389/fmed.2026.1821838

**Published:** 2026-07-02

**Authors:** Rui Pan, Ao Mi, Hui Cheng

**Affiliations:** Physical Education College of Tianshui Normal University, Tianshui, China

**Keywords:** Baduanjin, knee osteoarthritis, mind-body exercises, network meta-analysis, Pilates, Tai Chi, Wuqinxi, Yijinjing

## Abstract

**Objective:**

Traditional and modern Mind-body exercise methods are widely used in the rehabilitation treatment of knee osteoarthritis (KOA), this network meta-analysis (NMA) compared the relative efficacy of various Mind-body exercise methods on KOA-related pain, function and stiffness, as well as psychological and physical outcomes.

**Methods:**

PubMed, EMBASE, Cochrane Library, and Web of Science were searched up to December 1, 2025. The main outcome measures included WOMAC pain score, WOMAC physical function score and WOMAC stiffness score. Secondary outcome indicators included mental health status, 6-min walking test (6MWT) and timed up-and-go test (TUG). RoB 2 was used to evaluate the risk of bias, and CINeMA was used to evaluate the certainty of network evidence. This research scheme has been registered in PROSPERO (CRD420261281876).

**Results:**

A total of 29 RCTs (*n* = 2,095) were included. The mean age of participants ranged from 58 to 79 years. Eight intervention measures were evaluated: Wuqinxi, Tai Chi, Pilates, Yijinjing, Baduanjin, yoga, conventional exercise, and no exercise intervention. Compared with no exercise intervention, Tai Chi and Wuqinxi significantly reduced WOMAC pain score. Pilates, Baduanjin, and Tai Chi significantly improved the WOMAC physical function score. Baduanjin significantly improved stiffness. Yijinjing, yoga, and Tai Chi significantly improved mental health.

**Conclusion:**

Different Mind-body exercises may provide outcome-specific benefits for KOA, particularly in middle-aged and older adults. Tai Chi and Wuqinxi appeared more favorable for pain relief, Pilates had the highest probability of improving physical function, Baduanjin appeared favorable for stiffness, and Yijinjing showed the highest ranking probability for mental health. Given the clinical diversity across trials and the low or very low certainty of several comparisons, SUCRA rankings should be interpreted cautiously. Specifically, TUG-related ranking results should be considered exploratory because the TUG network did not form a closed loop and inconsistency testing could not be performed; therefore, they should not be interpreted as definitive evidence. Further high-quality, long-term RCTs are needed to strengthen the evidence and support more precise exercise prescriptions.

**Systematic review registration:** https://www.crd.york.ac.uk/prospero/display_record.php?ID=CRD420261281876, identifier: CRD420261281876.

## 1 Introduction

Knee osteoarthritis (KOA) is a common chronic degenerative joint disorder and a leading cause of pain and functional limitation, particularly among middle-aged and older adults (1, 2). It is characterized by activity-related knee pain, short-lived stiffness, and progressive impairment in physical function, reflecting a whole-joint pathological process involving articular cartilage, subchondral bone, synovium, and periarticular structures (1, 2). Epidemiological evidence indicates that KOA represents one of the most prevalent forms of osteoarthritis worldwide, with a substantial and increasing disease burden as population aging accelerates (3, 4). Given its high prevalence and contribution to disability, KOA poses a major public health challenge with considerable individual and societal impacts.

The clinical treatment of KOA mainly aims at relieving pain and improving joint function. Drug therapy and intra-articular injection are commonly used interventions (5). However, although these treatments can effectively relieve symptoms in the short term, long-term use may lead to adverse reactions, such as gastrointestinal problems and multiple organ dysfunction (6). Therefore, it is very important to find a safer and more effective alternative therapy.

In recent years, exercise therapy as an auxiliary treatment for KOA has attracted more and more attention. Studies have shown that proper exercise can enhance the muscle strength around the knee joint, thus improving joint stability and delaying cartilage degeneration (7, 8). However, although exercise therapy can usually bring positive effects, its curative effect varies with different interventions. In particular, traditional Mind-body exercises have become a key component of KOA treatment. Different from traditional aerobic exercise or strength training, Mind-body exercises combined physical activity, breathing regulation, and meditation and may improve overall balance, flexibility and mental health, thus providing a more comprehensive rehabilitation effect (9–11). For example, Pilates can enhance the stability and strength of core muscles, improve pelvic and trunk stability, and then reduce the load of knee joint and enhance its function, which may relieve KOA symptoms (12). Yoga is characterized by its gentle and slow movements and breathing control, which is especially beneficial to middle-aged and older adults and has shown positive effects in KOA patients (13, 14). At the same time, the traditional Chinese medicine regimen (TCE) including Qigong, Tai Chi, and Baduanjin has accumulated hundreds of years of experience (15, 16). Guided by the concept of balance between yin and yang and overall health, TCE promotes the circulation of qi and blood, regulates the function of viscera, and stimulates the vitality of muscles and tendons by combining body movement, breathing control and meditation, thus serving as an adjuvant therapy (17, 18). Studies have shown that TCE can significantly relieve the pain, stiffness and dysfunction of patients with KOA (19–22).

Although both traditional and modern Mind-body exercises have proved to have positive effects on the rehabilitation of knee osteoarthritis, direct comparative research is still scarce. The purpose of this study is to systematically evaluate and compare the curative effects of various traditional and modern therapeutic effects on knee osteoarthritis through the network meta-analysis of RCT, with special attention to middle-aged and older adult patients, who are the main affected population in the rehabilitation of knee osteoarthritis. By comprehensively evaluating the relative effects of these interventions, this study aims to provide evidence-based guidance for clinicians, patients and decision makers.

## 2 Materials and methods

This NMA follows the Extended Guide for Network Meta-analysis (Supplementary Table 1) (23). In view of the fact that there is no RCT that directly compares different traditional and modern Mind-body exercise methods, this study adopts indirect comparison method to rank the curative effects of various exercise programs on patients with KOA in a probabilistic way (24). In order to ensure the transparency, reliability and originality of the research, this research scheme has been registered in the Prospective Registry of Systematic Review (CRD420261281876).

### 2.1 Data sources and search strategy

In this study, PubMed, EMBASE, Cochrane Library and Web of Science databases were searched comprehensively. The search terms include knee osteoarthritis, osteoarthritis, randomized clinical trial, psychosomatic therapy, psychosomatic medicine, Yijinjing, Qigong, Tai Chi, Baduanjin, Pilates, Wuqinxi, Tai Chi, and Yoga. We used a combination of controlled vocabulary and free-text terms (Supplementary Table 2). The search scope covers the establishment of databases until December 1, 2025. In order to ensure the comprehensiveness of retrieval, no language restrictions are set. In addition, the references of inclusion studies and related systematic reviews were consulted to identify other studies that may meet the inclusion criteria.

### 2.2 Selection criteria

#### 2.2.1 Inclusion criteria

1) Study Population: Studies included participants with a confirmed diagnosis of KOA. Consistent with the epidemiology of KOA, most study populations consisted of middle-aged and older adults, with a disease duration of at least 6 months. Individuals with inflammatory arthritis were excluded, as were those who had recently undergone knee surgery or intra-articular injections, as these could significantly influence the assessment of knee symptoms and function.

2) Study Design and Intervention: Only RCTs were included. The intervention group received traditional or modern Mind-body exercises, such as Yoga, Tai Chi, Wuqinxi, Baduanjin, or Pilates. The control group received either conventional exercise (e.g., stretching, flexibility exercises, or standard functional training) or no exercise intervention (e.g., health education, lectures, online follow-up, or supervision).

3) Outcome Measures: RCTs were required to report at least one of the following outcome measures.

The outcomes assessed in this study include: TUG (Timed Up and Go), which evaluates mobility and dynamic balance by measuring the time taken for participants to rise from a seated position, walk a designated distance, and return to the starting point; 6MWT (6-Min Walk Test), which assesses walking ability and aerobic endurance by recording the maximum distance walked in 6 min; the WOMAC Pain Subscale, which measures pain severity; the WOMAC Physical Function Subscale, which evaluates physical function; the WOMAC stiffness Subscale, which gauges joint stiffness; and mental health outcomes, which can be assessed using the SF-36 mental health dimensions (e.g., emotional wellbeing, social function, energy levels) or the SF-12 Mental Component Summary (MCS, on a 0–100 scale, where higher scores reflect better mental health).

#### 2.2.2 Exclusion criteria

1) Studies with duplicate reporting from the same trial population at different stages or follow-up points (for example, when multiple reports are derived from the same RCT, the version with the most comprehensive data, the most thorough outcome reporting, or the most recent publication will be prioritized, to prevent double-counting of participants).

2) Studies with unclear outcome definitions, unspecified scale sources, or those that fail to provide extractable data.

3) Non-randomized controlled trials, as well as reviews, case reports, or other non-original experimental studies.

Prior to the inclusion of RCTs, studies were initially screened based on their titles and abstracts. All included RCTs were subsequently verified by two reviewers to ensure that the data used was from the most recently published version.

### 2.3 Data extraction and quality assessment

The two researchers independently extracted data from RCTs and evaluated the quality of the literature according to the PRISMA guidelines for systematic review and meta-analysis. Any differences between the two reviewers are resolved through discussion and the participation of the third author. The extracted data include: the first author and the year of publication; The institute is in the country/region; Sample size (reported by group); Baseline demographic and clinical characteristics (such as average age, male ratio and BMI), it is worth noting that the average age of each study is usually between 58 and 79 years old, reflecting the characteristics of the middle-aged and older adult population; Follow-up duration (in weeks, units are converted at different times); And the detailed intervention information of the experimental group and the control group (including exercise type, frequency, duration per session, total duration and specific details of intervention). To further assess the assumptions of transitivity and comparability, we additionally extracted potential effect modifiers across studies, including baseline disease severity, baseline pain level, supervision intensity, exercise dose, and concurrent therapy or co-interventions. These baseline- and intervention-related characteristics were summarized in Supplementary Table 45 and were considered when evaluating indirectness in the CINeMA/GRADE assessment. For continuous outcome indicators, the average change value and standard deviation (SD) before and after intervention are given priority. If only the mean and standard deviation of the baseline and endpoint are reported, the average change value is calculated as the difference between these two time points. The standard deviation of variation score is calculated based on baseline standard deviation, end standard deviation and correlation coefficient (R) according to the following formula:


$$\begin{array}{l} SDchange=\\ \sqrt{Baseline\;SD^{2}+Endpoint\;SD^{2}-2R\cdot Baseline\;SD\cdot Endpoint\;SD} \end{array}$$

If the correlation coefficient cannot be obtained directly from the study, the correlation coefficient is obtained from the experiment with the largest sample size, the lowest risk of bias and the reported change value.

For studies without available change-score standard deviations, the primary correlation coefficient *R* = 0.845 was adopted to impute missing values, which was extracted from a published study with the largest sample size and lowest risk of bias (25). To test the robustness of the main findings to this statistical assumption, sensitivity analyses were further performed using two alternative plausible correlation coefficients of *R* = 0.5 and *R* = 0.25. The league table results of pairwise comparisons under *R* = 0.5 are presented in Supplementary Figures 13, 14, and those under *R* = 0.25 are shown in Supplementary Figures 15, 16.

In this study, ROB 2 tool was used for quality evaluation, which was based on the evidence-based principles of systematic evaluation and meta-analysis. The tool evaluates potential bias sources from five key areas: randomization process, deviation from expected interventions, missing outcome data, outcome measurement and selective reporting. According to each field and the overall biased risk judgment, the research is divided into three categories: low risk, some concerns or high risk.

### 2.4 Statistical analysis

In this study, Stata 17.0 MP was used for network meta-analysis. For continuous outcome variables, when the same scale and unit are used in each study, mean difference (MD) and 95% confidence interval (CI) are used. When the same outcome variable adopts different scales or units in different studies, the standardized mean difference (SMD) and 95% CI are used. For binomial outcome variables, the odds ratio (OR) and 95% CI were calculated. In multi-group experiments, pairwise comparisons are generated by using the enhanced format, so as to preserve the relevant structure within the study and prevent the bias of artificially lowering the standard error due to repeated use of the control group. The main analysis adopts the random-effects model, and assumes that the network is consistent, and uses the restricted maximum likelihood method (REML) to estimate the inter-study variance (τ). When the network has a closed-loop structure, the global inconsistency test is carried out to evaluate the overall consistency, while the local inconsistency is evaluated by node splitting method. Additionally, the *I*^2^statistic was calculated to quantitatively assess between-study heterogeneity. For all key pairwise comparisons across six primary and secondary outcomes, 95% prediction intervals (95% PIs) were generated to evaluate the variability and clinical applicability of pooled effects. Detailed results of τ^2^ and *I*^2^ are presented in Supplementary Table 46, and the plots of 95% prediction intervals are shown in Supplementary Figures 17–22. A value of *P* < 0.1 is considered to indicate possible inconsistency. The inconsistency factor (IF) is used to evaluate the cycle consistency. IF the 95% confidence interval of IF contains 0, there is no statistical evidence to support the inconsistency between direct evidence and indirect evidence. Generate a network diagram to visualize the geometric structure of the network. In the figure, the node size is directly proportional to the total sample size of each treatment, and the thickness of the connecting line represents the number of studies comparing the corresponding interventions. In order to rank the intervention measures, a variety of ranking indicators are adopted, including the area under cumulative ranking curve (SUCRA), the best treatment probability (PreBest) and the average ranking, which together improve the robustness and interpretability of the results.

Before interpreting indirect comparisons and SUCRA rankings, the transitivity assumption was assessed qualitatively by comparing the distribution of predefined potential effect modifiers across treatment comparisons. These factors included baseline disease severity, baseline pain level, follow-up duration, supervision intensity, exercise dose, comparator type, and concurrent therapy. The assumption was considered more plausible when these characteristics were broadly comparable across comparisons. When important imbalance or insufficient reporting was observed, this was considered in the assessment of indirectness and in the cautious interpretation of ranking probabilities. Evaluation of publication bias and small sample effect using comparison adjusted funnel chart. The sensitivity analysis is carried out by one-by-one elimination method, that is, each study is eliminated in turn, and the random effect consistency model is re-run to compare the direction and size of the merger effect. In addition, univariate network element regression analysis was carried out to explore the influence of covariates at the research level on the treatment effect, and the regression coefficient, 95% confidence interval and Wald test *P-value* were reported. *P* < 0.05 is considered to be statistically significant, indicating that there is a covariant modification effect.

### 2.5 GRADE grading

In this study, the GRADE framework is adopted, and the evidence certainty of the results of network meta-analysis is evaluated by combining Meta CINeMA. The preliminary evaluation results of RCT are classified as “high certainty.” The evaluation of evidence certainty covers six aspects: the risk of bias within the study, indirectness, inaccuracy, heterogeneity, inconsistency, and potential bias between studies (including publication bias and small sample effect). The risk of bias within the study was evaluated by RoB 2.0, and each aspect was investigated independently. These bias risks were then weighted according to their contribution to the network estimates and aggregated at the comparison level using CINeMA's contribution matrix. Indirectness was evaluated based on the assumptions of transitivity and exchangeability, with potential effect modifiers (such as baseline severity, intervention intensity, and follow-up duration) pre-specified. Consistency between direct and indirect evidence was assessed for population, intervention, comparator, and outcome measures. Imprecision was determined by assessing whether the 95% confidence intervals crossed the null value or the pre-set minimum clinically important difference (MID) threshold. Heterogeneity was primarily evaluated using the τ^2^ estimate from the random-effects model, taking into account the location of the prediction interval relative to the MID. In networks containing closed loops, inconsistency was assessed through CINeMA's built-in direct–indirect consistency evaluation. Bias between studies was examined by considering trial registration, searches for gray literature, and small sample effects, as suggested by comparison-adjusted funnel plots. Each domain was rated as “no concern,” “some concern,” or “serious concern,” and evidence was downgraded according to GRADE principles: one level for “some concern” and two levels for “serious concern.” Ultimately, the overall certainty of evidence was classified as high, moderate, low, or very low.

## 3 Results

### 3.1 Systematic review and characteristics of the included studies

**Figure 1:**
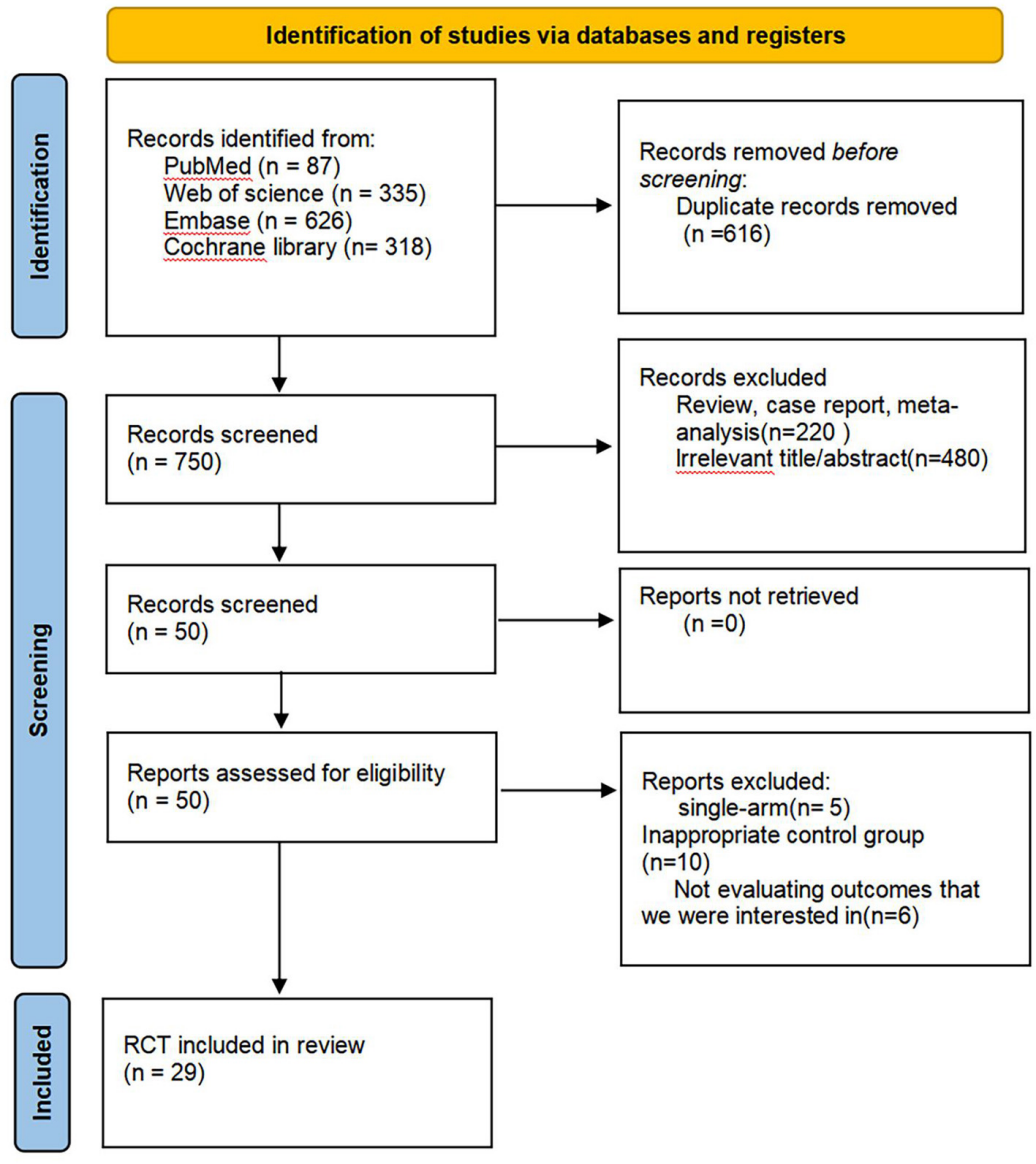
PRISMA 2020 flow diagram of study selection.

In the initial literature search, a total of 1,336 records were identified across the databases. Following abstract screening to exclude duplicates and irrelevant articles, 50 studies were deemed eligible for full-text review. Ultimately, 29 studies met the inclusion and exclusion criteria (Figure 1) (20–22, 25–50). This study included 2,095 patients who received one of the following eight interventions: Wuqinxi (WQX), Tai Chi (TC), Pilates (PIL), Yijinjing (YJJ), Baduanjin (BDJ), Yoga (YG), Conventional Exercise (CE), or No Exercise Intervention (NEI). A total of 29 RCTs were incorporated, spanning multiple data centers worldwide, including China (12 studies), the United States (10 studies), Australia (3 studies), and other countries. The overall sample represented primarily middle-aged and older adults, with mean ages ranging from 58 to 79 years, and a higher proportion of females. Follow-up durations varied across studies, typically ranging from 8 to 24 weeks. Overall, the included studies were broadly comparable in terms of KOA diagnosis, participant age, and follow-up duration. However, some clinical and intervention-related differences were observed. As shown in Supplementary Table 45, most studies enrolled symptomatic KOA participants according to ACR, NICE, or radiographic Kellgren–Lawrence criteria, and baseline pain levels were generally balanced between randomized groups. Nevertheless, the included trials differed in disease severity definitions, supervision intensity, exercise dose, comparator type, and concurrent therapy, including routine analgesics, NSAIDs, physical therapy, TENS, or usual care. These factors were therefore considered as potential effect modifiers when interpreting indirect comparisons and SUCRA rankings. Detailed information on the included studies is provided in Tables 1, 2, and Supplementary Table 45.

**Table 1:** General characteristics and baseline information of the included studies.

References	Age (Years)	Proportion of males (%)	Country	Follow-up duration (Weeks)	Sample size	BMI (kg/m^2^)
Tsai et al. (26)	79	27.27	United States	21	28/27	NR
An et al. (27)	65	0	China	8	11/10	25.7/25.4
Wang et al. (28)	60	29.9	United States	24	106/98	33.0/32.6
Nadia Saleem et al. (29)	58/56	0	Pakistan	8	20/20	25.8/26.9
Bennell et al. (30)	63/62	30.66	Australia	24	107/105	29.9/30.7
Rego et al. (31)	66/65	0	Brazil	7	8/9	28.9/32.0
Wortley et al. (32)	70/68	28	United States	10	13/12	30.5/35.1
Lee et al. (33)	60/61	25.58	United States	12	46/40	32.7/32.8
Zhu et al. (34)	65	0	China	24	23/23	25.2/25.1
Hu et al. (35)	66	0	China	24	52/40	36.5/26.4
Xiao et al. (21)	71/70	37.76	China	24	49/49	27.9/27.9
Kuntz et al. (36)	66/64	0	Canada	12	10/10	30.1/28.9
Xiao et al. (37)	71/70	33.82	China	12	34/34	27.9/27.9
Kang et al. (38)	63/65	0	China	36	12/15	25.6/24.3
Brismeé et al. (39)	71/69	17.07	United States	18	22/19	28.0/27.7
Evans et al. (40)	58/60	0	United States	8	11/15	NR
Cheung et al. (41)	69/72	0	United States	8	32/23	29.8/27.8
Ye et al. (42)	65/64	33.93	China	12	28/28	24.2/24.6
Ni et al. (43)	63	0	China	24	18/17	26.4/26.7
Wang et al. (44)	63/68	25	United States	24	20/20	30.0/29.8
Lee et al. (45)	70/67	6.82	South Korea	8	29/15	26.0/26.0
Song et al. (22)	64	0	China	12	20/20	24.6/24.4
Zhang et al. (46)	56/53	26	China	12	25/25	23.5/22.9
Ye et al. (47)	64/63	40	China	12	25/25	24.2/24.6
Nahayatbin et al. (48)	55/56	NR	Iran	4	16/16	29.0/29.0
Cheung et al. (49)	72	0	United States	8	18/18	29.1/28.8
Xiao and Li (20)	71/69	0	United States	24	142/142	29.8/28.4
Zhu et al. (25)	62/61	29.78	Australia	12	89/89	31.0/31.3
Abafita et al. (50)	61/64	27.35	Australia	24	58/59	29.4/28.4

XIAO, C.M. (a), XIAO, C.M. (b), YE, J.J. (a), and YE, J.J. (b) refer to two distinct studies, each employing different intervention approaches.

NR, no report.

**Table 2:** Intervention protocols and control conditions of the included randomized controlled trials (frequency, duration, and intervention details).

References	Intervention group prescription (Frequency/Duration per session/ Duration of intervention)	Control group prescription (Frequency/Duration per session/ Duration of intervention)	Intervention group protocol details	Control group protocol details
Tsai et al. (26)	3 times per week, for 20 weeks	3 times per week, for 20 weeks	**TC:**12-Form Sun-style Tai Chi	**NEI:** Health and Cultural Information Group
An et al. (27)	5 times per week, 30 min per session, for 8 weeks	No intervention	**BDJ:** Standardized Baduanjin exercise	**NEI:** No targeted treatments or interventions
Wang et al. (28)	2 times per week, 60 min per session, intensive intervention for 12 weeks, followed by encouragement for continued self-practice	For the first 6 weeks, 2 sessions per week (30 min per session in the clinic); for the following 6 weeks, 4 sessions per week (30 min per session at home); total intensive intervention duration: 12 weeks.	**TC:** Yang-style Tai Chi Protocol	**NEI:** Physical therapy
Nadia Saleem et al. (29)	2 times per week, 1 h per session (including preparatory physical therapy), for 8 weeks	2 times per week, 1 h per session (including preparatory physical therapy), for 8 weeks	**PIL:** Pilates	**CE:** Isometric training
Bennell et al. (30)	3 times per week, 30 min per session, online unsupervised practice, intensive intervention for 12 weeks	No mandatory frequency, self-access to educational website 3 times per week over 24 weeks, with no exercise-related interventions	**YG:** “My Joint Yoga” Unsupervised Online Yoga Program + Online Osteoarthritis Education	**NEI:** Online education
Rêgo et al. (31)	2 times per week, 60 min per session (including stretching, training, relaxation), intensive intervention for 7 weeks	No intervention, only 2 phone follow-ups per week, for 7 weeks	**PIL:** Mat Pilates	**CE:** No physical interventions or treatment guidance
Wortley et al. (32)	2 times per week, 60 min per session, intensive intervention for 10 weeks	No structured intervention, only 1 phone follow-up, for 10 weeks	**TC:** Yang-style Tai Chi	**NEI:** No targeted training interventions
Lee et al. (33)	2 times per week, 60 min per session, intensive intervention for 12 weeks	For the first 6 weeks, 2 sessions per week in the clinic (30 min each), for the following 6 weeks, 4 sessions per week at home (30 min each); total intervention duration: 12 weeks	**TC:** Standardized Modified Yang-style 10-Form Tai Chi	**NEI:** Physical therapy
Zhu et al. (34)	3 times per week, 60 min per session, intensive intervention for 24 weeks	1 offline lecture every 2 weeks (60 min) + 1 phone follow-up per week, for 24 weeks	**TC:** 24-Form Tai Chi adapted into an 8-Form Customized Program	**NEI:** Core intervention of health lectures and regular follow-ups, with no exercise-related interventions
Hu et al. (35)	3 times per week, 60 min per session, intensive intervention for 24 weeks	Regular participation in 60-min health lectures (frequency unspecified), no exercise intervention, for 24 weeks	**TC:** Structured Tai Chi Training Program	**NEI:** Core intervention of health lectures and self-maintenance of lifestyle, with no exercise-related interventions
Xiao et al. (2020a)	4 times per week, 60 min per session (including warm-up, core training, and relaxation), intensive intervention for 24 weeks	4 times per week, focusing on strength training and aerobic exercise, for 24 weeks	**WQX:** Modified Wuqinxi Protocol	**NEI:** Conventional physical therapy based on clinical guidelines
Kuntz et al. (36)	3 times per week, 60 min per session, intensive intervention for 12 weeks	3 times per week, 60 min per session of meditation and relaxation, with no exercise intervention, for 12 weeks	**YG:** Biomechanically Designed Yoga Protocol	**NEI:** Attention-equivalent control group, with no exercise interventions, participating only in meditation and relaxation courses
Xiao et al. (2020b)	4 times per week, 60 min per session (including warm-up, core training, and relaxation), intensive intervention for 12 weeks	4 times per week, focusing on home-based strength training and aerobic exercise, for 12 weeks	**WQX:** Home-based Wuqinxi Training Program	**NEI:** Home-based conventional physical therapy program
Kang et al. (38)	3 times per week, 60 min per session (including warm-up, core training, and relaxation), intensive intervention for 36 weeks	1 health lecture per month, 60 min, with no exercise intervention, for 36 weeks	**TC:** Tai Chi	**NEI:** Health lecture format, with no exercise-related interventions
Brismeé et al. (39)	0–6 weeks: 3 group sessions per week, 7–12 weeks: 3 home sessions per week, 13–18 weeks: no practice, total intervention duration 12 weeks	Only for the first 6 weeks, 3 health lectures per week, no intervention from week 7–18; total intervention duration: 6 weeks	**TC:** Standardized 24-Form Simplified Yang-style Tai Chi	**NEI:** Health lectures only, with no exercise-related interventions
Evans et al. (40)	2 times per week, 1.5 h per session, intensive intervention for 6 weeks	No additional interventions, only 1 follow-up monitoring session per week, for 6 weeks	**YG:** Iyengar Yoga Protocol	**NEI:** Maintenance of routine treatment
Cheung et al. (41)	1 offline session per week + 4 home practice sessions, for 8 weeks	1 phone follow-up per week, with no exercise intervention, for 8 weeks	**YG:** Hatha Yoga Protocol	**NEI:** Health education only, with no exercise-related interventions
Ye et al. (42)	3 sessions per week of Baduanjin training (40 min per session) + 5 sessions of routine therapy in the first 4 weeks, for 12 weeks	Only the first 4 weeks: 5 routine therapy sessions per week (1 h per session), no exercise intervention, for 12 weeks	**BDJ:** Baduanjin Qigong Training Combined with Mindfulness	**NEI:** Routine treatment, with no additional exercise interventions
Ni et al. (43)	24 weeks, divided into three phases, 2–4 times per week, 40 min per session, intensive intervention for 24 weeks	1 health lecture per week + stretching training, 45 min per session, for 24 weeks	**TC:**24-Form Simplified Yang-style Tai Chi Training	**CE:** Health lectures and stretching training, without Tai Chi-related exercise interventions
Wang et al. (44)	2 offline sessions per week + daily home practice, intensive intervention for 12 weeks, with follow-up until 48 weeks	2 offline health lectures per week + stretching training + daily home stretching, intensive intervention for 12 weeks, with follow-up until 48 weeks	**TC:** Modified Yang-style Tai Chi Training	**CE:** Health lectures and full-body stretching training
Lee et al. (45)	2 offline group sessions per week, 60 min per session, intensive intervention for 8 weeks	No intervention, only two assessments completed, for 8 weeks	**TC:** Simplified Tai Chi Qigong Training	**NEI:** No active interventions
Song et al. (22)	3 offline group sessions per week, 60 min per session, intensive intervention for 12 weeks	1 offline health lecture per week + exercise guidance, 60 min per session, for 12 weeks	**TC:** Modified 24-Form Yang-style Tai Chi	**NEI:** Primarily health education, with light home exercise guidance
Zhang et al. (46)	2 offline training sessions per week, 40 min per session, intensive intervention for 12 weeks	2 offline training sessions per week, 40 min per session, for 12 weeks	**YJJ:** Yijinjing Qigong Based on the Standard Set by the General Administration of Sport of China	**CE:** Targeted knee joint stretching and neuromuscular training
Ye et al. (47)	3 training sessions per week (40 min each), 4 weeks of in-hospital group practice followed by 8 weeks of home practice, total duration 12 weeks	No exercise intervention, only 3 assessments completed, for 12 weeks	**BDJ:** Baduanjin Qigong Training Based on the Standard Set by the General Administration of Sport of China	**NEI:** Maintain original lifestyle, with no additional exercise interventions
Nahayatbin et al. (48)	3 times per week, 20 min per session of Tai Chi training + 20 min of routine physical therapy, for 4 weeks	3 times per week, 20 min per session of routine physical therapy, for 4 weeks	**TC:** Simplified Yang-style Tai Chi 6th Form Training Combined with Conventional Physical Therapy	**NEI:** Only conventional physical therapy, with no additional exercise training
Cheung et al. (49)	1 offline group session per week (60 min) + 4 self-practice home sessions per week (30 min per session), for 8 weeks	No exercise intervention for the first 8 weeks, only data collection, followed by yoga intervention identical to the experimental group after 8 weeks	**YG:** Hatha Yoga	**NEI:** No yoga intervention for 8 weeks, maintaining original lifestyle and routine care
Xiao and Li (20)	6 times per week, 60 min per session (3 sets of training with rest between sets), for 24 weeks	No conventional exercise, only baseline, 12-week, and 24-week assessments, for 24 weeks	**WQX:** Wuqinxi Training Based on Traditional Chinese Medicine Theory	**NEI:** No regular physical exercise, maintaining original daily habits
Zhu et al. (25)	3 times per week, 45 min per session of online unsupervised Tai Chi practice, for 12 weeks	No exercise intervention, free access to educational website for 12 weeks, with 2 data collections	**TC:** Unsupervised Multimodal Online Yang-style Tai Chi Intervention, Combined with Educational Information and an Adherence Support App	**NEI:** Online osteoarthritis education resources only, with no Tai Chi-related content or app support
Abafita et al. (50)	First 12 weeks: 3 times per week (2 offline + 1 home), last 12 weeks: 3 home practice sessions per week, total duration 24 weeks, 1 h per session	Identical to the yoga group, for the first 12 weeks, 3 sessions per week (2 offline + 1 home), followed by 3 home practice sessions per week for the subsequent 12 weeks, total duration: 24 weeks, 1 h per session	**YG:** Professional Yoga Program Integrating Poses, Mindfulness, and Breathing Techniques	**CE:** Evidence-based lower limb strengthening training

### 3.2 Quality assessment of the literature

In this study, RoB 2 tool was used to evaluate the risk of bias in 29 RCTs. Among them, 15 studies were rated as low risk of bias, and 14 studies had some concerns. No study was rated as high risk of bias, indicating that the overall quality of the evidence included was relatively high. As for the randomization process, most studies clearly describe the generation methods of its random sequence (for example, computer-generated random sequence, random number table), and use sealed envelopes and other methods to implement allocation concealment, and report good baseline comparability. Therefore, the risk of bias in this field is generally low. However, a few studies only vaguely mention “random allocation” without specifying the hidden details of sequence generation or allocation, thus introducing potential selection bias, leading to the field being rated as “having some concerns.” In terms of the deviation between intervention measures and expected goals, most studies standardized exercise prescriptions and monitored compliance during the whole intervention process. In view of the challenge of blinding of participants and implementers in sports intervention, some studies have not clearly explained the blind method or compliance strategy in detail. This ambiguity may affect the subjective results, especially the results based on self-report, which leads to the field being rated as “there are some concerns.” For the missing result data, most studies report a low dropout rate and provide explanations for the missing data, which has little influence on the effect estimation. However, a few studies did not clearly explain how to deal with the missing data, or did not use intention-to-treat (ITT) analysis, which increased the sensitivity of the results to the missing data mechanism. These studies were also rated as “there are some concerns.” As for the measurement of results, most studies use standardized evaluation tools, such as WOMAC and NRS, and some studies also confirm the blind method of the evaluator, which helps to reduce the risk of measurement deviation. Nevertheless, a few studies did not sufficiently describe assessor training, measurement consistency, or blinding procedures, potentially introducing a slight risk of bias in measuring subjective outcomes. As such, concerns were raised in this domain. With respect to selective reporting, most studies provided comprehensive reports of predefined outcome measures, resulting in a low risk of selective reporting. However, a few studies reported incomplete secondary outcomes, inconsistent statistical analyses, or failed to align with predefined protocols, indicating potential risks of selective reporting. The domain-level and overall risk of bias assessments for each study are presented in Supplementary Figure 1.

### 3.3 Network meta-analyses

#### 3.3.1 Assessment of consistency and inconsistency

**Figure 2:**
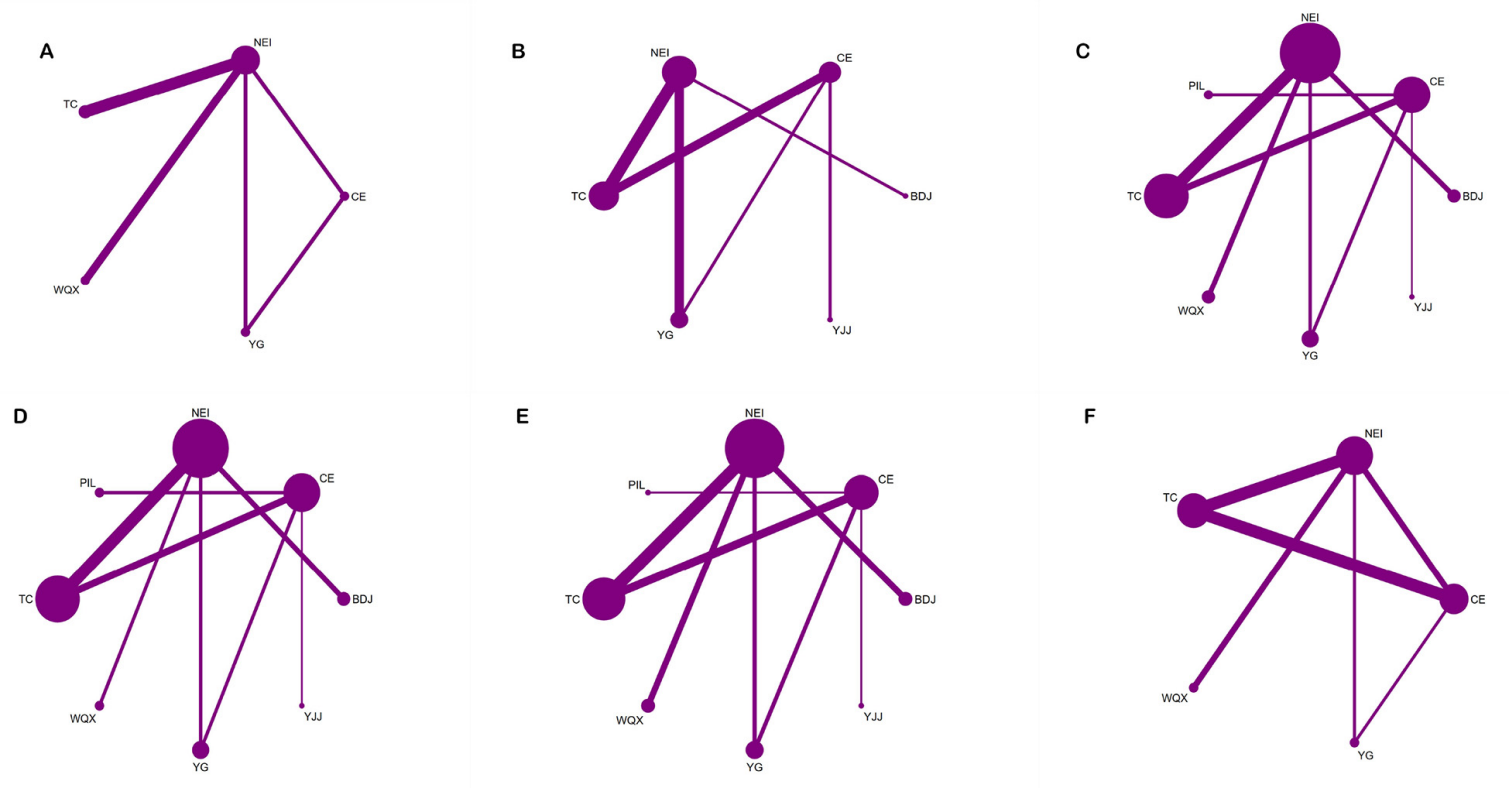
Network diagram comparing the efficacy of traditional and modern mind-body therapies in the rehabilitation of KOA. **(A)** TUG. **(B)** Mental Health. **(C)** WOMAC-Pain. **(D)** WOMAC-Physical Function. **(E)** WOMAC-Stiffness. **(F)** 6MWT.

In this study, WOMAC pain, WOMAC physical function, and WOMAC stiffness were considered the primary outcomes, while mental health, 6MWT, and TUG were secondary outcomes (Figure 2). A treatment comparison network was established for each outcome, followed by an assessment of consistency. For WOMAC pain, WOMAC physical function, WOMAC stiffness, mental health, and 6MWT, all networks formed closed loops, prompting the application of global inconsistency testing. The global inconsistency test showed *P-values* of 0.9047 for WOMAC pain, 0.6599 for WOMAC physical function, 0.6944 for WOMAC stiffness, 0.3957 for mental health, and 0.7909 for 6MWT, all of which were greater than 0.1, suggesting that the global consistency assumption was not rejected (see Supplementary Table 3). Further local inconsistency evaluations using the node-splitting method also yielded *P-values* greater than 0.1 for all comparison nodes (see Supplementary Tables 4–8). In addition, loop inconsistency analysis was conducted to assess the agreement between direct and indirect evidence, with the results revealing that the credibility intervals of the inconsistency factors crossed 0, indicating strong overall consistency within the network model (see Supplementary Figures 2–6). Consequently, the primary analysis was performed using a consistency model, based on the assumption of consistency.

In terms of between-study heterogeneity, the overall τ^2^and *I*^2^values across all outcomes are summarized in Supplementary Table 46, suggesting mild to moderate between-study variability. The 95% prediction intervals for all key comparisons (Supplementary Figures 17–22) further indicated that the pooled effect sizes were relatively stable across potential future similar studies, supporting the clinical applicability of our findings.

In addition to statistical inconsistency testing, clinical transitivity was assessed by comparing potential effect modifiers across the included comparisons. Supplementary Table 45 shows that most trials included middle-aged and older adults with symptomatic KOA and generally reported balanced baseline pain levels between intervention and control groups. However, differences were present in baseline disease severity, comparator type, supervision intensity, exercise dose, and concurrent therapy. These differences may partly modify treatment effects and were therefore considered when judging indirectness and interpreting ranking results. Thus, the absence of statistical inconsistency should not be interpreted as definitive proof of transitivity, but rather as supportive evidence when combined with clinical comparability.

Since the TUG outcome did not form a closed loop, global inconsistency testing, local inconsistency testing, and loop-specific inconsistency analysis could not be performed. Therefore, the interpretation of TUG relied mainly on the qualitative assessment of transitivity. Although age and follow-up duration were broadly similar across the included TUG studies, some uncertainty remained regarding intervention dose, supervision intensity, comparator type, and concurrent therapy. Accordingly, the certainty of evidence for TUG was downgraded in the indirectness domain, and the corresponding SUCRA ranking should be interpreted as exploratory rather than as definitive evidence of treatment superiority.

#### 3.3.2 TUG

**Figure 3:**
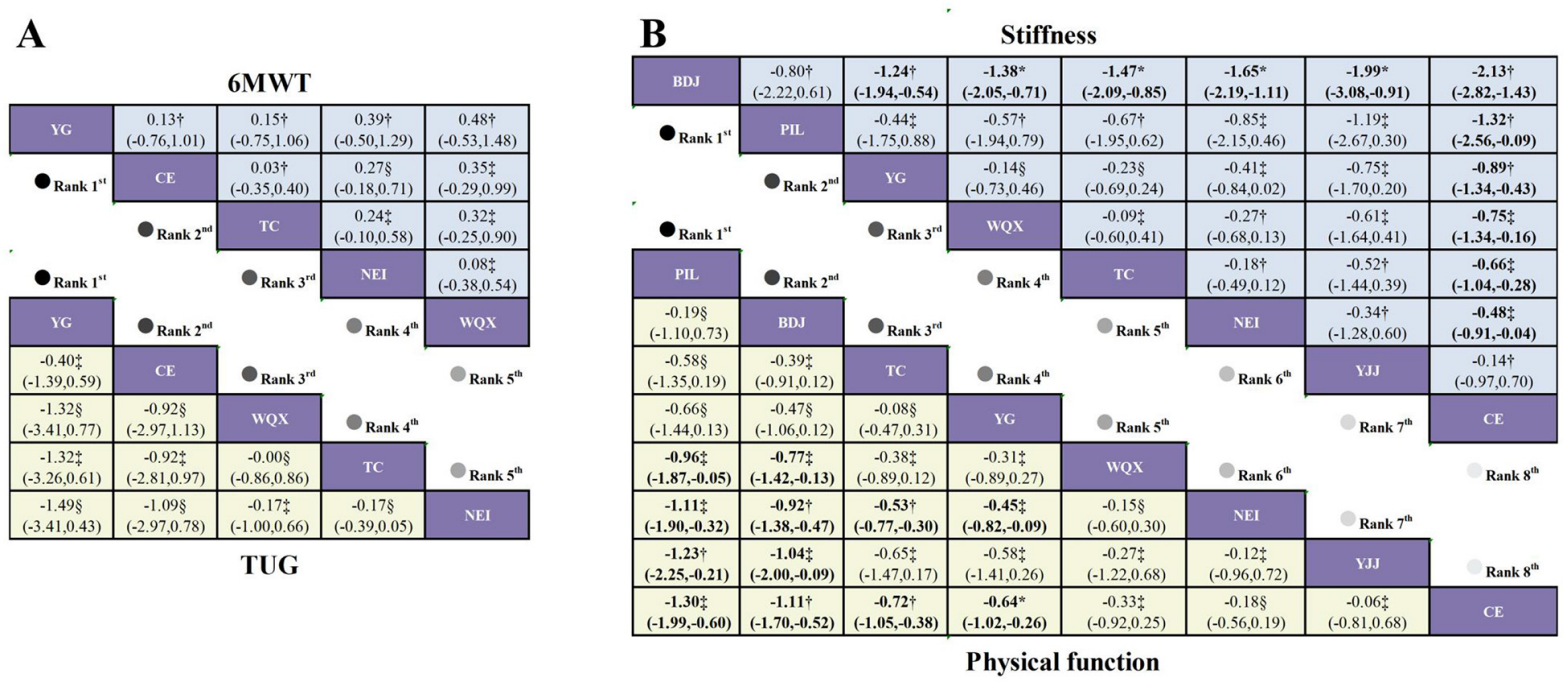
Network comparison of the rehabilitation effects of traditional and modern Mind-body therapies for KOA. **(A)** Shows the TUG test for assessing mobility and dynamic balance, and the 6MWT for evaluating walking ability and aerobic endurance. **(B)** Presents the WOMAC stiffness and the WOMAC physical function. Certainty of evidence: * = high, ^†^ = moderate, ^‡^ = low, ^§^ = very low.

**Figure 4:**
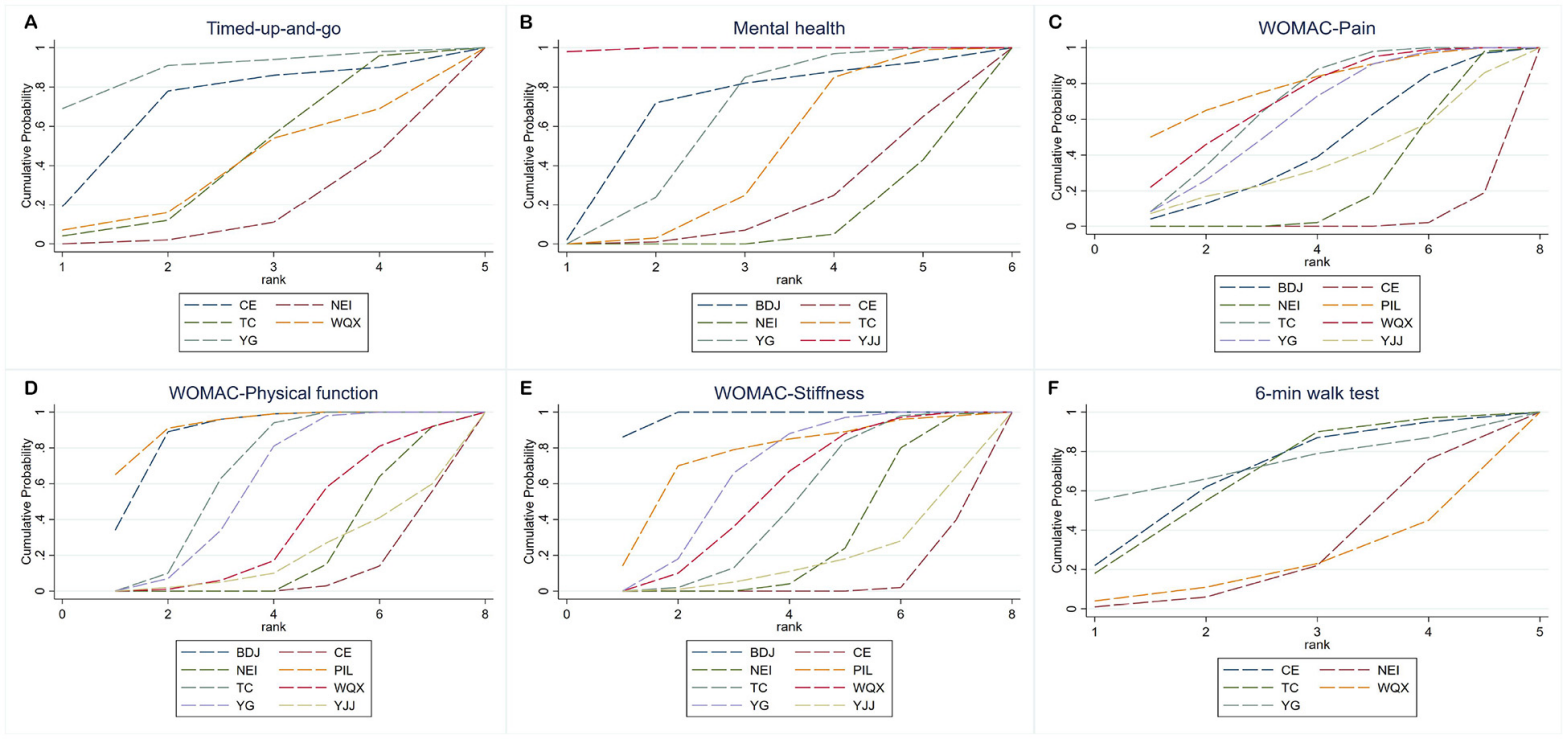
SUCRA diagram of different outcome measures. SUCRA, Surface Under the Cumulative Ranking Curve; **(A)** TUG. **(B)** Mental Health. **(C)** WOMAC-Pain. **(D)** WOMAC-Physical Function. **(E)** WOMAC-Stiffness. **(F)** 6MWT.

For the TUG test results, six studies involving 269 patients and five intervention schemes were included (see Figure 3A). All the results use the average difference (MD, in seconds) as the effect quantity, and the negative value indicates that the completion time of TUG is shortened, reflecting the improvement of function. Compared with NEI, all the estimated values of intervention effect did not reach statistical significance. However, YG intervention showed the most significant improvement trend (MD = −1.49, 95% CI: −3.41 to 0.43), followed by CE (MD = −1.09, 95% CI: −2.97 to 0.78) and WQX (MD = −0.17, 95% CI: −1.00 to 0.66). According to the ranking probability of SUCRA, YG (SUCRA = 87.9%) ranks the highest, followed by CE (68.4%) and WQX (36.5%), as shown in Figure 4 and Supplementary Tables 9–14. Because the TUG network did not form a closed loop and formal inconsistency testing could not be performed, these SUCRA-based rankings should be regarded as exploratory signals only and should not be interpreted as definitive evidence that one intervention is superior to another for TUG.

#### 3.3.3 6MWT

Ten studies (*n* = 621) were included in the results of 6-min walk test (6MWT), and five different intervention schemes were adopted in these studies (Figure 3A). Because there are differences in units of 6MWT, the SMD is calculated, and SMD > 0 indicates that walking endurance has improved. Compared with NEI, YG (SMD = 0.39, 95% CI: −0.50 to 1.29), CE (SMD = 0.27, 95% CI: −0.18 to 0.71), and TC (SMD = 0.24, 95% CI: −0.10 to 0.58). According to the ranking probability of SUCRA, YG ranks the highest (SUCRA = 71.7%), followed by CE (66.6%) and TC (64.9%) (Figure 4 and Supplementary Tables 9–14).

#### 3.3.4 WOMAC physical function

For the WOMAC physical function outcome, 23 studies (*n* = 1,579) were included, representing eight distinct intervention protocols (Figure 3B). Due to variations in scale versions and scoring ranges across the studies, SMD were used for synthesis, with SMD < 0 indicating improvement in WOMAC physical function. Compared to NEI, PIL (SMD = −1.11, 95% CI: −1.90 to −0.32), BDJ (SMD = −0.92, 95% CI: −1.38 to −0.47), TC (SMD = −0.53, 95% CI: −0.77 to −0.30), and YG (SMD = −0.45, 95% CI: −0.82 to −0.09) demonstrated statistically significant improvements. Based on the SUCRA ranking probabilities, PIL ranked highest (SUCRA = 92.8%), followed by BDJ (88.4%) and TC (66.7%) (Figure 4 and Supplementary Tables 9–14).

#### 3.3.5 WOMAC stiffness

For WOMAC stiffness score, 22 studies (*n* = 1,618) were included, representing 8 different intervention schemes (Figure 3B). Due to the differences in scale version and scoring range among the studies, SMD was used for comprehensive analysis, and SMD < 0 indicated that the symptoms of stiffness had improved. Compared with NEI, only BDJ group showed statistically significant improvement (SMD = −1.65, 95% CI: −2.19 to −1.11). The PIL group (SMD = −0.85, 95% CI: −2.15 to 0.46) and YG group (SMD = −0.41, 95% CI: −0.84 to 0.02) showed an improvement trend, but their 95% confidence interval included 0, indicating that it did not reach statistical significance. According to the ranking probability of SUCRA, BDJ ranks the highest (SUCRA = 98.0%), followed by PIL (75.8%) and YG (66.8%) (Figure 4) (see Supplementary Tables 9–14).

#### 3.3.6 Mental health

**Figure 5:**
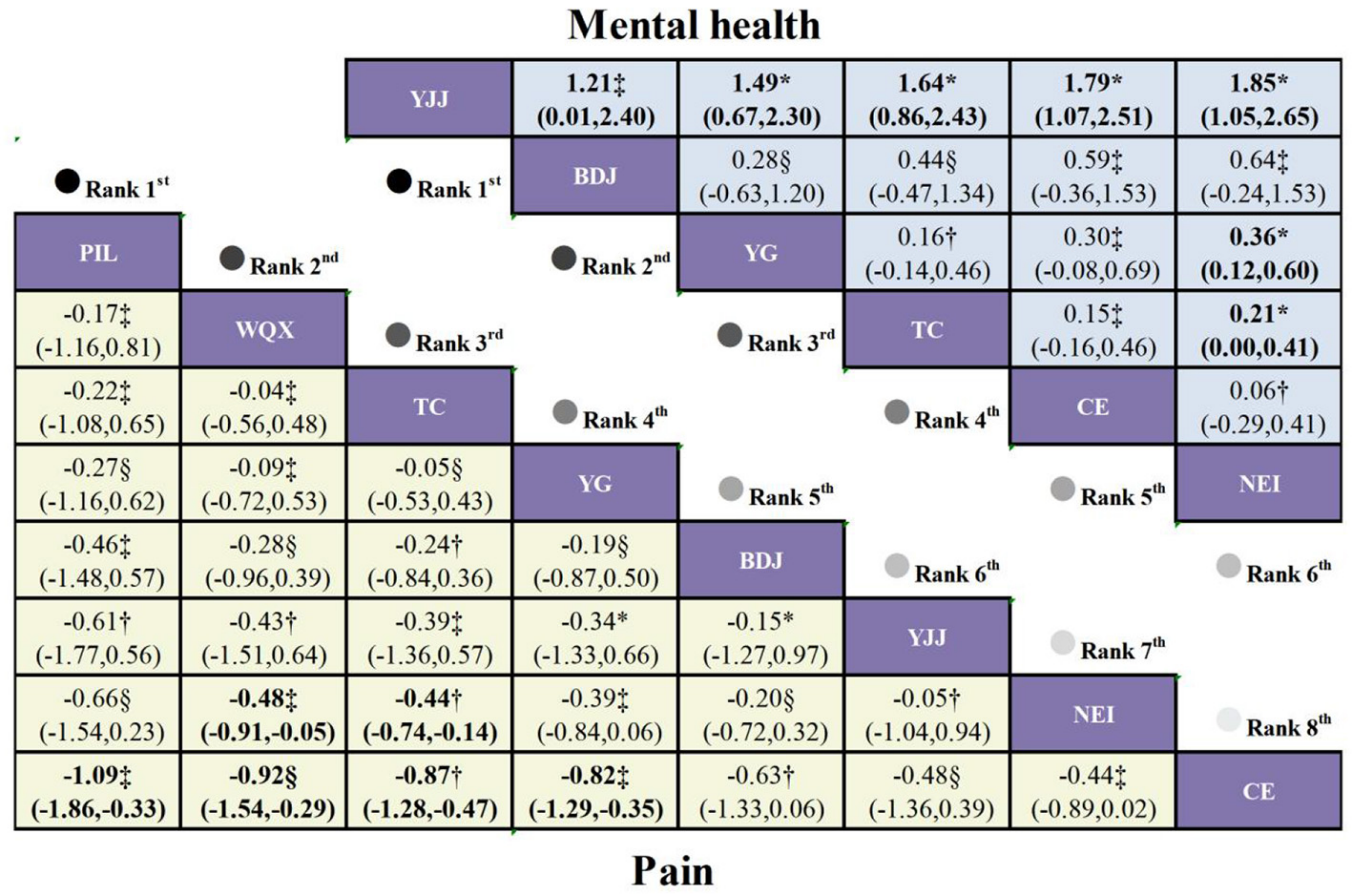
Network comparison of the rehabilitation effects of traditional and modern Mind-body therapies for KOA. Outcome measures include the WOMAC Pain and Mental Health for evaluating the effectiveness of interventions. Certainty of evidence: * = high, ^†^ = moderate, ^‡^ = low, ^§^ = very low.

In terms of mental health outcomes, 13 studies (*n* = 824) were included, covering six different intervention programs (Figure 5). Because the mental health dimension evaluation scales used in each study are different, including SF-36 and SF-12, the SMD is used for comprehensive analysis, and SMD >0 indicates that the mental health status has improved. Compared with NEI, YJJ (SMD = 1.85, 95% CI: 1.05 to 2.65), YG (SMD = 0.36, 95% CI: 0.12 to 0.60), and TC (SMD = 0.21, 95% CI: 0.00 to 0.41) showed improvements in mental health outcomes. According to the SUCRA ranking probabilities, YJJ ranked highest (SUCRA = 99.5%), followed by BDJ (67.5%) and YG (61.1%) (Figure 4 and Supplementary Tables 9–14).

#### 3.3.7 WOMAC pain

For the pain outcome of WOMAC, 24 studies involving 1,721 patients and 8 different intervention schemes were included (Figure 5). Due to the differences in scale version and scoring range among the studies, SMD was used for comprehensive analysis, and SMD < 0 indicated that WOMAC pain score was reduced. Compared with NEI, TC (SMD = −0.44, 95% CI: −0.74 to −0.14) and WQX (SMD = −0.48, 95% CI: −0.91 to −0.05) significantly improved the pain of patients with knee osteoarthritis. YG also showed an improvement trend (SMD = −0.39, 95% CI: −0.84 to 0.06), but the upper limit of its confidence interval crossed zero, indicating that the evidence was at a critical value and needed to be interpreted carefully. Although PIL also showed a tendency to relieve pain (SMD = −0.66, 95% CI: −1.54 to 0.23), the difference was not statistically significant. According to the SUCRA ranking probabilities, PIL ranked highest (SUCRA = 80.3%), followed by WQX (72.7%) and TC (70.3%), although the pairwise comparison between PIL and NEI did not reach statistical significance (Figure 4 and Supplementary Tables 9–14).

### 3.4 Sensitivity analysis, meta-regression, and publication bias

For all the outcome indicators, we conducted sensitivity analysis one by one to evaluate the influence of a single study on the estimated value of network effect. In each round of analysis, we eliminated one study and re-run the consistent random effect network meta-analysis with the remaining studies. The results show that no matter which study is excluded, the relative effect direction of each physical and mental intervention relative to NEI remains unchanged. The fluctuation of the effect quantity is very small, the 95% confidence intervals overlap significantly, and the statistical significance has not changed substantially, indicating the robustness of the main analysis results (Supplementary Tables 15–20).

In order to further explore the potential effect modifiers, we conducted univariate network meta-regression analysis on all the outcome indicators. The duration of intervention, the average age of participants and the study country/region were included in the analysis as covariables to evaluate their association with the effect of different interventions relative to NEI. Regression results show that these covariates have no statistically significant correlation with the estimated effect of any intervention relative to NEI (Supplementary Tables 21–38).

In addition, we draw a funnel chart for each outcome indicator to evaluate the small sample effect and potential publication bias. The scatter distribution of the funnel chart is generally symmetrical, and there is no obvious systematic bias or extreme abnormal value, indicating that the possibility of publication bias is low (Supplementary Figures 7–12).

The additional sensitivity analyses using alternative correlation coefficients for imputing change-score SDs (*R* = 0.5 and *R* = 0.25) did not materially alter the direction of treatment effects or the overall ranking pattern of interventions compared with the primary analysis, supporting the robustness of the findings (Supplementary Figures 13–16).

### 3.5 Network GRADE assessment

In this study, the CINeMA framework based on GRADE principle is used to evaluate the certainty of comparative evidence of each outcome indicator network. The quality of evidence varies from high to low according to the outcome index. For the TUG outcome indicators, a total of 10 comparisons were included, of which 4 (40%) were rated as low and 6 (60%) were rated as extremely low, indicating that the certainty of the evidence was generally low. For the mental health outcome, 15 comparisons were included, with 9 (60%) rated as high, 4 (26.67%) as moderate, and 2 (13.33%) as low, reflecting relatively high certainty in the evidence. In the WOMAC pain outcome, 28 comparisons were included, with 14 (50%) rated as moderate, 12 (42.86%) as low, and 2 (7.14%) as very low. For the WOMAC physical function outcome, 28 comparisons were included, with 1 (3.57%) rated as moderate, 15 (53.57%) as low, and 12 (42.86%) as very low, suggesting generally low certainty in the evidence. For the WOMAC stiffness outcome, 28 comparisons were included, with 4 (14.29%) rated as high, 12 (42.86%) as moderate, 10 (35.71%) as low, and 2 (7.14%) as very low. For the 6MWT outcome, 10 comparisons were included, with 5 (50%) rated as moderate, 4 (40%) as low, and 1 (10%) as very low. The network GRADE assessments for each outcome are provided in Supplementary Tables 39–44. Because several comparisons, particularly for TUG and WOMAC physical function, were supported by low or very low certainty evidence, the SUCRA rankings for these outcomes were interpreted cautiously and were not considered definitive evidence of treatment superiority.

## 4 Discussion

This study conducted a network meta-analysis to combine data from 29 RCTs involving primarily middle-aged and older adults with KOA, systematically comparing and ranking the relative efficacy of various traditional and modern Mind-body interventions for core outcomes in KOA. The results show that different types of exercise have different benefits across outcomes. Pilates showed the highest probability of improving body function, while Tai Chi and Wuqinxi appeared more favorable in relieving pain. Baduanjin appeared to be the most favorable for alleviating stiffness, while Yijinjing and yoga had a higher probability of improving mental health. However, in the TUG and 6-min walking tests, no Mind-body intervention consistently showed significant advantages. These ranking results should be interpreted in light of the transitivity assumption and the certainty of evidence. Although Supplementary Table 45 suggests that the included studies were broadly comparable in terms of KOA diagnosis, age distribution, and baseline pain balance, differences remained in disease severity, comparator type, supervision intensity, exercise dose, and concurrent therapy. These factors may act as potential effect modifiers and may influence indirect comparisons. Therefore, SUCRA values should be regarded as probability-based summaries within the current evidence network rather than definitive evidence of clinical superiority. This is particularly important when a treatment ranks highly but does not show a statistically significant pairwise effect, such as Pilates for WOMAC pain. Similarly, rankings for outcomes with low or very low certainty evidence should be interpreted cautiously and should not replace individualized clinical judgment.

As far as physical function is concerned, Pilates ranks the highest because of its emphasis on core stability, hip and knee movement chain control and posture correction. These factors can effectively improve the common compensatory exercise patterns and abnormal joint load distribution in patients with knee osteoarthritis, thus significantly improving the WOMAC physical function subscale and other indicators, which focuses on the limited degree of daily activities (51). In contrast, traditional Mind-body exercises such as Tai Chi and Baduanjin can also improve motor control, balance and proprioception. However, their training effects are more diverse and depend on training frequency, action accuracy and compliance. This difference is consistent with the results of our sensitivity analysis. Although the confidence intervals of some comparisons are wide, the overall results are still consistent.

For the symptoms of joint stiffness, Ba Duan Jin showed remarkable curative effect, indicating that low impact, whole body multi-joint exercise combined with rhythmic stretching may be particularly effective in relieving the clinical manifestations of joint stiffness (52). Baduanjin's repeatability, adjustable range of motion and relatively low learning curve make it very suitable for continuous training and gradual load. At the local joint level, Baduanjin can enhance joint mobility, improve the compliance of tendons and joint capsules, and promote local blood circulation and synovial fluid exchange-these effects may directly affect the subjective feeling of joint stiffness (53). These findings are consistent with the principle of non-surgical treatment of knee osteoarthritis, which takes conventional exercise as the core intervention measure (5).

The pain of KOA is not only the result of structural injury; Factors such as pain sensitization, negative emotions, sleep disorders and exercise fear will also affect the degree of pain (2). Tai Chi and Wuqinxi combine low-intensity aerobic exercise, balance and coordination training with concentration. These exercises help to reduce joint impact and improve joint stability and gait control. In addition, through rhythmic breathing and meditation, they can enhance the central processing of pain and stress response, thus achieving a more stable improvement in the pain assessment scale (54). These characteristics make this kind of Mind-body exercises especially suitable for middle-aged and older adults, who usually need low-impact and balance-oriented rehabilitation methods.

The observed benefits of Yijinjing and yoga on mental health indicate that physical and mental intervention may be an underestimated aspect in the comprehensive treatment of osteoarthritis. This association may be more significant among middle-aged and older adults, because they are faced with age-related decline in mobility, chronic pain, depression, anxiety and decline in quality of life. Pain and functional limitations heighten the psychological burden, while psychological distress exacerbates pain perception, lowers exercise adherence, and reduces self-efficacy (55). Both Yijinjing and Yoga incorporate stable breathing techniques, mindfulness, and body awareness, which offer a more substantial psychological training component (56). Moreover, the network GRADE analysis in this study indicated a higher level of certainty in the evidence for mental health outcomes, with improved measurement consistency and relatively low heterogeneity across the included trials.

The absence of significant differences observed in the TUG and 6MWT outcomes does not imply that these interventions are ineffective. Several factors may account for this. Most interventions in the included studies were conducted over a period of 8 to 24 weeks. If sufficient thresholds for muscle strength and cardiorespiratory fitness were not achieved, TUG performance could have been influenced by baseline fall risk, pain fluctuations, learning effects, and the level of test standardization. Additionally, under conditions of small sample sizes, the effects may have been more variable. Therefore, future research should implement standardized testing procedures to obtain objective functional results, determine the smallest clinically important differences, and consider stratification (for example, baseline walking ability and pain sensitivity level) to improve comparability and clinical interpretability.

Our findings are generally consistent with previous meta-analyses demonstrating the benefits of Mind-body exercises for KOA, but this study provides several important incremental contributions that address key gaps in the recent 2024–2025 literature.

First, this study provides the most comprehensive coverage of Mind-body exercise modalities to date. Previous network meta-analyses have been limited in their scope of interventions. For example, Tao et al. (57) only evaluated traditional Chinese Mind-body exercises (Tai Chi, Baduanjin, Wuqinxi) and excluded modern modalities such as Pilates and Yoga. Similarly, Gao et al. (11) included only 5 intervention types and did not assess Yijinjing. In contrast, our study is the first to systematically compare 8 distinct Mind-body interventions (5 traditional Chinese exercises + 3 modern exercises) in a single network, providing the most complete head-to-head comparison of available exercise options for KOA rehabilitation. This comprehensive coverage allows clinicians to make more informed choices between different exercise types based on specific patient needs.

Furthermore, our analysis is based on the largest and most up-to-date evidence base currently available. We included 29 RCTs and 2095 participants, representing the largest sample size of any network meta-analysis on this topic published to date. We also incorporated the latest evidence from 2025, including two high-quality RCTs published in JAMA Internal Medicine and JAMA Network Open (25, 50), which were not included in earlier reviews (11, 57, 58). This larger and more recent dataset increases the statistical power of our estimates and reduces the risk of bias from small-study effects.

In addition, we evaluated a broader range of clinically relevant outcomes than most previous studies. Most previous reviews have focused almost exclusively on the core WOMAC outcomes (pain, function, stiffness) and have neglected important functional and psychological outcomes. For example, Qiao et al. (58) did not assess objective physical function measures such as the 6MWT or TUG test, and no previous network meta-analysis has comprehensively evaluated the effects of different Mind-body exercises on mental health outcomes in KOA patients. Our study addresses this gap by including 6 clinically important outcomes, demonstrating that different exercises have distinct benefits across physical and psychological domains.

Importantly, we conducted a rigorous evidence certainty assessment using the CINeMA framework. While many previous network meta-analyses have provided SUCRA rankings, few have systematically evaluated the certainty of the underlying evidence. Our study is the first to apply the CINeMA framework specifically to Mind-body exercises for KOA, providing transparent and standardized ratings of evidence certainty for all pairwise comparisons. This allows readers to appropriately interpret the strength of our findings and understand the limitations of the current evidence base.

Notably, our study has a targeted focus on middle-aged and older adults, who are the primary population affected by KOA. Many previous reviews have included studies with younger populations or have not explicitly stratified their results by age. In contrast, all included studies in our analysis had a mean age of 55 years or older, ensuring that our findings are directly applicable to the population most in need of KOA rehabilitation interventions.

This study emphasizes that the effects of various Mind-body exercises on pain, stiffness, function and mental health are not completely consistent. Future mechanism research should focus on three key areas: biomechanics (joint load and exercise chain), neuroimmunology (pain sensitization and inflammation regulation), and psychological and behavioral factors (self-efficacy and fear avoidance). Based on the sorting evidence of this study, we can formulate a symptom-oriented exercise prescription strategy for communities and primary medical institutions. For individuals who are mainly troubled by dysfunction, Pilates could be considered as a favorable option based on ranking probability; for stiffness, Baduanjin tended to be a more favorable choice; for pain, Tai Chi or Wuqinxi had higher ranking probability for pain relief; for individuals with emotional or quality of life challenges, Yijinjing or yoga are likely to bring more benefits to mental health. This method is consistent with the current guidelines, which emphasize “exercise and self-management as the cornerstone, with a focus on patient preferences and accessibility,” provide low-cost and sustainable choices, have significant health and economic benefits, and help improve long-term compliance (5).

There are some limitations worthy of consideration in this study. First of all, due to the nature of sports intervention, it is not feasible to implement blind method for participants and intervention providers. In addition, subjective indicators such as pain, function and mental health may be affected by the expected effect. However, through consistency and inconsistency evaluation, sensitivity analysis and publication bias evaluation, the robustness of this study has been enhanced. Second, although we summarized key baseline and intervention-related characteristics in Supplementary Table 45 to support the assessment of transitivity, residual clinical heterogeneity remained across studies. Differences in baseline disease severity, baseline pain level, supervision intensity, exercise dose, comparator type, and concurrent therapy may have acted as potential effect modifiers. In particular, trials using active comparators such as conventional exercise or physical therapy may not be fully comparable with trials using wait-list, education, or no-exercise controls. Similarly, fully supervised interventions may differ from home-based or online unsupervised programs in adherence and non-specific therapeutic effects. Therefore, indirect comparisons and SUCRA rankings should be interpreted as comparative signals rather than definitive treatment hierarchies. Notably, the 95% prediction intervals for main outcomes (Supplementary Figures 17–22) also verified the generalizability of our pooled results in clinical practice. Third, the number of studies in some nodes is relatively small, the follow-up time is short, and the Asian population is dominant, which limits the universality of the research results in long-term effects, recurrence and cross-cultural applicability. In order to improve the universality and external validity of these conclusions, multi-center and multi-group randomized trials should be included in future research, and the follow-up time should be extended.

## 5 Conclusion

This network meta-analysis suggests that different traditional and modern Mind-body exercises may have outcome-specific benefits for KOA. Tai Chi and Wuqinxi appeared more favorable for pain relief, Pilates showed the highest probability of improving physical function, Baduanjin appeared the most favorable for stiffness, and Yijinjing showed the highest ranking probability for mental health. However, these rankings should not be interpreted as definitive treatment hierarchies because several comparisons were supported by low or very low certainty evidence and relied partly on indirect comparisons. Moreover, the TUG findings require particular caution: because no closed loop was formed for the TUG network, consistency testing was not feasible, and TUG-related SUCRA rankings should be regarded as exploratory rather than definitive evidence of treatment superiority. Clinical selection should therefore consider patient goals, baseline disease severity, pain level, exercise tolerance, supervision feasibility, concurrent therapy, and available resources. Further adequately powered, multicentre RCTs with standardized intervention reporting and longer follow-up are needed to confirm these findings.

## Data Availability

The original contributions presented in the study are included in the article/Supplementary material, further inquiries can be directed to the corresponding author.
