# Supplementary material for: Comparative efficacy of traditional and modern mind-body exercises in middle-aged and older adults with knee osteoarthritis: a network meta-analysis of randomized controlled trials
Source: Front Med (Lausanne). 2026 Jul 2;13:1821838. doi: 10.3389/fmed.2026.1821838 (PMC13372910; doi:10.3389/fmed.2026.1821838)
Supplement: Supplementary file 1 [file Table_1.docx]

**Table S1** PRISMA NMA Checklist of Items to Include When Reporting a Systematic Review Involving a Network Meta-analysis

| Section/Topic | Item # | Checklist Item | Reported on Page # |
| --- | --- | --- | --- |
| TITLE |  |  |  |
| Title | 1 | Identify the report as a systematic review *incorporating*  anetwork meta-analysis (or related form of meta-analysis). | 1 |
|  |  |  |  |
| ABSTRACT |  |  |  |
| Structured summary | 2 | Provide a structured summary including, as applicable:  **Background:** main objectives  **Methods:** data sources; study eligibility criteria, participants, and interventions; study appraisal; and *synthesis methods, such as network meta-analysis.*  **Results:** number of studies and participants identified; summary estimates with corresponding confidence/credible intervals; treatment rankings may also be discussed. Authors may choose to summarize pairwise comparisons against a chosen treatment included in their analyses for brevity.  **Discussion/Conclusions:** limitations; conclusions and implications of findings.  **Other:** systematic review registration number with registry name. | 1 |
|  |  |  |  |
| INTRODUCTION |  |  |  |
| Rationale | 3 | Describe the rationale for the review in the context of what is already known*, including mention of why a network meta-analysis has been conducted.* | 1-2 |
| Objectives | 4 | Provide an explicit statement of questions being addressed, with reference to participants, interventions, comparisons, outcomes, and study design (PICOS). | 2 |
|  |  |  |  |
| METHODS |  |  |  |
| Protocol and registration | 5 | Indicate whether a review protocol exists and if and where it can be accessed (e.g., Web address); and, if available, provide registration information, including registration number. | 2-3 |
| Eligibility criteria | 6 | Specify study characteristics (e.g., PICOS, length of follow-up) and report characteristics (e.g., years considered, language, publication status) used as criteria for eligibility, giving rationale. *Clearly describe eligible treatments included in the treatment network, and note whether any have been clustered or merged into the same node (with justification).* | 3 |
| Information sources | 7 | Describe all information sources (e.g., databases with dates of coverage, contact with study authors to identify additional studies) in the search and date last searched. | 3 |
| Search | 8 | Present full electronic search strategy for at least one database, including any limits used, such that it could be repeated. | 3，Supplementary  TableS2 |
| Study selection | 9 | State the process for selecting studies (i.e., screening, eligibility, included in systematic review, and, if applicable, included in the meta-analysis). | 3-4，Table 1 |
| Data collection process | 10 | Describe method of data extraction from reports (e.g., piloted forms, independently, in duplicate) and any processes for obtaining and confirming data from investigators. | 4 |
| Data items | 11 | List and define all variables for which data were sought (e.g., PICOS, funding sources) and any assumptions and simplifications made. | 3-4 |
| Geometry of the network | S1 | Describe methods used to explore the geometry of the treatment network under study and potential biases related to it. This should include how the evidence base has been graphically summarized for presentation, and what characteristics were compiled and used to describe the evidence base to readers. | 4 |
| Risk of bias within individual studies | 12 | Describe methods used for assessing risk of bias of individual studies (including specification of whether this was done at the study or outcome level), and how this information is to be used in any data synthesis. | 5 |
| Summary measures | 13 | State the principal summary measures (e.g., risk ratio, difference in means). Also describe the use of additional summary measures assessed, such as treatment rankings and surface under the cumulative ranking curve (SUCRA) values, as well as modified approaches used to present summary findings from meta-analyses. | 5 |
| Planned methods of analysis | 14 | Describe the methods of handling data and combining results of studies for each network meta-analysis. This should include, but not be limited to:   - Handling of multi-arm trials; - Selection of variance structure; - Selection of prior distributions in Bayesian analyses; and - Assessment of model fit. | 5 |
| Assessment of Inconsistency | S2 | Describe the statistical methods used to evaluate the agreement of direct and indirect evidence in the treatment network(s) studied. Describe efforts taken to address its presence when found. | 5 |
| Risk of bias across studies | 15 | Specify any assessment of risk of bias that may affect the cumulative evidence (e.g., publication bias, selective reporting within studies). | 5 |
| Additional analyses | 16 | Describe methods of additional analyses if done, indicating which were pre-specified. This may include, but not be limited to, the following:   - Sensitivity or subgroup analyses; - Meta-regression analyses; - Alternative formulations of the treatment network; and - Use of alternative prior distributions for Bayesian analyses (if applicable). | 5 |
| RESULTS† |  |  |  |
| Study selection | 17 | Give numbers of studies screened, assessed for eligibility, and included in the review, with reasons for exclusions at each stage, ideally with a flow diagram. | 5-6，Fig1 |
| Presentation of network structure | S3 | Provide a network graph of the included studies to enable visualization of the geometry of the treatment network. | 13-14，Fig2 |
| Summary of network geometry | S4 | Provide a brief overview of characteristics of the treatment network. This may include commentary on the abundance of trials and randomized patients for the different interventions and pairwise comparisons in the network, gaps of evidence in the treatment network, and potential biases reflected by the network structure. | 14-15 |
| Study characteristics | 18 | For each study, present characteristics for which data were extracted (e.g., study size, PICOS, follow-up period) and provide the citations. | 7-12,Table 1 |
| Risk of bias within studies | 19 | Present data on risk of bias of each study and, if available, any outcome level assessment. | 13,Figure 2 |
| Results of individual studies | 20 | For all outcomes considered (benefits or harms), present, for each study: 1) simple summary data for each intervention group, and 2) effect estimates and confidence intervals. *Modified approaches may be needed to deal with information from larger networks.* | 14-15 |
| Synthesis of results | 21 | Present results of each meta-analysis done, including confidence/credible intervals. In larger networks, authors may focus on comparisons versus a particular comparator (e.g. placebo or standard care), with full findings presented in an appendix. League tables and forest plots may be considered to summarize pairwise comparisons. If additional summary measures were explored (such as treatment rankings), these should also be presented. | 14-15 |
| Exploration for inconsistency | S5 | Describe results from investigations of inconsistency. This may include such information as measures of model fit to compare consistency and inconsistency models, *P* values from statistical tests, or summary of inconsistency estimates from different parts of the treatment network. | 13-14 |
| Risk of bias across studies | 22 | Present results of any assessment of risk of bias across studies for the evidence base being studied. | 15-16,Supplementary Figure S7-12 |
| Results of additional analyses | 23 | Give results of additional analyses, if done (e.g., sensitivity or subgroup analyses, meta-regression analyses*, alternative network geometries studied, alternative choice of prior distributions for Bayesian analyses,* and so forth). | 16 |
|  |  |  |  |
| DISCUSSION |  |  |  |
| Summary of evidence | 24 | Summarize the main findings, including the strength of evidence for each main outcome; consider their relevance to key groups (e.g., healthcare providers, users, and policy-makers). | 16-17 |
| Limitations | 25 | Discuss limitations at study and outcome level (e.g., risk of bias), and at review level (e.g., incomplete retrieval of identified research, reporting bias). *Comment on the validity of the assumptions, such as transitivity and consistency. Comment on any concerns regarding network geometry (e.g., avoidance of certain comparisons).* | 17-18 |
| Conclusions | 26 | Provide a general interpretation of the results in the context of other evidence, and implications for future research. | 18 |
|  |  |  |  |
| FUNDING |  |  |  |
| Funding | 27 | Describe sources of funding for the systematic review and other support (e.g., supply of data); role of funders for the systematic review. This should also include information regarding whether funding has been received from manufacturers of treatments in the network and/or whether some of the authors are content experts with professional conflicts of interest that could affect use of treatments in the network. | 18 |

PICOS = population, intervention, comparators, outcomes, study design.

* Text in italics indicate S wording specific to reporting of network meta-analyses that has been added to guidance from the PRISMA statement.

† Authors may wish to plan for use of appendices to present all relevant information in full detail for items in this section

| **Table S2.Literature Search Strategy** | | |
| --- | --- | --- |
| **PubMed**(December 1, 2025) | | |
| #1 | "osteoarthritis, knee"[MeSH Terms] | 33198 |
| #2 | "osteoarthritis, knee"[MeSH Terms] OR "knee osteoarthritides"[Title/Abstract] OR "knee osteoarthritis"[Title/Abstract] OR "osteoarthritis of knee"[Title/Abstract] OR "osteoarthritis of the knee"[Title/Abstract] | 42179 |
| #3 | "Mind-Body Therapies"[MeSH Terms] | 52023 |
| #4 | "Mind-Body Therapies"[MeSH Terms] OR "Mind-Body Therapies"[Title/Abstract] OR "mind body therapy"[Title/Abstract] OR "therapies mind body"[Title/Abstract] OR "therapy mind body"[Title/Abstract] OR "mind body medicine"[Title/Abstract] OR "mind body medicine"[Title/Abstract] OR "Yijinjing"[Title/Abstract] OR "Qigong"[Title/Abstract] OR "Taichi"[Title/Abstract] OR "Baduanjin"[Title/Abstract] OR "Pilates"[Title/Abstract] OR "Wuqinxi"[Title/Abstract] OR "tai ji quan"[Title/Abstract] OR "Yoga"[Title/Abstract] | 58902 |
| #5 | #2 AND #4 | 279 |
| #6 | (("osteoarthritis, knee"[MeSH Terms] OR "knee osteoarthritides"[Title/Abstract] OR "knee osteoarthritis"[Title/Abstract] OR "osteoarthritis of knee"[Title/Abstract] OR "osteoarthritis of the knee"[Title/Abstract]) AND ("Mind-Body Therapies"[MeSH Terms] OR "Mind-Body Therapies"[Title/Abstract] OR "mind body therapy"[Title/Abstract] OR "therapies mind body"[Title/Abstract] OR "therapy mind body"[Title/Abstract] OR "mind body medicine"[Title/Abstract] OR "mind body medicine"[Title/Abstract] OR "Yijinjing"[Title/Abstract] OR "Qigong"[Title/Abstract] OR "Taichi"[Title/Abstract] OR "Baduanjin"[Title/Abstract] OR "Pilates"[Title/Abstract] OR "Wuqinxi"[Title/Abstract] OR "tai ji quan"[Title/Abstract] OR "Yoga"[Title/Abstract])) AND (randomizedcontrolledtrial[Filter]) | 87 |
| **Embase**(December 1, 2025) | | |
| #1 | 'knee osteoarthritis'/exp | 60420 |
| #2 | 'knee osteoarthritis'/exp OR 'arthrosis, knee' OR 'femorotibial arthrosis' OR 'gonarthrosis' OR 'knee arthrosis' OR 'knee joint arthrosis' OR 'knee joint osteoarthritis' OR 'knee osteo-arthritis' OR 'knee osteo-arthrosis' OR 'knee osteoarthrosis' OR 'osteoarthritis, knee' OR 'osteoarthrosis, knee' OR 'knee osteoarthritis' | 64252 |
| #3 | 'alternative medicine'/exp | 95008 |
| #4 | 'alternative medicine'/exp OR 'alternative therapies' OR 'alternative therapy' OR 'complementary medicine' OR 'complementary therapies' OR 'eclectic medicine' OR 'eclecticism, historical' OR 'historical eclecticism' OR 'mental healing' OR 'metaphysical mind-body relations' OR 'mind body technique' OR 'mind body therapies' OR 'mind body therapy' OR 'mind-body relations (metaphysics)' OR 'mind-body relations, metaphysical' OR 'mind-body therapies' OR 'polarity therapy' OR 'radiaesthesia' OR 'radiesthesia' OR 'therapeutic cults' OR 'thomsonian medicine' OR 'alternative medicine' OR 'baduanjin' OR 'pilates' OR 'tai chi' OR 'yoga' OR 'tai ji quan' OR 'yi jin jing' OR 'qigong' OR 'wu qin xi' | 177849 |
| #5 | #2 AND #4 | 1835 |
| #6 | #5 AND 'randomized controlled trial'/de | 626 |
| **Cochrane**(December 1, 2025) | | |
| #1 | MeSH descriptor: [Osteoarthritis, Knee] explode all trees | 7534 |
| #2 | ("Osteoarthritis of Knee" OR "Knee Osteoarthritis" OR "Osteoarthritis of the Knee" OR "Knee Osteoarthritides"):ti,ab,kw | 14765 |
| #3 | #1 OR #2 | 17019 |
| #4 | MeSH descriptor: [Mind-Body Therapies] explode all trees | 10609 |
| #5 | ("Mind Body Therapies" OR "Mind-Body Therapy" OR "Therapy, Mind-Body" OR "Therapies, Mind-Body" OR "Mind-Body Medicine" OR "Mind Body Medicine" OR "Yoga" OR "Baduanjin" OR "Tai Chi" OR "Tai Chi Chuan" OR "Taijiquan" OR "Qigong" OR "Pilates" OR "Five Animal Frolics" OR "Wuqinxi" OR "Yijinjing"):ti,ab,kw | 12641 |
| #6 | #4 OR #5 | 20688 |
| #7 | #3 AND #6 | 318 |
| **Web of Science**(December 1, 2025) | | |
| #1 | TS=(osteoarthritis, knee OR "knee osteoarthritides" OR "knee osteoarthritis" OR "osteoarthritis of knee" OR "osteoarthritis of the knee") | 73455 |
| #2 | TS=("Mind-Body Therapies" OR "Mind Body Therapies" OR "Mind-Body Therapy" OR "Therapies, Mind-Body" OR "Therapy, Mind-Body" OR "Mind-Body Medicine" OR "Mind Body Medicine" OR "Yijinjing" OR "Qigong" OR "Taichi" OR "Tai Chi" OR "Tai Ji Quan" OR "Tai Chi Chuan" OR "Baduanjin" OR "Pilates" OR "Wuqinxi" OR "Five Animal Frolics" OR "Yoga") | 19723 |
| #3 | #1 AND #2 | 457 |
| #4 | TS=(random* OR randomi* OR randomly OR placebo* OR sham OR trial OR "clinical trial" OR "controlled clinical trial" OR "double blind" OR "single blind" OR "triple blind" OR crossover OR "cross over" OR "parallel group" OR cluster random* OR RCT) | 3995872 |
| #5 | #3 AND #4 | 335 |

**P-value Table for Each Outcome Measure**

**Table S3** Global inconsistency analysis p-values for each outcome measure

| **outcome measures** | **p-value of the global inconsistency analysis** |
| --- | --- |
| Mental health | 0.3957 |
| WOMAC-Pain | 0.9047 |
| WOMAC-Physical function | 0.6599 |
| WOMAC-Stiffness | 0.6944 |
| 6MWT | 0.7909 |

**Node Splitting Diagram**

| **Side** | **Coef.** | **Std. Err.** | **Coef.** | **Std. Err.** | **Coef.** | **Std. Err.** | **P>z** | **tau** |
| --- | --- | --- | --- | --- | --- | --- | --- | --- |
| BDJ VS NEI | -0.6453444 | 0.4505252 | -0.2128352 | 9.128964 | -0.4325092 | 9.140075 | 0.962 | 2.83e-07 |
| BDJ VS TC | 0.2102745 | 0.1735259 | -0.1394416 | 0.373312 | 0.3497161 | 0.4116703 | 0.396 | 4.10e-07 |
| CE VS YG | 0.0752735 | 0.3334693 | 0.424939 | 0.241452 | -0.3496655 | 0.4117048 | 0.396 | 2.83e-07 |
| CE VS YJJ | 1.792065 | 0.3674879 | 1.132093 | 63.26023 | 0.659973 | 63.26134 | 0.992 | 5.63e-10 |
| NEI VS TC | 0.1813409 | 0.1080881 | 0.5312108 | 0.3971309 | -0.3498699 | 0.4115775 | 0.395 | 1.19e-08 |
| NEI VS YG | 0.3960466 | 0.128487 | 0.0465911 | 0.3911078 | 0.3494555 | 0.4116724 | 0.396 | 3.27e-07 |

**Table S4** Node-splitting diagram for the outcome measure of Mental Health.

**Table S5** Node Splitting Diagram of the Outcome Measure WOMAC-Pain

| **Side** | **Coef.** | **Std. Err.** | **Coef.** | **Std. Err.** | **Coef.** | **Std. Err.** | **P>z** | **tau** |
| --- | --- | --- | --- | --- | --- | --- | --- | --- |
| BDJ VS NEI | 0.1986474 | 0.2663461 | 0.1400632 | 6.901851 | 0.0585842 | 6.906983 | 0.993 | 0.3225931 |
| CE VS PIL | -1.091218 | 0.3901141 | -1.306547 | 44.72835 | 0.2153293 | 44.72988 | 0.996 | 0.3225633 |
| CE VS TC | -0.8905451 | 0.2413472 | -0.8282273 | 0.4572908 | -0.0623178 | 0.5182512 | 0.904 | 0.3464755 |
| CE VS YG | -0.809757 | 0.3107736 | -0.8717907 | 0.4184748 | 0.0620336 | 0.5183602 | 0.905 | 0.3464817 |
| CE VS YJJ | -0.4840849 | 0.4474178 | -1.257509 | 63.25534 | 0.7734239 | 63.25696 | 0.99 | 0.3225621 |
| NEI VS TC | -0.4324078 | 0.1686322 | -0.494716 | 0.4892266 | 0.0623083 | 0.5179907 | 0.904 | 0.3464539 |
| NEI VS WQX | -0.4814438 | 0.2183607 | -0.3920801 | 36.52505 | -0.0893637 | 36.52572 | 0.998 | 0.3225615 |
| NEI VS YG | -0.4137492 | 0.2930528 | -0.3517835 | 0.4270002 | -0.0619658 | 0.5183045 | 0.905 | 0.3464767 |

**Table S6** Node Splitting Diagram of the Outcome Measure WOMAC-Physical Function

| **Side** | **Coef.** | **Std. Err.** | **Coef.** | **Std. Err.** | **Coef.** | **Std. Err.** | **P>z** | **tau** |
| --- | --- | --- | --- | --- | --- | --- | --- | --- |
| BDJ VS NEI | 0.9234779 | 0.2331334 | 0.2255263 | 7.072058 | 0.6979516 | 7.075908 | 0.921 | 0.2286971 |
| CE VS PIL | -1.295446 | 0.3543796 | -2.254577 | 44.73009 | 0.9591305 | 44.73145 | 0.983 | 0.2287031 |
| CE VS TC | -0.6726733 | 0.2000429 | -0.853752 | 0.36112 | 0.1810786 | 0.412867 | 0.661 | 0.2413821 |
| CE VS YG | -0.7088452 | 0.2503313 | -0.5269954 | 0.3307574 | -0.1818498 | 0.4129086 | 0.66 | 0.2413791 |
| CE VS YJJ | -0.0633915 | 0.3813543 | -2.212083 | 63.24025 | 2.148691 | 63.24143 | 0.973 | 0.2287029 |
| NEI VS TC | -0.5516508 | 0.1311795 | -0.3712991 | 0.3916978 | -0.1803517 | 0.4127519 | 0.662 | 0.2413786 |
| NEI VS WQX | -0.1485352 | 0.2296868 | -1.844157 | 44.69556 | 1.695621 | 44.69617 | 0.97 | 0.228704 |
| NEI VS YG | -0.4061839 | 0.2293738 | -0.5882587 | 0.3464712 | 0.1820748 | 0.4128758 | 0.659 | 0.2413744 |

**Table S7** Node Splitting Diagram of the Outcome Measure WOMAC-Stiffness

| **Side** | **Coef.** | **Std. Err.** | **Coef.** | **Std. Err.** | **Coef.** | **Std. Err.** | **P>z** | **tau** |
| --- | --- | --- | --- | --- | --- | --- | --- | --- |
| BDJ VS NEI | 1.654081 | 0.2757111 | 0.034145 | 7.255906 | 1.619936 | 7.26115 | 0.823 | 0.2956327 |
| CE VS PIL | -1.323498 | 0.6284458 | -4.333569 | 63.24874 | 3.010071 | 63.25158 | 0.962 | 0.2956917 |
| CE VS TC | -0.699013 | 0.2249846 | -0.5050541 | 0.4344906 | -0.1939589 | 0.4893159 | 0.692 | 0.3147385 |
| CE VS YG | -0.8258208 | 0.2975897 | -1.017736 | 0.3932079 | 0.1919153 | 0.4894215 | 0.695 | 0.3147588 |
| CE VS YJJ | -0.1361239 | 0.4251453 | -4.25269 | 63.25955 | 4.116566 | 63.26101 | 0.948 | 0.2956903 |
| NEI VS TC | -0.1574443 | 0.1725851 | -0.3532152 | 0.4576151 | 0.1957709 | 0.4890898 | 0.689 | 0.3147104 |
| NEI VS WQX | -0.273828 | 0.2050062 | -3.304053 | 36.50664 | 3.030225 | 36.5072 | 0.934 | 0.2956893 |
| NEI VS YG | -0.476917 | 0.2732249 | -0.2857731 | 0.411598 | -0.1911439 | 0.4893871 | 0.696 | 0.3147607 |

**Table S8** Node Splitting Diagram of the Outcome Measure 6MWT (6-Minute Walk Test)

| **Side** | **Coef.** | **Std. Err.** | **Coef.** | **Std. Err.** | **Coef.** | **Std. Err.** | **P>z** | **tau** |
| --- | --- | --- | --- | --- | --- | --- | --- | --- |
| CE VS NEI | -0.5286029 | 0.3211705 | -0.0350177 | 0.2988404 | -0.4935852 | 0.4389732 | 0.261 | 0.2223128 |
| CE VS TC | 0.0445824 | 0.2068747 | -0.4247251 | 0.4899819 | 0.4693075 | 0.5316569 | 0.377 | 0.2394941 |
| CE VS YG | 0.0615194 | 0.5140454 | 0.3972759 | 1.052803 | -0.3357565 | 1.162931 | 0.773 | 0.2706686 |
| NEI VS TC | 0.2001923 | 0.1887641 | 0.5840994 | 0.5212884 | -0.3839071 | 0.5545264 | 0.489 | 0.2538336 |
| NEI VS WQX | -0.0814043 | 0.2354383 | 0.5372564 | 44.72838 | -0.6186608 | 44.72901 | 0.989 | 0.2410059 |
| NEI VS YG | 0.4673843 | 0.526708 | 0.1316278 | 1.033856 | 0.3357565 | 1.162931 | 0.773 | 0.2706686 |

**SUCRA value**

**Table S9** SUCRA Value Table of the Outcome Measure TUG (Timed Up and Go)

| **Treatm~t** | **SUCRA** | **PrBest** | **MeanRank** |
| --- | --- | --- | --- |
| CE | 68.4 | 19.1 | 2.3 |
| NEI | 15.2 | 0.1 | 4.4 |
| TC | 42 | 4 | 3.3 |
| WQX | 36.5 | 7.4 | 3.5 |
| YG | 87.9 | 69.4 | 1.5 |

**Table S10** SUCRA Value Table of the Outcome Measure Mental Health

| **Treatm~t** | **SUCRA** | **PrBest** | **MeanRank** |
| --- | --- | --- | --- |
| BDJ | 67.5 | 2.3 | 2.6 |
| CE | 19.5 | 0 | 5 |
| NEI | 9.7 | 0 | 5.5 |
| TC | 42.7 | 0 | 3.9 |
| YG | 61.1 | 0 | 2.9 |
| YJJ | 99.5 | 97.7 | 1 |

**Table S11** SUCRA Value Table of the Outcome Measure WOMAC-Pain

| **Treatm~t** | **SUCRA** | **PrBest** | **MeanRank** |
| --- | --- | --- | --- |
| BDJ | 46.3 | 4.4 | 4.8 |
| CE | 3.1 | 0 | 7.8 |
| NEI | 25.6 | 0 | 6.2 |
| PIL | 80.3 | 50.4 | 2.4 |
| TC | 70.3 | 8.4 | 3.1 |
| WQX | 72.7 | 21.7 | 2.9 |
| YG | 63.6 | 8 | 3.5 |
| YJJ | 38 | 7 | 5.3 |

**Table S12** SUCRA Value Table of the Outcome Measure WOMAC-Physical Function

| **Treatm~t** | **SUCRA** | **PrBest** | **MeanRank** |
| --- | --- | --- | --- |
| BDJ | 88.4 | 34.1 | 1.8 |
| CE | 10.5 | 0 | 7.3 |
| NEI | 24.4 | 0 | 6.3 |
| PIL | 92.8 | 64.8 | 1.5 |
| TC | 66.7 | 0.3 | 3.3 |
| WQX | 36.4 | 0 | 5.5 |
| YG | 60 | 0.3 | 3.8 |
| YJJ | 20.8 | 0.4 | 6.5 |

**Table S13** SUCRA Value Table of the Outcome Measure WOMAC-Stiffness

| **Treatm~t** | **SUCRA** | **PrBest** | **MeanRank** |
| --- | --- | --- | --- |
| BDJ | 98 | 85.8 | 1.1 |
| CE | 5.9 | 0 | 7.6 |
| NEI | 29.6 | 0 | 5.9 |
| PIL | 75.8 | 14.2 | 2.7 |
| TC | 49.1 | 0 | 4.6 |
| WQX | 56.9 | 0 | 4 |
| YG | 66.8 | 0 | 3.3 |
| YJJ | 18.1 | 0 | 6.7 |

**Table S14** SUCRA Value Table of the Outcome Measure 6MWT (6-Minute Walk Test)

| **Treatm~t** | **SUCRA** | **PrBest** | **MeanRank** |
| --- | --- | --- | --- |
| CE | 66.6 | 22.0 | 2.3 |
| NEI | 26.0 | 0.8 | 4.0 |
| TC | 64.9 | 17.9 | 2.4 |
| WQX | 20.8 | 4.3 | 4.2 |
| YG | 71.7 | 55.0 | 2.1 |

**Sensitivity Analysis**

**Table S15** Sensitivity Analysis Using TUG(Timed-up-and-go) as the Outcome Measure.

| **dropped_id** | **comparison** | **eff** | **lci** | **uci** | **connected** |
| --- | --- | --- | --- | --- | --- |
| Michael Wortley 2013 | CE VS NEI | -0.57072158 | -1.56526 | 0.423817 | 1 |
| Michael Wortley 2013 | TC VS NEI | -0.34917979 | -0.9443078 | 0.2459483 | 1 |
| Michael Wortley 2013 | WQX VS NEI | -0.08750757 | -0.5562354 | 0.3812203 | 1 |
| Michael Wortley 2013 | YG VS NEI | -0.77825669 | -1.80027 | 0.2437563 | 1 |
| Chunmei Xiao 2020 | CE VS NEI | -0.57072158 | -1.440908 | 0.2994646 | 1 |
| Chunmei Xiao 2020 | TC VS NEI | -0.32555708 | -0.7601292 | 0.109015 | 1 |
| Chunmei Xiao 2020 | WQX VS NEI | -0.40220582 | -0.8826353 | 0.0782236 | 1 |
| Chunmei Xiao 2020 | YG VS NEI | -0.77825669 | -1.679715 | 0.1232019 | 1 |
| Alexander B. Kuntz 2018 | TC VS NEI | -0.32678303 | -0.8228726 | 0.1693066 | 1 |
| Alexander B. Kuntz 2018 | WQX VS NEI | -0.08404154 | -0.5102364 | 0.3421534 | 1 |
| Chun Mei Xiao 2020 | CE VS NEI | -0.57072158 | -1.440908 | 0.2994646 | 1 |
| Chun Mei Xiao 2020 | TC VS NEI | -0.32555708 | -0.7601292 | 0.109015 | 1 |
| Chun Mei Xiao 2020 | WQX VS NEI | 0.19419563 | -0.2327709 | 0.6211621 | 1 |
| Chun Mei Xiao 2020 | YG VS NEI | -0.77825669 | -1.679715 | 0.1232019 | 1 |
| Ning Kang 2022 | CE VS NEI | -0.57072158 | -1.575019 | 0.4335757 | 1 |
| Ning Kang 2022 | TC VS NEI | -0.26687414 | -0.9146729 | 0.3809246 | 1 |
| Ning Kang 2022 | WQX VS NEI | -0.08821028 | -0.5671995 | 0.390779 | 1 |
| Ning Kang 2022 | YG VS NEI | -0.77825669 | -1.809768 | 0.2532551 | 1 |
| Jiulong Song 2022 | CE VS NEI | -0.57072158 | -1.585949 | 0.4445057 | 1 |
| Jiulong Song 2022 | TC VS NEI | -0.36764276 | -1.082275 | 0.3469896 | 1 |
| Jiulong Song 2022 | WQX VS NEI | -0.0889366 | -0.5792856 | 0.4014125 | 1 |
| Jiulong Song 2022 | YG VS NEI | -0.77825669 | -1.820413 | 0.2638998 | 1 |

**Table S16** Sensitivity Analysis Using Mental health as the Outcome Measure.

| **dropped_id** | **comparison** | **eff** | **lci** | **uci** | **connected** |
| --- | --- | --- | --- | --- | --- |
| Pao-Feng Tsai 2012 | BDJ VS NEI | 0.64534438 | -0.237685 | 1.528374 | 1 |
| Pao-Feng Tsai 2012 | CE VS NEI | 0.0439563 | -0.358574 | 0.4464866 | 1 |
| Pao-Feng Tsai 2012 | TC VS NEI | 0.20674419 | 0.0014978 | 0.4119906 | 1 |
| Pao-Feng Tsai 2012 | YG VS NEI | 0.36052973 | 0.1198905 | 0.6011689 | 1 |
| Pao-Feng Tsai 2012 | YJJ VS NEI | 1.8359554 | 1.010857 | 2.661054 | 1 |
| BINGCHEN AN 2008 | CE VS NEI | 0.05737947 | -0.2904066 | 0.4051655 | 1 |
| BINGCHEN AN 2008 | TC VS NEI | 0.20551575 | 0.0011097 | 0.4099218 | 1 |
| BINGCHEN AN 2008 | YG VS NEI | 0.36226499 | 0.1230551 | 0.6014749 | 1 |
| BINGCHEN AN 2008 | YJJ VS NEI | 1.8493776 | 1.049554 | 2.649201 | 1 |
| Chenchen Wang 2016 | BDJ VS NEI | 0.64534438 | -0.237685 | 1.528374 | 1 |
| Chenchen Wang 2016 | CE VS NEI | 0.15300519 | -0.2404341 | 0.5464444 | 1 |
| Chenchen Wang 2016 | TC VS NEI | 0.32369718 | 0.0179725 | 0.6294219 | 1 |
| Chenchen Wang 2016 | YG VS NEI | 0.3746214 | 0.1342334 | 0.6150094 | 1 |
| Chenchen Wang 2016 | YJJ VS NEI | 1.9449968 | 1.124294 | 2.765699 | 1 |
| Augustine C. Lee 2017 | BDJ VS NEI | 0.64534438 | -0.237685 | 1.528374 | 1 |
| Augustine C. Lee 2017 | CE VS NEI | 0.03467504 | -0.3227789 | 0.392129 | 1 |
| Augustine C. Lee 2017 | TC VS NEI | 0.17745592 | -0.0510083 | 0.4059201 | 1 |
| Augustine C. Lee 2017 | YG VS NEI | 0.35933119 | 0.1198834 | 0.598779 | 1 |
| Augustine C. Lee 2017 | YJJ VS NEI | 1.8266748 | 1.022601 | 2.630749 | 1 |
| Ning Kang 2022 | BDJ VS NEI | 0.64534438 | -0.237685 | 1.528374 | 1 |
| Ning Kang 2022 | CE VS NEI | 0.07134546 | -0.2795014 | 0.4221924 | 1 |
| Ning Kang 2022 | TC VS NEI | 0.22277598 | 0.0105307 | 0.4350213 | 1 |
| Ning Kang 2022 | YG VS NEI | 0.36406961 | 0.1247851 | 0.6033542 | 1 |
| Ning Kang 2022 | YJJ VS NEI | 1.8633427 | 1.062183 | 2.664502 | 1 |
| Subhadra Evans 2013 | BDJ VS NEI | 0.64534438 | -0.237685 | 1.528374 | 1 |
| Subhadra Evans 2013 | CE VS NEI | 0.05695663 | -0.2914555 | 0.4053687 | 1 |
| Subhadra Evans 2013 | TC VS NEI | 0.20539756 | 0.0009082 | 0.4098869 | 1 |
| Subhadra Evans 2013 | YG VS NEI | 0.36071696 | 0.1095915 | 0.6118425 | 1 |
| Subhadra Evans 2013 | YJJ VS NEI | 1.8489548 | 1.048859 | 2.649051 | 1 |
| Corjena Cheung 2016 | BDJ VS NEI | 0.64534438 | -0.237685 | 1.528374 | 1 |
| Corjena Cheung 2016 | CE VS NEI | 0.03057608 | -0.3185916 | 0.3797437 | 1 |
| Corjena Cheung 2016 | TC VS NEI | 0.19802406 | -0.0065659 | 0.4026141 | 1 |
| Corjena Cheung 2016 | YG VS NEI | 0.26413405 | -0.0006841 | 0.5289522 | 1 |
| Corjena Cheung 2016 | YJJ VS NEI | 1.8225761 | 1.022151 | 2.623002 | 1 |
| CHENCHEN WANG 2009 | BDJ VS NEI | 0.64534438 | -0.237685 | 1.528374 | 1 |
| CHENCHEN WANG 2009 | CE VS NEI | 0.00933981 | -0.3743689 | 0.3930485 | 1 |
| CHENCHEN WANG 2009 | TC VS NEI | 0.20991285 | 0.0049685 | 0.4148572 | 1 |
| CHENCHEN WANG 2009 | YG VS NEI | 0.35605748 | 0.1159322 | 0.5961828 | 1 |
| CHENCHEN WANG 2009 | YJJ VS NEI | 1.8013413 | 0.9852582 | 2.617424 | 1 |
| Hwa-Jin Lee 2009 | BDJ VS NEI | 0.64534438 | -0.237685 | 1.528374 | 1 |
| Hwa-Jin Lee 2009 | CE VS NEI | 0.17741819 | -0.2040087 | 0.5588451 | 1 |
| Hwa-Jin Lee 2009 | TC VS NEI | 0.19453395 | -0.0103748 | 0.3994427 | 1 |
| Hwa-Jin Lee 2009 | YG VS NEI | 0.37777596 | 0.1377114 | 0.6178405 | 1 |
| Hwa-Jin Lee 2009 | YJJ VS NEI | 1.9694081 | 1.154395 | 2.784421 | 1 |
| Jiulong Song 2022 | BDJ VS NEI | 0.64534438 | -0.237685 | 1.528374 | 1 |
| Jiulong Song 2022 | CE VS NEI | 0.0357142 | -0.316706 | 0.3881344 | 1 |
| Jiulong Song 2022 | TC VS NEI | 0.17874027 | -0.0374495 | 0.3949301 | 1 |
| Jiulong Song 2022 | YG VS NEI | 0.35946514 | 0.120142 | 0.5987883 | 1 |
| Jiulong Song 2022 | YJJ VS NEI | 1.8277139 | 1.025864 | 2.629563 | 1 |
| Shuaipan Zhang 2022 | BDJ VS NEI | 0.64534438 | -0.237685 | 1.528374 | 1 |
| Shuaipan Zhang 2022 | CE VS NEI | 0.05741014 | -0.2903813 | 0.4052016 | 1 |
| Shuaipan Zhang 2022 | TC VS NEI | 0.20552433 | 0.0011175 | 0.4099311 | 1 |
| Shuaipan Zhang 2022 | YG VS NEI | 0.36226896 | 0.1230589 | 0.601479 | 1 |
| Corjena Cheung 2014 | BDJ VS NEI | 0.64534438 | -0.237685 | 1.528374 | 1 |
| Corjena Cheung 2014 | CE VS NEI | -0.02890361 | -0.4295751 | 0.3717678 | 1 |
| Corjena Cheung 2014 | TC VS NEI | 0.18139924 | -0.0304359 | 0.3932344 | 1 |
| Corjena Cheung 2014 | YG VS NEI | 0.39634029 | 0.1445591 | 0.6481215 | 1 |
| Corjena Cheung 2014 | YJJ VS NEI | 1.7631005 | 0.9389069 | 2.587294 | 1 |
| Shiyi Julia Zhu 2025 | BDJ VS NEI | 0.64534438 | -0.237685 | 1.528374 | 1 |
| Shiyi Julia Zhu 2025 | CE VS NEI | 0.09441228 | -0.2634964 | 0.4523209 | 1 |
| Shiyi Julia Zhu 2025 | TC VS NEI | 0.21586617 | 0.0100995 | 0.4216328 | 1 |
| Shiyi Julia Zhu 2025 | YG VS NEI | 0.49784894 | 0.1067317 | 0.8889661 | 1 |
| Shiyi Julia Zhu 2025 | YJJ VS NEI | 1.8864079 | 1.082131 | 2.690684 | 1 |

**Table S17** Sensitivity Analysis Using WOMAC-Pain as the Outcome Measure.

| **dropped_id** | **comparison** | **eff** | **lci** | **uci** | **connected** |
| --- | --- | --- | --- | --- | --- |
| Pao-Feng Tsai 2012 | BDJ VS NEI | -0.20337523 | -0.741238 | 0.3344875 | 1 |
| Pao-Feng Tsai 2012 | CE VS NEI | 0.47726689 | -0.0307762 | 0.98531 | 1 |
| Pao-Feng Tsai 2012 | PIL VS NEI | -0.62323613 | -1.553913 | 0.3074409 | 1 |
| Pao-Feng Tsai 2012 | TC VS NEI | -0.44580843 | -0.7564108 | -0.135206 | 1 |
| Pao-Feng Tsai 2012 | WQX VS NEI | -0.48005572 | -0.9263796 | -0.0337319 | 1 |
| Pao-Feng Tsai 2012 | YG VS NEI | -0.37454306 | -0.8510392 | 0.1019531 | 1 |
| Pao-Feng Tsai 2012 | YJJ VS NEI | -0.00684266 | -1.043403 | 1.029718 | 1 |
| BINGCHEN AN 2008 | BDJ VS NEI | 0.009129 | -0.5617321 | 0.57999 | 1 |
| BINGCHEN AN 2008 | CE VS NEI | 0.43415878 | -0.0067517 | 0.8750693 | 1 |
| BINGCHEN AN 2008 | PIL VS NEI | -0.64903395 | -1.516668 | 0.2186005 | 1 |
| BINGCHEN AN 2008 | TC VS NEI | -0.43945924 | -0.7287841 | -0.1501344 | 1 |
| BINGCHEN AN 2008 | WQX VS NEI | -0.48285489 | -0.895339 | -0.0703708 | 1 |
| BINGCHEN AN 2008 | YG VS NEI | -0.38299981 | -0.8203973 | 0.0543977 | 1 |
| BINGCHEN AN 2008 | YJJ VS NEI | -0.04994405 | -1.011666 | 0.9117783 | 1 |
| Chenchen Wang 2016 | BDJ VS NEI | -0.20379371 | -0.743136 | 0.3355485 | 1 |
| Chenchen Wang 2016 | CE VS NEI | 0.40822556 | -0.0756377 | 0.8920888 | 1 |
| Chenchen Wang 2016 | PIL VS NEI | -0.69309777 | -1.611536 | 0.2253402 | 1 |
| Chenchen Wang 2016 | TC VS NEI | -0.47787557 | -0.8210126 | -0.1347386 | 1 |
| Chenchen Wang 2016 | WQX VS NEI | -0.47993596 | -0.9279256 | -0.0319463 | 1 |
| Chenchen Wang 2016 | YG VS NEI | -0.4071589 | -0.8792742 | 0.0649564 | 1 |
| Chenchen Wang 2016 | YJJ VS NEI | -0.07587667 | -1.102949 | 0.951196 | 1 |
| Nadia Saleem 2022 | BDJ VS NEI | -0.19473958 | -0.7043417 | 0.3148625 | 1 |
| Nadia Saleem 2022 | CE VS NEI | 0.43425427 | -0.0074724 | 0.875981 | 1 |
| Nadia Saleem 2022 | PIL VS NEI | -1.6139075 | -3.070774 | -0.1570406 | 1 |
| Nadia Saleem 2022 | TC VS NEI | -0.43945835 | -0.7293538 | -0.1495629 | 1 |
| Nadia Saleem 2022 | WQX VS NEI | -0.48276334 | -0.8962108 | -0.0693159 | 1 |
| Nadia Saleem 2022 | YG VS NEI | -0.38332257 | -0.8214413 | 0.0547961 | 1 |
| Nadia Saleem 2022 | YJJ VS NEI | -0.04984863 | -1.01318 | 0.9134823 | 1 |
| Kim L. Bennell 2022 | BDJ VS NEI | -0.18700428 | -0.6744093 | 0.3004007 | 1 |
| Kim L. Bennell 2022 | CE VS NEI | 0.28190837 | -0.1751544 | 0.7389711 | 1 |
| Kim L. Bennell 2022 | PIL VS NEI | -0.78727752 | -1.636691 | 0.0621358 | 1 |
| Kim L. Bennell 2022 | TC VS NEI | -0.486894 | -0.7662277 | -0.2075603 | 1 |
| Kim L. Bennell 2022 | WQX VS NEI | -0.48564364 | -0.8718172 | -0.09947 | 1 |
| Kim L. Bennell 2022 | YG VS NEI | -0.68409887 | -1.225133 | -0.1430646 | 1 |
| Kim L. Bennell 2022 | YJJ VS NEI | -0.20217954 | -1.138757 | 0.7343982 | 1 |
| Tiago Albuquerque Maranhao Rego 2023 | BDJ VS NEI | -0.19473944 | -0.7043411 | 0.3148623 | 1 |
| Tiago Albuquerque Maranhao Rego 2023 | CE VS NEI | 0.434223 | -0.0075025 | 0.8759485 | 1 |
| Tiago Albuquerque Maranhao Rego 2023 | PIL VS NEI | -0.262091 | -1.246599 | 0.7224174 | 1 |
| Tiago Albuquerque Maranhao Rego 2023 | TC VS NEI | -0.43946843 | -0.7293636 | -0.1495733 | 1 |
| Tiago Albuquerque Maranhao Rego 2023 | WQX VS NEI | -0.48276338 | -0.8962103 | -0.0693165 | 1 |
| Tiago Albuquerque Maranhao Rego 2023 | YG VS NEI | -0.38333678 | -0.8214568 | 0.0547832 | 1 |
| Tiago Albuquerque Maranhao Rego 2023 | YJJ VS NEI | -0.0498799 | -1.01321 | 0.9134499 | 1 |
| Michael Wortley 2013 | BDJ VS NEI | -0.20095431 | -0.7306381 | 0.3287295 | 1 |
| Michael Wortley 2013 | CE VS NEI | 0.41934224 | -0.0456672 | 0.8843516 | 1 |
| Michael Wortley 2013 | PIL VS NEI | -0.6763766 | -1.577626 | 0.2248726 | 1 |
| Michael Wortley 2013 | TC VS NEI | -0.46190864 | -0.7762173 | -0.1475999 | 1 |
| Michael Wortley 2013 | WQX VS NEI | -0.48076798 | -0.9176116 | -0.0439243 | 1 |
| Michael Wortley 2013 | YG VS NEI | -0.39843136 | -0.8593396 | 0.0624769 | 1 |
| Michael Wortley 2013 | YJJ VS NEI | -0.06476054 | -1.068665 | 0.9391443 | 1 |
| Augustine C. Lee 2017 | BDJ VS NEI | -0.20477339 | -0.7474213 | 0.3378745 | 1 |
| Augustine C. Lee 2017 | CE VS NEI | 0.43688562 | -0.0468689 | 0.9206401 | 1 |
| Augustine C. Lee 2017 | PIL VS NEI | -0.66639354 | -1.588937 | 0.2561503 | 1 |
| Augustine C. Lee 2017 | TC VS NEI | -0.44042239 | -0.7781581 | -0.1026867 | 1 |
| Augustine C. Lee 2017 | WQX VS NEI | -0.47965941 | -0.9315579 | -0.0277609 | 1 |
| Augustine C. Lee 2017 | YG VS NEI | -0.39501951 | -0.87035 | 0.080311 | 1 |
| Augustine C. Lee 2017 | YJJ VS NEI | -0.04721995 | -1.07931 | 0.9848704 | 1 |
| Qingguang Zhu 2016 | BDJ VS NEI | -0.20310814 | -0.7400712 | 0.3338549 | 1 |
| Qingguang Zhu 2016 | CE VS NEI | 0.45411502 | -0.0221529 | 0.930383 | 1 |
| Qingguang Zhu 2016 | PIL VS NEI | -0.64585495 | -1.558654 | 0.2669437 | 1 |
| Qingguang Zhu 2016 | TC VS NEI | -0.41681901 | -0.7455867 | -0.0880513 | 1 |
| Qingguang Zhu 2016 | WQX VS NEI | -0.48013277 | -0.9254011 | -0.0348645 | 1 |
| Qingguang Zhu 2016 | YG VS NEI | -0.38497961 | -0.8536112 | 0.083652 | 1 |
| Qingguang Zhu 2016 | YJJ VS NEI | -0.02999196 | -1.049992 | 0.9900077 | 1 |
| Xiaoyue Hu 2020 | BDJ VS NEI | -0.18935658 | -0.6835788 | 0.3048656 | 1 |
| Xiaoyue Hu 2020 | CE VS NEI | 0.50364954 | 0.0689608 | 0.9383383 | 1 |
| Xiaoyue Hu 2020 | PIL VS NEI | -0.56992029 | -1.420906 | 0.281065 | 1 |
| Xiaoyue Hu 2020 | TC VS NEI | -0.34409393 | -0.6470916 | -0.0410962 | 1 |
| Xiaoyue Hu 2020 | WQX VS NEI | -0.48471531 | -0.8790434 | -0.0903872 | 1 |
| Xiaoyue Hu 2020 | YG VS NEI | -0.34393071 | -0.7684349 | 0.0805735 | 1 |
| Xiaoyue Hu 2020 | YJJ VS NEI | 0.01954147 | -0.916397 | 0.95548 | 1 |
| Chunmei Xiao 2020 | BDJ VS NEI | -0.20243296 | -0.7371917 | 0.3323258 | 1 |
| Chunmei Xiao 2020 | CE VS NEI | 0.43676885 | -0.0293343 | 0.902872 | 1 |
| Chunmei Xiao 2020 | PIL VS NEI | -0.66186296 | -1.567655 | 0.243929 | 1 |
| Chunmei Xiao 2020 | TC VS NEI | -0.43946918 | -0.7463683 | -0.13257 | 1 |
| Chunmei Xiao 2020 | WQX VS NEI | -0.57696854 | -1.111698 | -0.0422388 | 1 |
| Chunmei Xiao 2020 | YG VS NEI | -0.39220583 | -0.8570508 | 0.0726391 | 1 |
| Chunmei Xiao 2020 | YJJ VS NEI | -0.04733611 | -1.059184 | 0.9645116 | 1 |
| Chun Mei Xiao 2020 | BDJ VS NEI | -0.20499776 | -0.7484239 | 0.3384284 | 1 |
| Chun Mei Xiao 2020 | CE VS NEI | 0.43772666 | -0.0369081 | 0.9123614 | 1 |
| Chun Mei Xiao 2020 | PIL VS NEI | -0.66600079 | -1.584575 | 0.252573 | 1 |
| Chun Mei Xiao 2020 | TC VS NEI | -0.4394187 | -0.7522753 | -0.1265621 | 1 |
| Chun Mei Xiao 2020 | WQX VS NEI | -0.48571012 | -1.024651 | 0.0532303 | 1 |
| Chun Mei Xiao 2020 | YG VS NEI | -0.39489862 | -0.8689017 | 0.0791045 | 1 |
| Chun Mei Xiao 2020 | YJJ VS NEI | -0.04637907 | -1.075403 | 0.9826449 | 1 |
| Jean-Michel Brisme´e 2007 | BDJ VS NEI | -0.19476694 | -0.7046301 | 0.3150962 | 1 |
| Jean-Michel Brisme´e 2007 | CE VS NEI | 0.37872149 | -0.0695806 | 0.8270236 | 1 |
| Jean-Michel Brisme´e 2007 | PIL VS NEI | -0.70502421 | -1.579009 | 0.1689605 | 1 |
| Jean-Michel Brisme´e 2007 | TC VS NEI | -0.51362008 | -0.8218446 | -0.2053956 | 1 |
| Jean-Michel Brisme´e 2007 | WQX VS NEI | -0.48275398 | -0.896327 | -0.069181 | 1 |
| Jean-Michel Brisme´e 2007 | YG VS NEI | -0.40892973 | -0.8501524 | 0.0322929 | 1 |
| Jean-Michel Brisme´e 2007 | YJJ VS NEI | -0.10537606 | -1.071866 | 0.8611137 | 1 |
| Corjena Cheung 2016 | BDJ VS NEI | -0.17924491 | -0.6453907 | 0.2869009 | 1 |
| Corjena Cheung 2016 | CE VS NEI | 0.54854965 | 0.1359362 | 0.9611632 | 1 |
| Corjena Cheung 2016 | PIL VS NEI | -0.50662484 | -1.317338 | 0.3040886 | 1 |
| Corjena Cheung 2016 | TC VS NEI | -0.40200599 | -0.6629194 | -0.1410926 | 1 |
| Corjena Cheung 2016 | WQX VS NEI | -0.48909258 | -0.849387 | -0.1287981 | 1 |
| Corjena Cheung 2016 | YG VS NEI | -0.12115391 | -0.5654467 | 0.3231389 | 1 |
| Corjena Cheung 2016 | YJJ VS NEI | 0.06444048 | -0.8196182 | 0.9484991 | 1 |
| JiaJia Ye 2020 | BDJ VS NEI | -0.36461388 | -1.052363 | 0.3231348 | 1 |
| JiaJia Ye 2020 | CE VS NEI | 0.43672208 | -0.0289367 | 0.9023809 | 1 |
| JiaJia Ye 2020 | PIL VS NEI | -0.66165522 | -1.566595 | 0.2432846 | 1 |
| JiaJia Ye 2020 | TC VS NEI | -0.43947109 | -0.7460749 | -0.1328673 | 1 |
| JiaJia Ye 2020 | WQX VS NEI | -0.48036671 | -0.9224656 | -0.0382679 | 1 |
| JiaJia Ye 2020 | YG VS NEI | -0.39206721 | -0.8562686 | 0.0721342 | 1 |
| JiaJia Ye 2020 | YJJ VS NEI | -0.04738284 | -1.058372 | 0.9636059 | 1 |
| Guo-Xin Ni 2010 | BDJ VS NEI | -0.18652052 | -0.6714181 | 0.298377 | 1 |
| Guo-Xin Ni 2010 | CE VS NEI | 0.27832439 | -0.1597892 | 0.716438 | 1 |
| Guo-Xin Ni 2010 | PIL VS NEI | -0.7899712 | -1.637373 | 0.0574306 | 1 |
| Guo-Xin Ni 2010 | TC VS NEI | -0.41422743 | -0.687949 | -0.1405058 | 1 |
| Guo-Xin Ni 2010 | WQX VS NEI | -0.48584078 | -0.8701167 | -0.1015649 | 1 |
| Guo-Xin Ni 2010 | YG VS NEI | -0.44245795 | -0.8632843 | -0.0216316 | 1 |
| Guo-Xin Ni 2010 | YJJ VS NEI | -0.20576319 | -1.13121 | 0.7196841 | 1 |
| CHENCHEN WANG 2009 | BDJ VS NEI | -0.20267681 | -0.7381656 | 0.332812 | 1 |
| CHENCHEN WANG 2009 | CE VS NEI | 0.48143881 | -0.0183742 | 0.9812518 | 1 |
| CHENCHEN WANG 2009 | PIL VS NEI | -0.61768166 | -1.541087 | 0.3057237 | 1 |
| CHENCHEN WANG 2009 | TC VS NEI | -0.44652729 | -0.75527 | -0.1377846 | 1 |
| CHENCHEN WANG 2009 | WQX VS NEI | -0.48025776 | -0.9238247 | -0.0366908 | 1 |
| CHENCHEN WANG 2009 | YG VS NEI | -0.37175699 | -0.8440527 | 0.1005387 | 1 |
| CHENCHEN WANG 2009 | YJJ VS NEI | -0.00267097 | -1.031701 | 1.026359 | 1 |
| Hwa-Jin Lee 2009 | BDJ VS NEI | -0.20021934 | -0.7275177 | 0.327079 | 1 |
| Hwa-Jin Lee 2009 | CE VS NEI | 0.51767967 | 0.0259717 | 1.009388 | 1 |
| Hwa-Jin Lee 2009 | PIL VS NEI | -0.57661436 | -1.487579 | 0.3343503 | 1 |
| Hwa-Jin Lee 2009 | TC VS NEI | -0.45247681 | -0.7556424 | -0.1493113 | 1 |
| Hwa-Jin Lee 2009 | WQX VS NEI | -0.4809911 | -0.9150061 | -0.0469761 | 1 |
| Hwa-Jin Lee 2009 | YG VS NEI | -0.35192837 | -0.815199 | 0.1113422 | 1 |
| Hwa-Jin Lee 2009 | YJJ VS NEI | 0.03356691 | -0.9793814 | 1.046515 | 1 |
| Jiulong Song 2022 | BDJ VS NEI | -0.20309891 | -0.7400277 | 0.3338298 | 1 |
| Jiulong Song 2022 | CE VS NEI | 0.45073985 | -0.0249699 | 0.9264496 | 1 |
| Jiulong Song 2022 | PIL VS NEI | -0.64921133 | -1.561674 | 0.2632518 | 1 |
| Jiulong Song 2022 | TC VS NEI | -0.42128312 | -0.7486831 | -0.0938832 | 1 |
| Jiulong Song 2022 | WQX VS NEI | -0.48013527 | -0.9253668 | -0.0349037 | 1 |
| Jiulong Song 2022 | YG VS NEI | -0.38653725 | -0.8550217 | 0.0819472 | 1 |
| Jiulong Song 2022 | YJJ VS NEI | -0.03336677 | -1.053059 | 0.9863253 | 1 |
| Shuaipan Zhang 2022 | BDJ VS NEI | -0.19874779 | -0.7211074 | 0.3236118 | 1 |
| Shuaipan Zhang 2022 | CE VS NEI | 0.43549804 | -0.0186931 | 0.8896891 | 1 |
| Shuaipan Zhang 2022 | PIL VS NEI | -0.65591654 | -1.543424 | 0.2315906 | 1 |
| Shuaipan Zhang 2022 | TC VS NEI | -0.43949468 | -0.7380977 | -0.1408916 | 1 |
| Shuaipan Zhang 2022 | WQX VS NEI | -0.48144771 | -0.9098309 | -0.0530645 | 1 |
| Shuaipan Zhang 2022 | YG VS NEI | -0.38810587 | -0.8397492 | 0.0635375 | 1 |
| Jiajia Ye 2020 | BDJ VS NEI | -0.31119018 | -0.9953801 | 0.3729998 | 1 |
| Jiajia Ye 2020 | CE VS NEI | 0.43716102 | -0.0324493 | 0.9067713 | 1 |
| Jiajia Ye 2020 | PIL VS NEI | -0.66358331 | -1.574506 | 0.2473391 | 1 |
| Jiajia Ye 2020 | TC VS NEI | -0.43945106 | -0.7488096 | -0.1300925 | 1 |
| Jiajia Ye 2020 | WQX VS NEI | -0.48001996 | -0.9268335 | -0.0332064 | 1 |
| Jiajia Ye 2020 | YG VS NEI | -0.39334116 | -0.8618419 | 0.0751596 | 1 |
| Jiajia Ye 2020 | YJJ VS NEI | -0.04694425 | -1.065865 | 0.9719766 | 1 |
| Corjena Cheung 2014 | BDJ VS NEI | -0.20018801 | -0.7275122 | 0.3271362 | 1 |
| Corjena Cheung 2014 | CE VS NEI | 0.39140475 | -0.0871105 | 0.86992 | 1 |
| Corjena Cheung 2014 | PIL VS NEI | -0.70280935 | -1.607376 | 0.2017568 | 1 |
| Corjena Cheung 2014 | TC VS NEI | -0.45404333 | -0.7590635 | -0.1490231 | 1 |
| Corjena Cheung 2014 | WQX VS NEI | -0.48100066 | -0.91491 | -0.0470914 | 1 |
| Corjena Cheung 2014 | YG VS NEI | -0.34725908 | -0.8212938 | 0.1267756 | 1 |
| Corjena Cheung 2014 | YJJ VS NEI | -0.09269497 | -1.099153 | 0.9137635 | 1 |
| Zhigang Xiao 2021 | BDJ VS NEI | -0.20293991 | -0.7394028 | 0.333523 | 1 |
| Zhigang Xiao 2021 | CE VS NEI | 0.43695402 | -0.0308193 | 0.9047273 | 1 |
| Zhigang Xiao 2021 | PIL VS NEI | -0.6626802 | -1.570985 | 0.245625 | 1 |
| Zhigang Xiao 2021 | TC VS NEI | -0.4394611 | -0.7475255 | -0.1313967 | 1 |
| Zhigang Xiao 2021 | WQX VS NEI | -0.36400484 | -0.93386 | 0.2058503 | 1 |
| Zhigang Xiao 2021 | YG VS NEI | -0.39274803 | -0.8593932 | 0.0738972 | 1 |
| Zhigang Xiao 2021 | YJJ VS NEI | -0.04715109 | -1.062352 | 0.9680497 | 1 |
| Bedru J. Abafita 2025 | BDJ VS NEI | -0.20397778 | -0.7437821 | 0.3358265 | 1 |
| Bedru J. Abafita 2025 | CE VS NEI | 0.50435761 | -0.0068467 | 1.015562 | 1 |
| Bedru J. Abafita 2025 | PIL VS NEI | -0.59734651 | -1.531586 | 0.3368932 | 1 |
| Bedru J. Abafita 2025 | TC VS NEI | -0.41740732 | -0.7346524 | -0.1001622 | 1 |
| Bedru J. Abafita 2025 | WQX VS NEI | -0.47988353 | -0.9285895 | -0.0311775 | 1 |
| Bedru J. Abafita 2025 | YG VS NEI | -0.4587495 | -0.9685914 | 0.0510924 | 1 |
| Bedru J. Abafita 2025 | YJJ VS NEI | 0.02024495 | -1.02092 | 1.06141 | 1 |

**Table S18** Sensitivity Analysis Using WOMAC-Physical function as the Outcome Measure.

| **dropped_id** | **comparison** | **eff** | **lci** | **uci** | **connected** |
| --- | --- | --- | --- | --- | --- |
| Pao-Feng Tsai 2012 | BDJ VS NEI | -0.92273587 | -1.390184 | -0.4552875 | 1 |
| Pao-Feng Tsai 2012 | CE VS NEI | 0.18155011 | -0.2356912 | 0.5987914 | 1 |
| Pao-Feng Tsai 2012 | PIL VS NEI | -1.1204166 | -1.944595 | -0.2962385 | 1 |
| Pao-Feng Tsai 2012 | TC VS NEI | -0.53379833 | -0.7801421 | -0.2874546 | 1 |
| Pao-Feng Tsai 2012 | WQX VS NEI | -0.14932365 | -0.6144137 | 0.3157664 | 1 |
| Pao-Feng Tsai 2012 | YG VS NEI | -0.46162291 | -0.8494136 | -0.0738322 | 1 |
| Pao-Feng Tsai 2012 | YJJ VS NEI | 0.1181471 | -0.7536088 | 0.989903 | 1 |
| BINGCHEN AN 2008 | BDJ VS NEI | -0.96270708 | -1.48327 | -0.4421444 | 1 |
| BINGCHEN AN 2008 | CE VS NEI | 0.18253817 | -0.1971414 | 0.5622177 | 1 |
| BINGCHEN AN 2008 | PIL VS NEI | -1.1164006 | -1.916878 | -0.3159233 | 1 |
| BINGCHEN AN 2008 | TC VS NEI | -0.533262 | -0.7746683 | -0.2918557 | 1 |
| BINGCHEN AN 2008 | WQX VS NEI | -0.14898814 | -0.6071895 | 0.3092132 | 1 |
| BINGCHEN AN 2008 | YG VS NEI | -0.45859143 | -0.8337072 | -0.0834757 | 1 |
| BINGCHEN AN 2008 | YJJ VS NEI | 0.11913533 | -0.7278268 | 0.9660975 | 1 |
| Chenchen Wang 2016 | BDJ VS NEI | -0.92339725 | -1.381472 | -0.4653223 | 1 |
| Chenchen Wang 2016 | CE VS NEI | 0.14060928 | -0.2438828 | 0.5251014 | 1 |
| Chenchen Wang 2016 | PIL VS NEI | -1.1554832 | -1.953281 | -0.3576856 | 1 |
| Chenchen Wang 2016 | TC VS NEI | -0.59129222 | -0.8569388 | -0.3256456 | 1 |
| Chenchen Wang 2016 | WQX VS NEI | -0.1486609 | -0.6004363 | 0.3031145 | 1 |
| Chenchen Wang 2016 | YG VS NEI | -0.47480849 | -0.8471488 | -0.1024682 | 1 |
| Chenchen Wang 2016 | YJJ VS NEI | 0.07720978 | -0.7650164 | 0.919436 | 1 |
| Nadia Saleem 2022 | BDJ VS NEI | -0.92383836 | -1.375812 | -0.4718646 | 1 |
| Nadia Saleem 2022 | CE VS NEI | 0.18527039 | -0.1834291 | 0.5539699 | 1 |
| Nadia Saleem 2022 | PIL VS NEI | -2.0508649 | -3.470071 | -0.6316587 | 1 |
| Nadia Saleem 2022 | TC VS NEI | -0.53192741 | -0.7650258 | -0.298829 | 1 |
| Nadia Saleem 2022 | WQX VS NEI | -0.14819664 | -0.5912717 | 0.2948785 | 1 |
| Nadia Saleem 2022 | YG VS NEI | -0.45127248 | -0.8138743 | -0.0886707 | 1 |
| Nadia Saleem 2022 | YJJ VS NEI | 0.12186788 | -0.7039383 | 0.9476741 | 1 |
| Kim L. Bennell 2022 | BDJ VS NEI | -0.9248047 | -1.363889 | -0.4857204 | 1 |
| Kim L. Bennell 2022 | CE VS NEI | 0.05574561 | -0.3302128 | 0.441704 | 1 |
| Kim L. Bennell 2022 | PIL VS NEI | -1.2282139 | -2.005021 | -0.4514069 | 1 |
| Kim L. Bennell 2022 | TC VS NEI | -0.56804485 | -0.7952783 | -0.3408114 | 1 |
| Kim L. Bennell 2022 | WQX VS NEI | -0.14711134 | -0.5716842 | 0.2774616 | 1 |
| Kim L. Bennell 2022 | YG VS NEI | -0.71529821 | -1.180279 | -0.2503179 | 1 |
| Kim L. Bennell 2022 | YJJ VS NEI | -0.00764746 | -0.8218686 | 0.8065737 | 1 |
| Tiago Albuquerque Maranhao Rego 2023 | BDJ VS NEI | -0.92383832 | -1.375813 | -0.471864 | 1 |
| Tiago Albuquerque Maranhao Rego 2023 | CE VS NEI | 0.18525046 | -0.1834508 | 0.5539517 | 1 |
| Tiago Albuquerque Maranhao Rego 2023 | PIL VS NEI | -0.79311941 | -1.665191 | 0.078952 | 1 |
| Tiago Albuquerque Maranhao Rego 2023 | TC VS NEI | -0.53193329 | -0.7650325 | -0.2988341 | 1 |
| Tiago Albuquerque Maranhao Rego 2023 | WQX VS NEI | -0.14819668 | -0.5912725 | 0.2948792 | 1 |
| Tiago Albuquerque Maranhao Rego 2023 | YG VS NEI | -0.45128165 | -0.8138856 | -0.0886777 | 1 |
| Tiago Albuquerque Maranhao Rego 2023 | YJJ VS NEI | 0.12184796 | -0.7039599 | 0.9476558 | 1 |
| Michael Wortley 2013 | BDJ VS NEI | -0.92319838 | -1.384051 | -0.4623457 | 1 |
| Michael Wortley 2013 | CE VS NEI | 0.16961402 | -0.2112069 | 0.5504349 | 1 |
| Michael Wortley 2013 | PIL VS NEI | -1.1282349 | -1.92824 | -0.3282302 | 1 |
| Michael Wortley 2013 | TC VS NEI | -0.55118254 | -0.798201 | -0.3041641 | 1 |
| Michael Wortley 2013 | WQX VS NEI | -0.14886426 | -0.6045957 | 0.3068672 | 1 |
| Michael Wortley 2013 | YG VS NEI | -0.46342397 | -0.8378565 | -0.0889915 | 1 |
| Michael Wortley 2013 | YJJ VS NEI | 0.10621224 | -0.7386043 | 0.9510288 | 1 |
| Augustine C. Lee 2017 | BDJ VS NEI | -0.9227352 | -1.390192 | -0.4552787 | 1 |
| Augustine C. Lee 2017 | CE VS NEI | 0.16341967 | -0.2282081 | 0.5550475 | 1 |
| Augustine C. Lee 2017 | PIL VS NEI | -1.1385507 | -1.949981 | -0.3271199 | 1 |
| Augustine C. Lee 2017 | TC VS NEI | -0.55816311 | -0.8221464 | -0.2941798 | 1 |
| Augustine C. Lee 2017 | WQX VS NEI | -0.1493243 | -0.6144264 | 0.3157779 | 1 |
| Augustine C. Lee 2017 | YG VS NEI | -0.46978255 | -0.8521649 | -0.0874003 | 1 |
| Augustine C. Lee 2017 | YJJ VS NEI | 0.10001806 | -0.7597892 | 0.9598253 | 1 |
| Xiaoyue Hu 2020 | BDJ VS NEI | -0.9305665 | -1.302429 | -0.5587039 | 1 |
| Xiaoyue Hu 2020 | CE VS NEI | 0.30721893 | 0.0235959 | 0.590842 | 1 |
| Xiaoyue Hu 2020 | PIL VS NEI | -0.93182934 | -1.597121 | -0.2665377 | 1 |
| Xiaoyue Hu 2020 | TC VS NEI | -0.39655382 | -0.5655533 | -0.2275543 | 1 |
| Xiaoyue Hu 2020 | WQX VS NEI | -0.1377859 | -0.462507 | 0.1869352 | 1 |
| Xiaoyue Hu 2020 | YG VS NEI | -0.32101633 | -0.600124 | -0.0419087 | 1 |
| Xiaoyue Hu 2020 | YJJ VS NEI | 0.24381409 | -0.4237301 | 0.9113582 | 1 |
| Chunmei Xiao 2020 | BDJ VS NEI | -0.92323123 | -1.383626 | -0.4628361 | 1 |
| Chunmei Xiao 2020 | CE VS NEI | 0.18306434 | -0.1944003 | 0.5605289 | 1 |
| Chunmei Xiao 2020 | PIL VS NEI | -1.114496 | -1.911921 | -0.3170715 | 1 |
| Chunmei Xiao 2020 | TC VS NEI | -0.53300969 | -0.7727259 | -0.2932934 | 1 |
| Chunmei Xiao 2020 | WQX VS NEI | -0.36009606 | -1.023149 | 0.3029566 | 1 |
| Chunmei Xiao 2020 | YG VS NEI | -0.45714364 | -0.8300925 | -0.0841947 | 1 |
| Chunmei Xiao 2020 | YJJ VS NEI | 0.11966158 | -0.7229393 | 0.9622625 | 1 |
| Chun Mei Xiao 2020 | BDJ VS NEI | -0.92323122 | -1.383626 | -0.462836 | 1 |
| Chun Mei Xiao 2020 | CE VS NEI | 0.18306435 | -0.1944003 | 0.5605289 | 1 |
| Chun Mei Xiao 2020 | PIL VS NEI | -1.1144961 | -1.911921 | -0.3170715 | 1 |
| Chun Mei Xiao 2020 | TC VS NEI | -0.53300968 | -0.7727259 | -0.2932934 | 1 |
| Chun Mei Xiao 2020 | WQX VS NEI | 0.03917227 | -0.5863125 | 0.664657 | 1 |
| Chun Mei Xiao 2020 | YG VS NEI | -0.45714369 | -0.8300926 | -0.0841948 | 1 |
| Chun Mei Xiao 2020 | YJJ VS NEI | 0.11966158 | -0.7229393 | 0.9622625 | 1 |
| Jean-Michel Brisme´e 2007 | BDJ VS NEI | -0.92297671 | -1.386971 | -0.4589825 | 1 |
| Jean-Michel Brisme´e 2007 | CE VS NEI | 0.16375963 | -0.2225345 | 0.5500538 | 1 |
| Jean-Michel Brisme´e 2007 | PIL VS NEI | -1.1360545 | -1.941801 | -0.3303079 | 1 |
| Jean-Michel Brisme´e 2007 | TC VS NEI | -0.55843508 | -0.8143052 | -0.3025649 | 1 |
| Jean-Michel Brisme´e 2007 | WQX VS NEI | -0.14908676 | -0.6092803 | 0.3111068 | 1 |
| Jean-Michel Brisme´e 2007 | YG VS NEI | -0.46777904 | -0.8462155 | -0.0893425 | 1 |
| Jean-Michel Brisme´e 2007 | YJJ VS NEI | 0.10035815 | -0.7517346 | 0.9524509 | 1 |
| Corjena Cheung 2016 | BDJ VS NEI | -0.92476 | -1.364436 | -0.4850842 | 1 |
| Corjena Cheung 2016 | CE VS NEI | 0.2566701 | -0.1106988 | 0.624039 | 1 |
| Corjena Cheung 2016 | PIL VS NEI | -1.0276899 | -1.799713 | -0.2556666 | 1 |
| Corjena Cheung 2016 | TC VS NEI | -0.51061573 | -0.7351125 | -0.2861189 | 1 |
| Corjena Cheung 2016 | WQX VS NEI | -0.14716375 | -0.572589 | 0.2782615 | 1 |
| Corjena Cheung 2016 | YG VS NEI | -0.30297408 | -0.6959132 | 0.089965 | 1 |
| Corjena Cheung 2016 | YJJ VS NEI | 0.19326335 | -0.6131986 | 0.9997253 | 1 |
| JiaJia Ye 2020 | BDJ VS NEI | -0.89291451 | -1.498522 | -0.287307 | 1 |
| JiaJia Ye 2020 | CE VS NEI | 0.18169002 | -0.2018115 | 0.5651915 | 1 |
| JiaJia Ye 2020 | PIL VS NEI | -1.119574 | -1.925674 | -0.3134741 | 1 |
| JiaJia Ye 2020 | TC VS NEI | -0.53366338 | -0.7779487 | -0.289378 | 1 |
| JiaJia Ye 2020 | WQX VS NEI | -0.14924697 | -0.6127357 | 0.3142417 | 1 |
| JiaJia Ye 2020 | YG VS NEI | -0.46096839 | -0.8403107 | -0.0816261 | 1 |
| JiaJia Ye 2020 | YJJ VS NEI | 0.11828706 | -0.7361016 | 0.9726757 | 1 |
| Guo-Xin Ni 2010 | BDJ VS NEI | -0.92296869 | -1.387078 | -0.4588597 | 1 |
| Guo-Xin Ni 2010 | CE VS NEI | 0.1504389 | -0.2534848 | 0.5543626 | 1 |
| Guo-Xin Ni 2010 | PIL VS NEI | -1.1494449 | -1.963994 | -0.3348956 | 1 |
| Guo-Xin Ni 2010 | TC VS NEI | -0.52860613 | -0.771939 | -0.2852732 | 1 |
| Guo-Xin Ni 2010 | WQX VS NEI | -0.14909473 | -0.6094509 | 0.3112614 | 1 |
| Guo-Xin Ni 2010 | YG VS NEI | -0.47381958 | -0.8562625 | -0.0913767 | 1 |
| Guo-Xin Ni 2010 | YJJ VS NEI | 0.08703843 | -0.7733616 | 0.9474385 | 1 |
| CHENCHEN WANG 2009 | BDJ VS NEI | -0.92289226 | -1.388094 | -0.4576902 | 1 |
| CHENCHEN WANG 2009 | CE VS NEI | 0.1995276 | -0.2085783 | 0.6076335 | 1 |
| CHENCHEN WANG 2009 | PIL VS NEI | -1.1010432 | -1.918163 | -0.2839234 | 1 |
| CHENCHEN WANG 2009 | TC VS NEI | -0.53622323 | -0.7806013 | -0.2918452 | 1 |
| CHENCHEN WANG 2009 | WQX VS NEI | -0.14917039 | -0.6110766 | 0.3127358 | 1 |
| CHENCHEN WANG 2009 | YG VS NEI | -0.45236628 | -0.8358378 | -0.0688948 | 1 |
| CHENCHEN WANG 2009 | YJJ VS NEI | 0.13612333 | -0.7278977 | 1.000144 | 1 |
| Hwa-Jin Lee 2009 | BDJ VS NEI | -0.92318381 | -1.384245 | -0.4621224 | 1 |
| Hwa-Jin Lee 2009 | CE VS NEI | 0.2328157 | -0.1708028 | 0.6364343 | 1 |
| Hwa-Jin Lee 2009 | PIL VS NEI | -1.0651698 | -1.875635 | -0.2547051 | 1 |
| Hwa-Jin Lee 2009 | TC VS NEI | -0.54068226 | -0.7818889 | -0.2994756 | 1 |
| Hwa-Jin Lee 2009 | WQX VS NEI | -0.14887902 | -0.6049058 | 0.3071478 | 1 |
| Hwa-Jin Lee 2009 | YG VS NEI | -0.43523143 | -0.8134812 | -0.0569817 | 1 |
| Hwa-Jin Lee 2009 | YJJ VS NEI | 0.16940916 | -0.6862348 | 1.025053 | 1 |
| Jiulong Song 2022 | BDJ VS NEI | -0.92315507 | -1.384622 | -0.4616877 | 1 |
| Jiulong Song 2022 | CE VS NEI | 0.2022958 | -0.1798803 | 0.5844719 | 1 |
| Jiulong Song 2022 | PIL VS NEI | -1.0959403 | -1.895876 | -0.2960048 | 1 |
| Jiulong Song 2022 | TC VS NEI | -0.50666766 | -0.7586571 | -0.2546782 | 1 |
| Jiulong Song 2022 | WQX VS NEI | -0.14890808 | -0.6055118 | 0.3076957 | 1 |
| Jiulong Song 2022 | YG VS NEI | -0.44912249 | -0.8231181 | -0.0751269 | 1 |
| Jiulong Song 2022 | YJJ VS NEI | 0.13889153 | -0.7074708 | 0.9852538 | 1 |
| Shuaipan Zhang 2022 | BDJ VS NEI | -0.92345937 | -1.380658 | -0.4662608 | 1 |
| Shuaipan Zhang 2022 | CE VS NEI | 0.18386937 | -0.1902386 | 0.5579774 | 1 |
| Shuaipan Zhang 2022 | PIL VS NEI | -1.1116829 | -1.903821 | -0.3195452 | 1 |
| Shuaipan Zhang 2022 | TC VS NEI | -0.53262026 | -0.7698222 | -0.2954183 | 1 |
| Shuaipan Zhang 2022 | WQX VS NEI | -0.14859662 | -0.5991275 | 0.3019342 | 1 |
| Shuaipan Zhang 2022 | YG VS NEI | -0.4549781 | -0.8236941 | -0.0862621 | 1 |
| Jiajia Ye 2020 | BDJ VS NEI | -0.90068426 | -1.493258 | -0.3081107 | 1 |
| Jiajia Ye 2020 | CE VS NEI | 0.18170983 | -0.2016996 | 0.5651193 | 1 |
| Jiajia Ye 2020 | PIL VS NEI | -1.1194984 | -1.925462 | -0.3135353 | 1 |
| Jiajia Ye 2020 | TC VS NEI | -0.53365407 | -0.7778702 | -0.2894379 | 1 |
| Jiajia Ye 2020 | WQX VS NEI | -0.14924086 | -0.6126025 | 0.3141207 | 1 |
| Jiajia Ye 2020 | YG VS NEI | -0.46091232 | -0.8401514 | -0.0816733 | 1 |
| Jiajia Ye 2020 | YJJ VS NEI | 0.11830687 | -0.7359029 | 0.9725167 | 1 |
| Corjena Cheung 2014 | BDJ VS NEI | -0.92345721 | -1.38069 | -0.4662242 | 1 |
| Corjena Cheung 2014 | CE VS NEI | 0.13695115 | -0.2519456 | 0.5258479 | 1 |
| Corjena Cheung 2014 | PIL VS NEI | -1.1586143 | -1.958677 | -0.3585514 | 1 |
| Corjena Cheung 2014 | TC VS NEI | -0.5465143 | -0.7860873 | -0.3069413 | 1 |
| Corjena Cheung 2014 | WQX VS NEI | -0.14859887 | -0.5991773 | 0.3019796 | 1 |
| Corjena Cheung 2014 | YG VS NEI | -0.4141853 | -0.7911183 | -0.0372523 | 1 |
| Corjena Cheung 2014 | YJJ VS NEI | 0.07355195 | -0.7694252 | 0.9165291 | 1 |
| Shiyi Julia Zhu 2025 | BDJ VS NEI | -0.92231626 | -1.395881 | -0.4487519 | 1 |
| Shiyi Julia Zhu 2025 | CE VS NEI | 0.17545511 | -0.2252758 | 0.576186 | 1 |
| Shiyi Julia Zhu 2025 | PIL VS NEI | -1.1302935 | -1.951927 | -0.3086601 | 1 |
| Shiyi Julia Zhu 2025 | TC VS NEI | -0.54073475 | -0.8173482 | -0.2641212 | 1 |
| Shiyi Julia Zhu 2025 | WQX VS NEI | -0.14972474 | -0.6234646 | 0.3240151 | 1 |
| Shiyi Julia Zhu 2025 | YG VS NEI | -0.46746998 | -0.8568856 | -0.0780544 | 1 |
| Shiyi Julia Zhu 2025 | YJJ VS NEI | 0.11205227 | -0.7612507 | 0.9853553 | 1 |
| Bedru J. Abafita 2025 | BDJ VS NEI | -0.922479 | -1.393655 | -0.4513032 | 1 |
| Bedru J. Abafita 2025 | CE VS NEI | 0.20654827 | -0.2196351 | 0.6327317 | 1 |
| Bedru J. Abafita 2025 | PIL VS NEI | -1.0977316 | -1.928741 | -0.2667228 | 1 |
| Bedru J. Abafita 2025 | TC VS NEI | -0.52630691 | -0.7798319 | -0.2727819 | 1 |
| Bedru J. Abafita 2025 | WQX VS NEI | -0.1495709 | -0.6199363 | 0.3207945 | 1 |
| Bedru J. Abafita 2025 | YG VS NEI | -0.48720619 | -0.9062472 | -0.0681652 | 1 |
| Bedru J. Abafita 2025 | YJJ VS NEI | 0.14314311 | -0.7385291 | 1.024815 | 1 |

**Table S19** Sensitivity Analysis Using WOMAC-Stiffness as the Outcome Measure.

| **dropped_id** | **comparison** | **eff** | **lci** | **uci** | **connected** |
| --- | --- | --- | --- | --- | --- |
| Pao-Feng Tsai 2012 | BDJ VS NEI | -1.6609329 | -2.179682 | -1.142184 | 1 |
| Pao-Feng Tsai 2012 | CE VS NEI | 0.34321551 | -0.0978985 | 0.7843295 | 1 |
| Pao-Feng Tsai 2012 | PIL VS NEI | -0.98023221 | -2.262507 | 0.3020425 | 1 |
| Pao-Feng Tsai 2012 | TC VS NEI | -0.15599234 | -0.4438136 | 0.1318289 | 1 |
| Pao-Feng Tsai 2012 | WQX VS NEI | -0.27929005 | -0.6520758 | 0.0934957 | 1 |
| Pao-Feng Tsai 2012 | YG VS NEI | -0.45695476 | -0.8712741 | -0.0426355 | 1 |
| Pao-Feng Tsai 2012 | YJJ VS NEI | 0.20706901 | -0.6993047 | 1.113443 | 1 |
| BINGCHEN AN 2008 | BDJ VS NEI | -1.9999061 | -2.588427 | -1.411385 | 1 |
| BINGCHEN AN 2008 | CE VS NEI | 0.47595797 | 0.0728033 | 0.8791127 | 1 |
| BINGCHEN AN 2008 | PIL VS NEI | -0.84751403 | -2.108214 | 0.4131864 | 1 |
| BINGCHEN AN 2008 | TC VS NEI | -0.18118353 | -0.4606861 | 0.098319 | 1 |
| BINGCHEN AN 2008 | WQX VS NEI | -0.2816182 | -0.6436887 | 0.0804523 | 1 |
| BINGCHEN AN 2008 | YG VS NEI | -0.39175222 | -0.7876635 | 0.0041591 | 1 |
| BINGCHEN AN 2008 | YJJ VS NEI | 0.3398017 | -0.5357792 | 1.215383 | 1 |
| Chenchen Wang 2016 | BDJ VS NEI | -1.6480976 | -2.209348 | -1.086847 | 1 |
| Chenchen Wang 2016 | CE VS NEI | 0.46376987 | -0.0144334 | 0.9419731 | 1 |
| Chenchen Wang 2016 | PIL VS NEI | -0.85969673 | -2.206029 | 0.4866355 | 1 |
| Chenchen Wang 2016 | TC VS NEI | -0.19869909 | -0.5627462 | 0.1653481 | 1 |
| Chenchen Wang 2016 | WQX VS NEI | -0.26988754 | -0.6988113 | 0.1590362 | 1 |
| Chenchen Wang 2016 | YG VS NEI | -0.42803361 | -0.8881211 | 0.0320539 | 1 |
| Chenchen Wang 2016 | YJJ VS NEI | 0.32760651 | -0.6673379 | 1.322551 | 1 |
| Kim L. Bennell 2022 | BDJ VS NEI | -1.6539183 | -2.194948 | -1.112889 | 1 |
| Kim L. Bennell 2022 | CE VS NEI | 0.37108205 | -0.101916 | 0.8440802 | 1 |
| Kim L. Bennell 2022 | PIL VS NEI | -0.95236842 | -2.272333 | 0.3675963 | 1 |
| Kim L. Bennell 2022 | TC VS NEI | -0.22021019 | -0.5324613 | 0.0920409 | 1 |
| Kim L. Bennell 2022 | WQX VS NEI | -0.27373858 | -0.6762739 | 0.1287968 | 1 |
| Kim L. Bennell 2022 | YG VS NEI | -0.62865475 | -1.193658 | -0.0636518 | 1 |
| Kim L. Bennell 2022 | YJJ VS NEI | 0.23493054 | -0.7240286 | 1.19389 | 1 |
| Tiago Albuquerque Maranhao Rego 2023 | BDJ VS NEI | -1.6540342 | -2.194584 | -1.113485 | 1 |
| Tiago Albuquerque Maranhao Rego 2023 | CE VS NEI | 0.47635871 | 0.0390146 | 0.9137028 | 1 |
| Tiago Albuquerque Maranhao Rego 2023 | TC VS NEI | -0.18155821 | -0.4865662 | 0.1234498 | 1 |
| Tiago Albuquerque Maranhao Rego 2023 | WQX VS NEI | -0.27382148 | -0.6757826 | 0.1281396 | 1 |
| Tiago Albuquerque Maranhao Rego 2023 | YG VS NEI | -0.41122492 | -0.8432424 | 0.0207926 | 1 |
| Tiago Albuquerque Maranhao Rego 2023 | YJJ VS NEI | 0.34019762 | -0.6010416 | 1.281437 | 1 |
| Michael Wortley 2013 | BDJ VS NEI | -1.6519477 | -2.199624 | -1.104271 | 1 |
| Michael Wortley 2013 | CE VS NEI | 0.462593 | 0.0124114 | 0.9127746 | 1 |
| Michael Wortley 2013 | PIL VS NEI | -0.86087429 | -2.180901 | 0.4591521 | 1 |
| Michael Wortley 2013 | TC VS NEI | -0.19996232 | -0.5230381 | 0.1231135 | 1 |
| Michael Wortley 2013 | WQX VS NEI | -0.27236724 | -0.6836463 | 0.1389118 | 1 |
| Michael Wortley 2013 | YG VS NEI | -0.4214839 | -0.8639583 | 0.0209905 | 1 |
| Michael Wortley 2013 | YJJ VS NEI | 0.32643202 | -0.6326119 | 1.285476 | 1 |
| Qingguang Zhu 2016 | BDJ VS NEI | -1.6516903 | -2.200292 | -1.103089 | 1 |
| Qingguang Zhu 2016 | CE VS NEI | 0.50989607 | 0.0536558 | 0.9661363 | 1 |
| Qingguang Zhu 2016 | PIL VS NEI | -0.81358022 | -2.136758 | 0.5095977 | 1 |
| Qingguang Zhu 2016 | TC VS NEI | -0.13748198 | -0.4731523 | 0.1981884 | 1 |
| Qingguang Zhu 2016 | WQX VS NEI | -0.27219332 | -0.6846545 | 0.1402679 | 1 |
| Qingguang Zhu 2016 | YG VS NEI | -0.40039737 | -0.8435707 | 0.042776 | 1 |
| Qingguang Zhu 2016 | YJJ VS NEI | 0.37373046 | -0.5896467 | 1.337108 | 1 |
| Xiaoyue Hu 2020 | BDJ VS NEI | -1.6489632 | -2.207109 | -1.090817 | 1 |
| Xiaoyue Hu 2020 | CE VS NEI | 0.46195989 | -0.0091088 | 0.9330286 | 1 |
| Xiaoyue Hu 2020 | PIL VS NEI | -0.86150657 | -2.201504 | 0.4784907 | 1 |
| Xiaoyue Hu 2020 | TC VS NEI | -0.20101649 | -0.5542181 | 0.1521851 | 1 |
| Xiaoyue Hu 2020 | WQX VS NEI | -0.27042345 | -0.6953226 | 0.1544757 | 1 |
| Xiaoyue Hu 2020 | YG VS NEI | -0.42730185 | -0.8832611 | 0.0286574 | 1 |
| Xiaoyue Hu 2020 | YJJ VS NEI | 0.32579723 | -0.660557 | 1.312151 | 1 |
| Chunmei Xiao 2020 | BDJ VS NEI | -1.6552672 | -2.192143 | -1.118391 | 1 |
| Chunmei Xiao 2020 | CE VS NEI | 0.47628522 | 0.0435751 | 0.9089953 | 1 |
| Chunmei Xiao 2020 | PIL VS NEI | -0.84718536 | -2.147878 | 0.4535073 | 1 |
| Chunmei Xiao 2020 | TC VS NEI | -0.18154235 | -0.4831159 | 0.1200312 | 1 |
| Chunmei Xiao 2020 | WQX VS NEI | -0.41470175 | -0.8912312 | 0.0618277 | 1 |
| Chunmei Xiao 2020 | YG VS NEI | -0.40884784 | -0.8379301 | 0.0202344 | 1 |
| Chunmei Xiao 2020 | YJJ VS NEI | 0.34012481 | -0.5921251 | 1.272375 | 1 |
| Chun Mei Xiao 2020 | BDJ VS NEI | -1.6488271 | -2.207477 | -1.090177 | 1 |
| Chun Mei Xiao 2020 | CE VS NEI | 0.47678164 | 0.0192506 | 0.9343127 | 1 |
| Chun Mei Xiao 2020 | PIL VS NEI | -0.84668768 | -2.182586 | 0.4892107 | 1 |
| Chun Mei Xiao 2020 | TC VS NEI | -0.18151289 | -0.501449 | 0.1384232 | 1 |
| Chun Mei Xiao 2020 | WQX VS NEI | -0.28976389 | -0.7949317 | 0.2154039 | 1 |
| Chun Mei Xiao 2020 | YG VS NEI | -0.42076021 | -0.8743738 | 0.0328534 | 1 |
| Chun Mei Xiao 2020 | YJJ VS NEI | 0.34061744 | -0.6401598 | 1.321395 | 1 |
| Jean-Michel Brisme´e 2007 | BDJ VS NEI | -1.6532329 | -2.196549 | -1.109917 | 1 |
| Jean-Michel Brisme´e 2007 | CE VS NEI | 0.43379856 | -0.0157024 | 0.8832995 | 1 |
| Jean-Michel Brisme´e 2007 | PIL VS NEI | -0.88966356 | -2.204167 | 0.4248396 | 1 |
| Jean-Michel Brisme´e 2007 | TC VS NEI | -0.23799019 | -0.56767 | 0.0916896 | 1 |
| Jean-Michel Brisme´e 2007 | WQX VS NEI | -0.27325321 | -0.6788058 | 0.1322994 | 1 |
| Jean-Michel Brisme´e 2007 | YG VS NEI | -0.43213194 | -0.8708852 | 0.0066213 | 1 |
| Jean-Michel Brisme´e 2007 | YJJ VS NEI | 0.29764096 | -0.6537858 | 1.249068 | 1 |
| Corjena Cheung 2016 | BDJ VS NEI | -1.6697831 | -2.163492 | -1.176074 | 1 |
| Corjena Cheung 2016 | CE VS NEI | 0.5817807 | 0.1861596 | 0.9774018 | 1 |
| Corjena Cheung 2016 | PIL VS NEI | -0.74171027 | -1.979261 | 0.4958401 | 1 |
| Corjena Cheung 2016 | TC VS NEI | -0.14357734 | -0.4093525 | 0.1221978 | 1 |
| Corjena Cheung 2016 | WQX VS NEI | -0.28824992 | -0.6271553 | 0.0506554 | 1 |
| Corjena Cheung 2016 | YG VS NEI | -0.16474079 | -0.5843944 | 0.2549128 | 1 |
| Corjena Cheung 2016 | YJJ VS NEI | 0.44561945 | -0.3962834 | 1.287522 | 1 |
| JiaJia Ye 2020 | BDJ VS NEI | -1.4814998 | -2.181614 | -0.7813854 | 1 |
| JiaJia Ye 2020 | CE VS NEI | 0.47653394 | 0.0304863 | 0.9225816 | 1 |
| JiaJia Ye 2020 | PIL VS NEI | -0.84693597 | -2.166376 | 0.4725043 | 1 |
| JiaJia Ye 2020 | TC VS NEI | -0.18155573 | -0.4930145 | 0.129903 | 1 |
| JiaJia Ye 2020 | WQX VS NEI | -0.27223499 | -0.6843882 | 0.1399183 | 1 |
| JiaJia Ye 2020 | YG VS NEI | -0.41549614 | -0.8569136 | 0.0259213 | 1 |
| JiaJia Ye 2020 | YJJ VS NEI | 0.34037152 | -0.617865 | 1.298608 | 1 |
| Guo-Xin Ni 2010 | BDJ VS NEI | -1.6503627 | -2.203565 | -1.09716 | 1 |
| Guo-Xin Ni 2010 | CE VS NEI | 0.46723424 | -0.0097428 | 0.9442114 | 1 |
| Guo-Xin Ni 2010 | PIL VS NEI | -0.85623357 | -2.192284 | 0.4798166 | 1 |
| Guo-Xin Ni 2010 | TC VS NEI | -0.17977211 | -0.4965482 | 0.137004 | 1 |
| Guo-Xin Ni 2010 | WQX VS NEI | -0.2713158 | -0.6897866 | 0.147155 | 1 |
| Guo-Xin Ni 2010 | YG VS NEI | -0.4223274 | -0.8751817 | 0.030527 | 1 |
| Guo-Xin Ni 2010 | YJJ VS NEI | 0.33107191 | -0.6499131 | 1.312057 | 1 |
| CHENCHEN WANG 2009 | BDJ VS NEI | -1.6528543 | -2.197461 | -1.108248 | 1 |
| CHENCHEN WANG 2009 | CE VS NEI | 0.54722441 | 0.0771404 | 1.017308 | 1 |
| CHENCHEN WANG 2009 | PIL VS NEI | -0.77625917 | -2.099477 | 0.5469587 | 1 |
| CHENCHEN WANG 2009 | TC VS NEI | -0.19487985 | -0.5046816 | 0.1149219 | 1 |
| CHENCHEN WANG 2009 | WQX VS NEI | -0.27298902 | -0.6802295 | 0.1342515 | 1 |
| CHENCHEN WANG 2009 | YG VS NEI | -0.38123162 | -0.8234615 | 0.0609983 | 1 |
| CHENCHEN WANG 2009 | YJJ VS NEI | 0.41105605 | -0.5523768 | 1.374489 | 1 |
| Hwa-Jin Lee 2009 | BDJ VS NEI | -1.6507043 | -2.202707 | -1.098702 | 1 |
| Hwa-Jin Lee 2009 | CE VS NEI | 0.51179324 | 0.0333739 | 0.9902126 | 1 |
| Hwa-Jin Lee 2009 | PIL VS NEI | -0.81168326 | -2.1468 | 0.523433 | 1 |
| Hwa-Jin Lee 2009 | TC VS NEI | -0.18814317 | -0.5040785 | 0.1277922 | 1 |
| Hwa-Jin Lee 2009 | WQX VS NEI | -0.2715387 | -0.68845 | 0.1453726 | 1 |
| Hwa-Jin Lee 2009 | YG VS NEI | -0.40134359 | -0.8527665 | 0.0500793 | 1 |
| Hwa-Jin Lee 2009 | YJJ VS NEI | 0.37562682 | -0.6040857 | 1.355339 | 1 |
| Jiulong Song 2022 | BDJ VS NEI | -1.6518323 | -2.199937 | -1.103728 | 1 |
| Jiulong Song 2022 | CE VS NEI | 0.50881831 | 0.0538828 | 0.9637538 | 1 |
| Jiulong Song 2022 | PIL VS NEI | -0.8146578 | -2.136793 | 0.5074775 | 1 |
| Jiulong Song 2022 | TC VS NEI | -0.13888432 | -0.4723187 | 0.19455 | 1 |
| Jiulong Song 2022 | WQX VS NEI | -0.27228916 | -0.6841062 | 0.1395278 | 1 |
| Jiulong Song 2022 | YG VS NEI | -0.40062552 | -0.8428925 | 0.0416415 | 1 |
| Jiulong Song 2022 | YJJ VS NEI | 0.37265289 | -0.5892915 | 1.334597 | 1 |
| Shuaipan Zhang 2022 | BDJ VS NEI | -1.6540341 | -2.194584 | -1.113485 | 1 |
| Shuaipan Zhang 2022 | CE VS NEI | 0.47638628 | 0.0390419 | 0.9137307 | 1 |
| Shuaipan Zhang 2022 | PIL VS NEI | -0.84708408 | -2.154234 | 0.460066 | 1 |
| Shuaipan Zhang 2022 | TC VS NEI | -0.18154809 | -0.4865562 | 0.12346 | 1 |
| Shuaipan Zhang 2022 | WQX VS NEI | -0.27382153 | -0.6757828 | 0.1281397 | 1 |
| Shuaipan Zhang 2022 | YG VS NEI | -0.41121236 | -0.8432279 | 0.0208031 | 1 |
| Jiajia Ye 2020 | BDJ VS NEI | -1.4178879 | -2.091434 | -0.744342 | 1 |
| Jiajia Ye 2020 | CE VS NEI | 0.47638946 | 0.037775 | 0.915004 | 1 |
| Jiajia Ye 2020 | PIL VS NEI | -0.84708083 | -2.156024 | 0.461862 | 1 |
| Jiajia Ye 2020 | TC VS NEI | -0.18155752 | -0.4875132 | 0.1243982 | 1 |
| Jiajia Ye 2020 | WQX VS NEI | -0.2735796 | -0.6770493 | 0.1298901 | 1 |
| Jiajia Ye 2020 | YG VS NEI | -0.411866 | -0.8453258 | 0.0215938 | 1 |
| Jiajia Ye 2020 | YJJ VS NEI | 0.34022817 | -0.6034998 | 1.283956 | 1 |
| Corjena Cheung 2014 | BDJ VS NEI | -1.657564 | -2.186851 | -1.128277 | 1 |
| Corjena Cheung 2014 | CE VS NEI | 0.3994985 | -0.0417461 | 0.8407431 | 1 |
| Corjena Cheung 2014 | PIL VS NEI | -0.92395841 | -2.218657 | 0.3707398 | 1 |
| Corjena Cheung 2014 | TC VS NEI | -0.20936838 | -0.5080159 | 0.0892792 | 1 |
| Corjena Cheung 2014 | WQX VS NEI | -0.27648264 | -0.6633791 | 0.1104138 | 1 |
| Corjena Cheung 2014 | YG VS NEI | -0.33787434 | -0.7652496 | 0.0895009 | 1 |
| Corjena Cheung 2014 | YJJ VS NEI | 0.26334597 | -0.6605218 | 1.187214 | 1 |
| Zhigang Xiao 2021 | BDJ VS NEI | -1.6560028 | -2.190544 | -1.121461 | 1 |
| Zhigang Xiao 2021 | CE VS NEI | 0.47624059 | 0.0462635 | 0.9062177 | 1 |
| Zhigang Xiao 2021 | PIL VS NEI | -0.84723012 | -2.144133 | 0.4496725 | 1 |
| Zhigang Xiao 2021 | TC VS NEI | -0.18152986 | -0.4810728 | 0.118013 | 1 |
| Zhigang Xiao 2021 | WQX VS NEI | -0.08667509 | -0.5963035 | 0.4229533 | 1 |
| Zhigang Xiao 2021 | YG VS NEI | -0.40740935 | -0.834001 | 0.0191823 | 1 |
| Zhigang Xiao 2021 | YJJ VS NEI | 0.34008058 | -0.5868734 | 1.267035 | 1 |
| Bedru J. Abafita 2025 | BDJ VS NEI | -1.6566119 | -2.188774 | -1.12445 | 1 |
| Bedru J. Abafita 2025 | CE VS NEI | 0.62125749 | 0.1497333 | 1.092782 | 1 |
| Bedru J. Abafita 2025 | PIL VS NEI | -0.70224016 | -2.011164 | 0.6066834 | 1 |
| Bedru J. Abafita 2025 | TC VS NEI | -0.12863198 | -0.4350758 | 0.1778118 | 1 |
| Bedru J. Abafita 2025 | WQX VS NEI | -0.2757384 | -0.6665501 | 0.1150733 | 1 |
| Bedru J. Abafita 2025 | YG VS NEI | -0.53262598 | -0.9912512 | -0.0740008 | 1 |
| Bedru J. Abafita 2025 | YJJ VS NEI | 0.48508504 | -0.4586186 | 1.428789 | 1 |

**Table S20** Sensitivity Analysis Using 6MWT（6-min walk test）as the Outcome Measure.

| **dropped_id** | **comparison** | **eff** | **lci** | **uci** | **connected** |
| --- | --- | --- | --- | --- | --- |
| Chenchen Wang 2016 | CE VS NEI | 0.34645486 | -0.1591648 | 0.8520746 | 1 |
| Chenchen Wang 2016 | TC VS NEI | 0.34655628 | -0.1023427 | 0.7954553 | 1 |
| Chenchen Wang 2016 | WQX VS NEI | -0.08164796 | -0.5797738 | 0.4164779 | 1 |
| Chenchen Wang 2016 | YG VS NEI | 0.43623154 | -0.4923812 | 1.364844 | 1 |
| Michael Wortley 2013 | CE VS NEI | 0.28919038 | -0.1871791 | 0.7655599 | 1 |
| Michael Wortley 2013 | TC VS NEI | 0.26904096 | -0.1191605 | 0.6572424 | 1 |
| Michael Wortley 2013 | WQX VS NEI | -0.08161516 | -0.5739746 | 0.4107443 | 1 |
| Michael Wortley 2013 | YG VS NEI | 0.40617232 | -0.5136566 | 1.326001 | 1 |
| Augustine C. Lee 2017 | CE VS NEI | 0.27919978 | -0.2373407 | 0.7957403 | 1 |
| Augustine C. Lee 2017 | TC VS NEI | 0.25456817 | -0.1936931 | 0.7028294 | 1 |
| Augustine C. Lee 2017 | WQX VS NEI | -0.08180114 | -0.6097955 | 0.4461932 | 1 |
| Augustine C. Lee 2017 | YG VS NEI | 0.40113681 | -0.5533614 | 1.355635 | 1 |
| Chunmei Xiao 2020 | CE VS NEI | 0.27793442 | -0.2107771 | 0.7666459 | 1 |
| Chunmei Xiao 2020 | TC VS NEI | 0.25281825 | -0.1327332 | 0.6383697 | 1 |
| Chunmei Xiao 2020 | WQX VS NEI | -0.11401703 | -0.8776285 | 0.6495944 | 1 |
| Chunmei Xiao 2020 | YG VS NEI | 0.40048086 | -0.5508493 | 1.351811 | 1 |
| Alexander B. Kuntz 2018 | CE VS NEI | 0.23798115 | -0.2829648 | 0.758927 | 1 |
| Alexander B. Kuntz 2018 | TC VS NEI | 0.23151463 | -0.1448012 | 0.6078304 | 1 |
| Alexander B. Kuntz 2018 | WQX VS NEI | -0.0816086 | -0.5728389 | 0.4096217 | 1 |
| Chun Mei Xiao 2020 | CE VS NEI | 0.27793442 | -0.2107771 | 0.7666459 | 1 |
| Chun Mei Xiao 2020 | TC VS NEI | 0.25281827 | -0.1327332 | 0.6383697 | 1 |
| Chun Mei Xiao 2020 | WQX VS NEI | -0.05207492 | -0.7857032 | 0.6815534 | 1 |
| Chun Mei Xiao 2020 | YG VS NEI | 0.40048088 | -0.5508493 | 1.351811 | 1 |
| Guo-Xin Ni 2010 | CE VS NEI | 0.37747479 | -0.0356447 | 0.7905943 | 1 |
| Guo-Xin Ni 2010 | TC VS NEI | 0.18492977 | -0.1040793 | 0.4739388 | 1 |
| Guo-Xin Ni 2010 | WQX VS NEI | -0.08050376 | -0.4490468 | 0.2880392 | 1 |
| Guo-Xin Ni 2010 | YG VS NEI | 0.45230742 | -0.367554 | 1.272169 | 1 |
| CHENCHEN WANG 2009 | CE VS NEI | 0.37326282 | -0.0557905 | 0.8023162 | 1 |
| CHENCHEN WANG 2009 | TC VS NEI | 0.19567559 | -0.1109947 | 0.5023459 | 1 |
| CHENCHEN WANG 2009 | WQX VS NEI | -0.08082552 | -0.4754781 | 0.3138271 | 1 |
| CHENCHEN WANG 2009 | YG VS NEI | 0.45011756 | -0.389778 | 1.290013 | 1 |
| Hwa-Jin Lee 2009 | CE VS NEI | 0.03813984 | -0.3731663 | 0.449446 | 1 |
| Hwa-Jin Lee 2009 | TC VS NEI | 0.21302005 | -0.0894267 | 0.5154667 | 1 |
| Hwa-Jin Lee 2009 | WQX VS NEI | -0.0798558 | -0.4082101 | 0.2484985 | 1 |
| Hwa-Jin Lee 2009 | YG VS NEI | 0.27126482 | -0.5232183 | 1.065748 | 1 |
| Mona Nahayatbin 2018 | CE VS NEI | 0.14739349 | -0.4088729 | 0.7036599 | 1 |
| Mona Nahayatbin 2018 | TC VS NEI | 0.17710765 | -0.2307944 | 0.5850097 | 1 |
| Mona Nahayatbin 2018 | WQX VS NEI | -0.08169348 | -0.5881662 | 0.4247792 | 1 |
| Mona Nahayatbin 2018 | YG VS NEI | 0.33194995 | -0.6113502 | 1.27525 | 1 |

**Meta-regression**

**Countries Conducting Research**

**Table S21** Meta-Regression Analysis of TUG（Timed-up-and-go）Using Countries Conducting Research as a Moderator.

|  |  | **Coefficient** | **Std. err.** | **z** | **P>z** | **[95% conf.** | **interval]** |
| --- | --- | --- | --- | --- | --- | --- | --- |
| NEI vs CE | country | 0.25246 | 2.592925 | 0.1 | 0.922 | -4.829579 | 5.334499 |
| NEI vs CE | constant term | -0.4147923 | 15.49893 | -0.03 | 0.979 | -30.79214 | 29.96255 |
| TC vs CE | country | 0.589505 | 2.745623 | 0.21 | 0.83 | -4.791818 | 5.970828 |
| TC vs CE | constant term | -1.251769 | 15.60041 | -0.08 | 0.936 | -31.828 | 29.32446 |
| WQX vs CE | constant term | -0.0792707 | 10.33633 | -0.01 | 0.994 | -20.3381 | 20.17955 |
| YG vs CE | constant term | -0.4000037 | 0.5033522 | -0.79 | 0.427 | -1.386556 | 0.5865485 |

**Table S22** Meta-Regression Analysis of Mental health Using Countries Conducting Research as a Moderator.

|  |  | **Coefficient** | **Std. err.** | **z** | **P>z** | **[95% conf.** | **interval]** |
| --- | --- | --- | --- | --- | --- | --- | --- |
| CE VS BDJ | country | -0.0423425 | 4.936369 | -0.01 | 0.993 | -9.717447 | 9.632762 |
| CE VS BDJ | constant term | -0.3833902 | 9.885183 | -0.04 | 0.969 | -19.75799 | 18.99121 |
| NEI VS BDJ | country | 0.0045616 | 4.939597 | 0 | 0.999 | -9.67687 | 9.685994 |
| NEI VS BDJ | constant term | -0.6535764 | 9.890411 | -0.07 | 0.947 | -20.03842 | 18.73127 |
| TC VS BDJ | country | 0.0578943 | 4.936207 | 0.01 | 0.991 | -9.616893 | 9.732681 |
| TC VS BDJ | constant term | -0.5104672 | 9.883414 | -0.05 | 0.959 | -19.8816 | 18.86067 |
| YG VS BDJ | country | -0.0599796 | 4.939744 | -0.01 | 0.99 | -9.741701 | 9.621741 |
| YG VS BDJ | constant term | -0.0496396 | 9.892571 | -0.01 | 0.996 | -19.43872 | 19.33944 |
| YJJ VS BDJ | constant term | 1.323959 | 0.6517471 | 2.03 | 0.042 | 0.0465584 | 2.60136 |

**Table S23** Meta-Regression Analysis of WOMAC-Pain Using Countries Conducting Research as a Moderator.

|  |  | **Coefficient** | **Std. err.** | **z** | **P>z** | **[95% conf.** | **interval]** |
| --- | --- | --- | --- | --- | --- | --- | --- |
| CE VS BDJ | country | -0.0478407 | 4.227542 | -0.01 | 0.991 | -8.333671 | 8.23799 |
| CE VS BDJ | constant term | 0.7407391 | 8.465144 | 0.09 | 0.93 | -15.85064 | 17.33212 |
| NEI VS BDJ | country | -0.0114176 | 4.228326 | 0 | 0.998 | -8.298784 | 8.275948 |
| NEI VS BDJ | constant term | 0.206849 | 8.461623 | 0.02 | 0.98 | -16.37763 | 16.79133 |
| PIL VS BDJ | country | -1.399707 | 4.303123 | -0.33 | 0.745 | -9.833673 | 7.034258 |
| PIL VS BDJ | constant term | 4.100011 | 8.874609 | 0.46 | 0.644 | -13.2939 | 21.49393 |
| TC VS BDJ | country | -0.0282521 | 4.227518 | -0.01 | 0.995 | -8.314036 | 8.257532 |
| TC VS BDJ | constant term | -0.2001743 | 8.461116 | -0.02 | 0.981 | -16.78366 | 16.38331 |
| WQX VS BDJ | constant term | -0.3028359 | 0.3123127 | -0.97 | 0.332 | -0.9149575 | 0.3092856 |
| YG VS BDJ | country | 0.1761337 | 4.228231 | 0.04 | 0.967 | -8.111047 | 8.463315 |
| YG VS BDJ | constant term | -0.8422989 | 8.468923 | -0.1 | 0.921 | -17.44108 | 15.75648 |
| YJJ VS BDJ | constant term | 0.160939 | 0.5275106 | 0.31 | 0.76 | -0.8729627 | 1.194841 |

**Table S24** Meta-Regression Analysis of WOMAC-Physical function Using Countries Conducting Research as a Moderator.

|  |  | **Coefficient** | **Std. err.** | **z** | **P>z** | **[95% conf.** | **interval]** |
| --- | --- | --- | --- | --- | --- | --- | --- |
| CE VS BDJ | country | -0.1019758 | 3.923008 | -0.03 | 0.979 | -7.79093 | 7.586978 |
| CE VS BDJ | constant term | 1.331132 | 7.855696 | 0.17 | 0.865 | -14.06575 | 16.72801 |
| NEI VS BDJ | country | -0.0869012 | 3.922982 | -0.02 | 0.982 | -7.775805 | 7.602003 |
| NEI VS BDJ | constant term | 1.097049 | 7.851205 | 0.14 | 0.889 | -14.29103 | 16.48513 |
| PIL VS BDJ | country | -1.359844 | 4.003538 | -0.34 | 0.734 | -9.206634 | 6.486946 |
| PIL VS BDJ | constant term | 4.126299 | 8.284325 | 0.5 | 0.618 | -12.11068 | 20.36328 |
| TC VS BDJ | country | -0.0910581 | 3.922834 | -0.02 | 0.981 | -7.779672 | 7.597555 |
| TC VS BDJ | constant term | 0.5772504 | 7.852228 | 0.07 | 0.941 | -14.81283 | 15.96734 |
| WQX VS BDJ | constant term | 0.7751375 | 0.3219572 | 2.41 | 0.016 | 0.1441129 | 1.406162 |
| YG VS BDJ | country | 0.0536649 | 3.923446 | 0.01 | 0.989 | -7.636148 | 7.743478 |
| YG VS BDJ | constant term | 0.1302413 | 7.858651 | 0.02 | 0.987 | -15.27243 | 15.53291 |
| YJJ VS BDJ | constant term | 1.06371 | 0.4851731 | 2.19 | 0.028 | 0.1127884 | 2.014632 |

**Table S25** Meta-Regression Analysis of WOMAC-Stiffness Using Countries Conducting Research as a Moderator.

|  |  | **Coefficient** | **Std. err.** | **z** | **P>z** | **[95% conf.** | **interval]** |
| --- | --- | --- | --- | --- | --- | --- | --- |
| CE VS BDJ | country | -0.2452007 | 4.303276 | -0.06 | 0.955 | -8.679466 | 8.189064 |
| CE VS BDJ | constant term | 2.701852 | 8.615744 | 0.31 | 0.754 | -14.1847 | 19.5884 |
| NEI VS BDJ | country | -0.1576173 | 4.303879 | -0.04 | 0.971 | -8.593066 | 8.277831 |
| NEI VS BDJ | constant term | 1.986197 | 8.612955 | 0.23 | 0.818 | -14.89488 | 18.86728 |
| PIL VS BDJ | constant term | 0.3973679 | 8.628413 | 0.05 | 0.963 | -16.51401 | 17.30875 |
| TC VS BDJ | country | -0.2119852 | 4.303277 | -0.05 | 0.961 | -8.646253 | 8.222283 |
| TC VS BDJ | constant term | 1.910914 | 8.612727 | 0.22 | 0.824 | -14.96972 | 18.79155 |
| WQX VS BDJ | constant term | 1.378984 | 0.2960737 | 4.66 | 0 | 0.7986898 | 1.959277 |
| YG VS BDJ | country | 0.0287168 | 4.303831 | 0.01 | 0.995 | -8.406638 | 8.464071 |
| YG VS BDJ | constant term | 0.8832467 | 8.618865 | 0.1 | 0.918 | -16.00942 | 17.77591 |
| YJJ VS BDJ | constant term | 2.075177 | 0.4917153 | 4.22 | 0 | 1.111432 | 3.038921 |

**Table S26** Meta-Regression Analysis of 6MWT(6-min walk test) Using Countries Conducting Research as a Moderator.

|  |  | **Coefficient** | **Std. err.** | **z** | **P>z** | **[95% conf.** | **interval]** |
| --- | --- | --- | --- | --- | --- | --- | --- |
| NEI VS CE | country | -0.1786606 | 0.0617042 | -2.9 | 0.004 | -0.2995985 | -0.0577226 |
| NEI VS CE | constant term | 0.6250896 | 0.3231905 | 1.93 | 0.053 | -0.0083522 | 1.258531 |
| TC VS CE | country | -0.1504587 | 0.0553556 | -2.72 | 0.007 | -0.2589537 | -0.0419637 |
| TC VS CE | constant term | 0.7082061 | 0.2946349 | 2.4 | 0.016 | 0.1307323 | 1.28568 |
| WQX VS CE | constant term | 0.188139 | 0.2850576 | 0.66 | 0.509 | -0.3705637 | 0.7468417 |
| YG VS CE | constant term | 0.0424075 | 0.3989653 | 0.11 | 0.915 | -0.7395501 | 0.8243652 |

**follow-up time**

**Table S27** Meta-Regression Analysis of TUG（Timed-up-and-go）Using follow-up time as a Moderator.

|  |  | **Coefficient** | **Std. err.** | **z** | **P>z** | **[95% conf.** | **interval]** |
| --- | --- | --- | --- | --- | --- | --- | --- |
| NEI vs CE | follow-up time | -0.0553098 | 0.6203228 | -0.09 | 0.929 | -1.27112 | 1.1605 |
| NEI vs CE | constant term | 1.762351 | 7.585351 | 0.23 | 0.816 | -13.10466 | 16.62937 |
| TC vs CE | follow-up time | -0.0632976 | 0.6205503 | -0.1 | 0.919 | -1.279554 | 1.152959 |
| TC vs CE | constant term | 1.810967 | 7.603307 | 0.24 | 0.812 | -13.09124 | 16.71318 |
| WQX vs CE | follow-up time | 0.0780237 | 0.6244308 | 0.12 | 0.901 | -1.145838 | 1.301885 |
| WQX vs CE | constant term | -0.9376709 | 7.717751 | -0.12 | 0.903 | -16.06419 | 14.18884 |
| YG vs CE | constant term | -0.4001574 | 0.503336 | -0.8 | 0.427 | -1.386678 | 0.5863629 |

**Table S28** Meta-Regression Analysis of Mental health Using follow-up time as a Moderator.

|  |  | **Coefficient** | **Std. err.** | **z** | **P>z** | **[95% conf.** | **interval]** |
| --- | --- | --- | --- | --- | --- | --- | --- |
| CE VS BDJ | follow-up time | 0.0255936 | 0.8097036 | 0.03 | 0.975 | -1.561396 | 1.612584 |
| CE VS BDJ | constant term | -0.6549808 | 6.520532 | -0.1 | 0.92 | -13.43499 | 12.12503 |
| NEI VS BDJ | follow-up time | 0.0310916 | 0.8093946 | 0.04 | 0.969 | -1.555293 | 1.617476 |
| NEI VS BDJ | constant term | -0.8937325 | 6.505335 | -0.14 | 0.891 | -13.64395 | 11.85649 |
| TC VS BDJ | follow-up time | 0.0061905 | 0.8094839 | 0.01 | 0.994 | -1.580369 | 1.59275 |
| TC VS BDJ | constant term | -0.1622143 | 6.512564 | -0.02 | 0.98 | -12.92661 | 12.60218 |
| YG VS BDJ | follow-up time | -0.0311228 | 0.8120424 | -0.04 | 0.969 | -1.622697 | 1.560451 |
| YG VS BDJ | constant term | 0.13407 | 6.543598 | 0.02 | 0.984 | -12.69115 | 12.95929 |
| YJJ VS BDJ | constant term | 1.444169 | 3.281489 | 0.44 | 0.66 | -4.987432 | 7.87577 |

**Table S29** Meta-Regression Analysis of WOMAC-Pain Using follow-up time as a Moderator.

|  |  | **Coefficient** | **Std. err.** | **z** | **P>z** | **[95% conf.** | **interval]** |
| --- | --- | --- | --- | --- | --- | --- | --- |
| CE VS BDJ | follow-up time | -0.1994352 | 0.1437581 | -1.39 | 0.165 | -0.481196 | 0.0823255 |
| CE VS BDJ | constant term | 2.623005 | 1.699297 | 1.54 | 0.123 | -0.7075563 | 5.953566 |
| NEI VS BDJ | follow-up time | -0.2297384 | 0.1409424 | -1.63 | 0.103 | -0.5059805 | 0.0465036 |
| NEI VS BDJ | constant term | 2.754565 | 1.600643 | 1.72 | 0.085 | -0.3826384 | 5.891768 |
| PIL VS BDJ | follow-up time | 1.152551 | 0.7961099 | 1.45 | 0.148 | -0.4077953 | 2.712898 |
| PIL VS BDJ | constant term | -8.889247 | 6.307075 | -1.41 | 0.159 | -21.25089 | 3.472393 |
| TC VS BDJ | follow-up time | -0.2419797 | 0.1425015 | -1.7 | 0.089 | -0.5212774 | 0.0373181 |
| TC VS BDJ | constant term | 2.55466 | 1.655734 | 1.54 | 0.123 | -0.6905184 | 5.799838 |
| WQX VS BDJ | follow-up time | -0.2329504 | 0.1446074 | -1.61 | 0.107 | -0.5163758 | 0.050475 |
| WQX VS BDJ | constant term | 2.328295 | 1.744426 | 1.33 | 0.182 | -1.090717 | 5.747308 |
| YG VS BDJ | follow-up time | -0.1651072 | 0.1430578 | -1.15 | 0.248 | -0.4454952 | 0.1152809 |
| YG VS BDJ | constant term | 1.205812 | 1.674884 | 0.72 | 0.472 | -2.076901 | 4.488524 |
| YJJ VS BDJ | constant term | -0.2543017 | 0.5324801 | -0.48 | 0.633 | -1.297943 | 0.7893401 |

**Table S30** Meta-Regression Analysis of WOMAC-Physical function Using follow-up time as a Moderator.

|  |  | **Coefficient** | **Std. err.** | **z** | **P>z** | **[95% conf.** | **interval]** |
| --- | --- | --- | --- | --- | --- | --- | --- |
| CE VS BDJ | follow-up time | 0.054845 | 0.1425122 | 0.38 | 0.7 | -0.2244737 | 0.3341637 |
| CE VS BDJ | constant term | 0.4012056 | 1.671454 | 0.24 | 0.81 | -2.874783 | 3.677195 |
| NEI VS BDJ | follow-up time | 0.0443259 | 0.1397922 | 0.32 | 0.751 | -0.2296618 | 0.3183137 |
| NEI VS BDJ | constant term | 0.4276724 | 1.580009 | 0.27 | 0.787 | -2.669088 | 3.524433 |
| PIL VS BDJ | follow-up time | 1.312697 | 0.8170845 | 1.61 | 0.108 | -0.2887594 | 2.914153 |
| PIL VS BDJ | constant term | -10.64003 | 6.466317 | -1.65 | 0.1 | -23.31378 | 2.033718 |
| TC VS BDJ | follow-up time | 0.0316588 | 0.1412184 | 0.22 | 0.823 | -0.2451241 | 0.3084418 |
| TC VS BDJ | constant term | 0.1173646 | 1.621371 | 0.07 | 0.942 | -3.060464 | 3.295193 |
| WQX VS BDJ | follow-up time | 0.0775897 | 0.1446906 | 0.54 | 0.592 | -0.2059988 | 0.3611781 |
| WQX VS BDJ | constant term | -0.3315633 | 1.737082 | -0.19 | 0.849 | -3.736182 | 3.073055 |
| YG VS BDJ | follow-up time | 0.0856987 | 0.1418493 | 0.6 | 0.546 | -0.1923209 | 0.3637182 |
| YG VS BDJ | constant term | -0.7986286 | 1.651284 | -0.48 | 0.629 | -4.035086 | 2.437829 |
| YJJ VS BDJ | constant term | 0.9958815 | 0.5224357 | 1.91 | 0.057 | -0.0280737 | 2.019837 |

**Table S31** Meta-Regression Analysis of WOMAC-Stiffness Using follow-up time as a Moderator.

|  |  | **Coefficient** | **Std. err.** | **z** | **P>z** | **[95% conf.** | **interval]** |
| --- | --- | --- | --- | --- | --- | --- | --- |
| CE VS BDJ | follow-up time | 0.3109961 | 0.1391618 | 2.23 | 0.025 | 0.038244 | 0.5837482 |
| CE VS BDJ | constant term | -1.174966 | 1.645985 | -0.71 | 0.475 | -4.401036 | 2.051105 |
| NEI VS BDJ | follow-up time | 0.317828 | 0.1360597 | 2.34 | 0.019 | 0.051156 | 0.5845001 |
| NEI VS BDJ | constant term | -1.834213 | 1.522415 | -1.2 | 0.228 | -4.818092 | 1.149667 |
| PIL VS BDJ | constant term | -0.3215512 | 0.9409725 | -0.34 | 0.733 | -2.165823 | 1.522721 |
| TC VS BDJ | follow-up time | 0.2979699 | 0.1388191 | 2.15 | 0.032 | 0.0258896 | 0.5700503 |
| TC VS BDJ | constant term | -1.571805 | 1.64274 | -0.96 | 0.339 | -4.791516 | 1.647906 |
| WQX VS BDJ | follow-up time | 0.3088226 | 0.1390111 | 2.22 | 0.026 | 0.0363658 | 0.5812794 |
| WQX VS BDJ | constant term | -1.948961 | 1.643902 | -1.19 | 0.236 | -5.17095 | 1.273027 |
| YG VS BDJ | follow-up time | 0.3711868 | 0.1379638 | 2.69 | 0.007 | 0.1007828 | 0.6415909 |
| YG VS BDJ | constant term | -3.189338 | 1.594718 | -2 | 0.046 | -6.314928 | -0.0637482 |
| YJJ VS BDJ | constant term | 2.420708 | 0.5382867 | 4.5 | 0 | 1.365686 | 3.475731 |

**Table S32** Meta-Regression Analysis of 6MWT(6-min walk test) Using follow-up time as a Moderator.

|  |  | **Coefficient** | **Std. err.** | **z** | **P>z** | **[95% conf.** | **interval]** |
| --- | --- | --- | --- | --- | --- | --- | --- |
| NEI VS CE | follow-up time | 0.0625469 | 0.020982 | 2.98 | 0.003 | 0.0214229 | 0.1036709 |
| NEI VS CE | constant term | -1.094899 | 0.3501253 | -3.13 | 0.002 | -1.781133 | -0.4086665 |
| TC VS CE | follow-up time | 0.0506693 | 0.0182024 | 2.78 | 0.005 | 0.0149934 | 0.0863453 |
| TC VS CE | constant term | -0.7390121 | 0.3129745 | -2.36 | 0.018 | -1.352431 | -0.1255934 |
| WQX VS CE | follow-up time | 0.0677056 | 0.0343143 | 1.97 | 0.048 | 0.0004508 | 0.1349604 |
| WQX VS CE | constant term | -1.270787 | 0.6368052 | -2 | 0.046 | -2.518902 | -0.0226717 |
| YG VS CE | constant term | 0.0901942 | 0.3950688 | 0.23 | 0.819 | -0.6841265 | 0.8645149 |

**Mean age**

**Table S33** Meta-Regression Analysis of TUG（Timed-up-and-go）Using Mean age as a Moderator.

|  |  | **Coefficient** | **Std. err.** | **z** | **P>z** | **[95% conf.** | **interval]** |
| --- | --- | --- | --- | --- | --- | --- | --- |
| NEI vs CE | mean age | 0.3100613 | 3.615346 | 0.09 | 0.932 | -6.775888 | 7.39601 |
| NEI vs CE | constant term | -19.67578 | 242.1492 | -0.08 | 0.935 | -494.2795 | 454.9279 |
| TC vs CE | mean age | 0.2426022 | 3.619684 | 0.07 | 0.947 | -6.851848 | 7.337052 |
| TC vs CE | constant term | -15.52129 | 242.4138 | -0.06 | 0.949 | -490.6437 | 459.6011 |
| WQX vs CE | constant term | 1.859056 | 10.97663 | 0.17 | 0.866 | -19.65474 | 23.37286 |
| YG vs CE | constant term | -0.4001923 | 0.503336 | -0.8 | 0.427 | -1.386713 | 0.5863282 |

**Table S34** Meta-Regression Analysis of Mental health Using Mean age as a Moderator.

|  |  | **Coefficient** | **Std. err.** | **z** | **P>z** | **[95% conf.** | **interval]** |
| --- | --- | --- | --- | --- | --- | --- | --- |
| CE VS BDJ | mean age | 0.0238802 | 1.435368 | 0.02 | 0.987 | -2.789389 | 2.837149 |
| CE VS BDJ | constant term | -1.996702 | 93.30321 | -0.02 | 0.983 | -184.8676 | 180.8742 |
| NEI VS BDJ | mean age | -0.0319559 | 1.435647 | -0.02 | 0.982 | -2.845773 | 2.781861 |
| NEI VS BDJ | constant term | 1.432496 | 93.31878 | 0.02 | 0.988 | -181.4689 | 184.3339 |
| TC VS BDJ | mean age | 0.0209785 | 1.435332 | 0.01 | 0.988 | -2.792221 | 2.834178 |
| TC VS BDJ | constant term | -1.595791 | 93.29806 | -0.02 | 0.986 | -184.4566 | 181.265 |
| YG VS BDJ | mean age | 0.0105096 | 1.435588 | 0.01 | 0.994 | -2.803191 | 2.82421 |
| YG VS BDJ | constant term | -0.891998 | 93.3155 | -0.01 | 0.992 | -183.787 | 182.003 |
| YJJ VS BDJ | constant term | 1.10876 | 14.36999 | 0.08 | 0.938 | -27.05591 | 29.27343 |

**Table S35** Meta-Regression Analysis of WOMAC-Pain Using Mean age as a Moderator.

|  |  | **Coefficient** | **Std. err.** | **z** | **P>z** | **[95% conf.** | **interval]** |
| --- | --- | --- | --- | --- | --- | --- | --- |
| CE VS BDJ | mean age | 0.7444396 | 0.511642 | 1.46 | 0.146 | -0.2583604 | 1.74724 |
| CE VS BDJ | constant term | -47.0541 | 32.85031 | -1.43 | 0.152 | -111.4395 | 17.33131 |
| NEI VS BDJ | mean age | 0.7790137 | 0.5106878 | 1.53 | 0.127 | -0.2219161 | 1.779943 |
| NEI VS BDJ | constant term | -49.83602 | 32.78154 | -1.52 | 0.128 | -114.0867 | 14.41462 |
| PIL VS BDJ | mean age | 0.5753848 | 0.5207214 | 1.1 | 0.269 | -0.4452104 | 1.59598 |
| PIL VS BDJ | constant term | -38.11391 | 33.34467 | -1.14 | 0.253 | -103.4683 | 27.24044 |
| TC VS BDJ | mean age | 0.7782789 | 0.5110077 | 1.52 | 0.128 | -0.2232778 | 1.779836 |
| TC VS BDJ | constant term | -50.24601 | 32.80197 | -1.53 | 0.126 | -114.5367 | 14.04468 |
| WQX VS BDJ | constant term | 4.199327 | 2.97877 | 1.41 | 0.159 | -1.638955 | 10.03761 |
| YG VS BDJ | mean age | 0.6787736 | 0.5118005 | 1.33 | 0.185 | -0.324337 | 1.681884 |
| YG VS BDJ | constant term | -43.57107 | 32.85798 | -1.33 | 0.185 | -107.9715 | 20.82939 |
| YJJ VS BDJ | constant term | -6.593557 | 4.733484 | -1.39 | 0.164 | -15.87102 | 2.683901 |

**Table S36** Meta-Regression Analysis of WOMAC-Physical function Using Mean age as a Moderator.

|  |  | **Coefficient** | **Std. err.** | **z** | **P>z** | **[95% conf.** | **interval]** |
| --- | --- | --- | --- | --- | --- | --- | --- |
| CE VS BDJ | mean age | -0.1905336 | 0.4977859 | -0.38 | 0.702 | -1.166176 | 0.7851087 |
| CE VS BDJ | constant term | 13.36426 | 31.96111 | 0.42 | 0.676 | -49.27837 | 76.00689 |
| NEI VS BDJ | mean age | -0.156664 | 0.4965672 | -0.32 | 0.752 | -1.129918 | 0.8165899 |
| NEI VS BDJ | constant term | 10.98271 | 31.87821 | 0.34 | 0.73 | -51.49745 | 73.46286 |
| PIL VS BDJ | mean age | -0.3477562 | 0.5072834 | -0.69 | 0.493 | -1.342013 | 0.6465011 |
| PIL VS BDJ | constant term | 21.34741 | 32.4759 | 0.66 | 0.511 | -42.30418 | 84.999 |
| TC VS BDJ | mean age | -0.1831132 | 0.4973903 | -0.37 | 0.713 | -1.15798 | 0.7917539 |
| TC VS BDJ | constant term | 12.12448 | 31.92987 | 0.38 | 0.704 | -50.45691 | 74.70587 |
| WQX VS BDJ | constant term | -0.1291381 | 2.897236 | -0.04 | 0.964 | -5.807616 | 5.54934 |
| YG VS BDJ | mean age | -0.2332027 | 0.4976943 | -0.47 | 0.639 | -1.208666 | 0.7422602 |
| YG VS BDJ | constant term | 15.54376 | 31.95373 | 0.49 | 0.627 | -47.08441 | 78.17192 |
| YJJ VS BDJ | constant term | 2.821341 | 4.604024 | 0.61 | 0.54 | -6.202381 | 11.84506 |

**Table S37** Meta-Regression Analysis of WOMAC-Stiffness Using Mean age as a Moderator.

|  |  | **Coefficient** | **Std. err.** | **z** | **P>z** | **[95% conf.** | **interval]** |
| --- | --- | --- | --- | --- | --- | --- | --- |
| CE VS BDJ | mean age | -1.099081 | 0.4907896 | -2.24 | 0.025 | -2.061011 | -0.1371512 |
| CE VS BDJ | constant term | 72.59631 | 31.53116 | 2.3 | 0.021 | 10.79637 | 134.3963 |
| NEI VS BDJ | mean age | -1.148824 | 0.4896111 | -2.35 | 0.019 | -2.108444 | -0.1892042 |
| NEI VS BDJ | constant term | 75.4815 | 31.45057 | 2.4 | 0.016 | 13.83952 | 137.1235 |
| PIL VS BDJ | constant term | -0.1675384 | 0.7394225 | -0.23 | 0.821 | -1.61678 | 1.281703 |
| TC VS BDJ | mean age | -1.141283 | 0.490482 | -2.33 | 0.02 | -2.10261 | -0.1799554 |
| TC VS BDJ | constant term | 74.84053 | 31.50757 | 2.38 | 0.018 | 13.08683 | 136.5942 |
| WQX VS BDJ | constant term | -5.25921 | 2.837367 | -1.85 | 0.064 | -10.82035 | 0.301927 |
| YG VS BDJ | mean age | -1.223996 | 0.4906831 | -2.49 | 0.013 | -2.185717 | -0.262275 |
| YG VS BDJ | constant term | 79.99512 | 31.52158 | 2.54 | 0.011 | 18.21396 | 141.7763 |
| YJJ VS BDJ | constant term | 12.01005 | 4.55755 | 2.64 | 0.008 | 3.077417 | 20.94269 |

**Table S38** Meta-Regression Analysis of 6MWT(6-min walk test) Using Mean age as a Moderator.

|  |  | **Coefficient** | **Std. err.** | **z** | **P>z** | **[95% conf.** | **interval]** |
| --- | --- | --- | --- | --- | --- | --- | --- |
| NEI VS CE | mean age | 0.0011868 | 0.0533662 | 0.02 | 0.982 | -0.1034091 | 0.1057826 |
| NEI VS CE | constant term | -0.2878582 | 3.322647 | -0.09 | 0.931 | -6.800127 | 6.224411 |
| TC VS CE | mean age | -0.030068 | 0.0455605 | -0.66 | 0.509 | -0.1193648 | 0.0592289 |
| TC VS CE | constant term | 1.877542 | 2.898911 | 0.65 | 0.517 | -3.804219 | 7.559302 |
| WQX VS CE | constant term | -0.2865203 | 0.5637404 | -0.51 | 0.611 | -1.391431 | 0.8183906 |
| YG VS CE | constant term | 0.1556714 | 0.496322 | 0.31 | 0.754 | -0.8171018 | 1.128445 |

**Network GRADE Classification**

**Table S39** Grade Grading Evaluation Form of TUG(Timed-up-and-go)

| **Comparison** | **Number of studies** | **Within-study bias** | **Reporting bias** | **Indirectness** | **Imprecision** | **Heterogeneity** | **Incoherence** | **Confidence rating** | **Reason(s) for downgrading** |
| --- | --- | --- | --- | --- | --- | --- | --- | --- | --- |
| CE:NEI | 1 | No concerns | Low risk | No concerns | Some concerns | Some concerns | Some concerns | Very low | ["Imprecision","Heterogeneity","Incoherence"] |
| CE:YG | 1 | No concerns | Low risk | No concerns | Some concerns | No concerns | Some concerns | Low | ["Imprecision","Incoherence"] |
| NEI:TC | 3 | No concerns | Low risk | No concerns | Some concerns | Some concerns | Some concerns | Very low | ["Imprecision","Heterogeneity","Incoherence"] |
| NEI:WQX | 2 | No concerns | Low risk | No concerns | No concerns | Some concerns | Some concerns | Low | ["Heterogeneity","Incoherence"] |
| NEI:YG | 1 | No concerns | Low risk | No concerns | Some concerns | Some concerns | Some concerns | Very low | ["Imprecision","Heterogeneity","Incoherence"] |
| CE:TC | 0 | No concerns | Low risk | No concerns | Some concerns | No concerns | Some concerns | Low | ["Imprecision","Incoherence"] |
| CE:WQX | 0 | No concerns | Low risk | No concerns | Some concerns | Some concerns | Some concerns | Very low | ["Imprecision","Heterogeneity","Incoherence"] |
| TC:WQX | 0 | No concerns | Low risk | No concerns | Some concerns | Some concerns | Some concerns | Very low | ["Imprecision","Heterogeneity","Incoherence"] |
| TC:YG | 0 | No concerns | Low risk | No concerns | Some concerns | No concerns | Some concerns | Low | ["Imprecision","Incoherence"] |
| WQX:YG | 0 | No concerns | Low risk | No concerns | Some concerns | Some concerns | Some concerns | Very low | ["Imprecision","Heterogeneity","Incoherence"] |

**Table S40** Grade Grading Evaluation Form of Mental health

| **Comparison** | **Number of studies** | **Within-study bias** | **Reporting bias** | **Indirectness** | **Imprecision** | **Heterogeneity** | **Incoherence** | **Confidence rating** | **Reason(s) for downgrading** |
| --- | --- | --- | --- | --- | --- | --- | --- | --- | --- |
| BDJ:NEI | 1 | Some concerns | Low risk | No concerns | Some concerns | No concerns | No concerns | Low | ["Within-study bias","Imprecision"] |
| CE:TC | 3 | Some concerns | Low risk | No concerns | No concerns | Some concerns | No concerns | Low | ["Within-study bias","Heterogeneity"] |
| CE:YG | 1 | Some concerns | Low risk | No concerns | Some concerns | No concerns | No concerns | Low | ["Within-study bias","Imprecision"] |
| CE:YJJ | 1 | No concerns | Low risk | No concerns | No concerns | No concerns | No concerns | High | [] |
| NEI:TC | 4 | No concerns | Low risk | No concerns | No concerns | No concerns | No concerns | High | [] |
| NEI:YG | 3 | No concerns | Low risk | No concerns | No concerns | No concerns | No concerns | High | [] |
| BDJ:CE | 0 | Some concerns | Low risk | No concerns | Some concerns | No concerns | No concerns | Low | ["Within-study bias","Imprecision"] |
| BDJ:TC | 0 | Some concerns | Low risk | No concerns | Some concerns | Some concerns | No concerns | Very low | ["Within-study bias","Imprecision","Heterogeneity"] |
| BDJ:YG | 0 | Some concerns | Low risk | No concerns | Major concerns | No concerns | No concerns | Very low | ["Within-study bias","Imprecision"] |
| BDJ:YJJ | 0 | Some concerns | Low risk | No concerns | No concerns | Some concerns | No concerns | Low | ["Within-study bias","Heterogeneity"] |
| CE:NEI | 0 | Some concerns | Low risk | No concerns | No concerns | No concerns | No concerns | Moderate | ["Within-study bias"] |
| NEI:YJJ | 0 | No concerns | Low risk | No concerns | No concerns | No concerns | No concerns | High | [] |
| TC:YG | 0 | No concerns | Low risk | No concerns | No concerns | Some concerns | No concerns | Moderate | ["Heterogeneity"] |
| TC:YJJ | 0 | No concerns | Low risk | No concerns | No concerns | No concerns | No concerns | High | [] |
| YG:YJJ | 0 | No concerns | Low risk | No concerns | No concerns | No concerns | No concerns | High | [] |

**Table S41** Grade Grading Evaluation Form of WOMAC-Pain

| **Comparison** | **Number of studies** | **Within-study bias** | **Reporting bias** | **Indirectness** | **Imprecision** | **Heterogeneity** | **Incoherence** | **Confidence rating** | **Reason(s) for downgrading** |
| --- | --- | --- | --- | --- | --- | --- | --- | --- | --- |
| BDJ:NEI | 3 | No concerns | Low risk | No concerns | Some concerns | Some concerns | Some concerns | Very low | ["Imprecision","Heterogeneity","Incoherence"] |
| CE:PIL | 2 | Some concerns | Low risk | No concerns | No concerns | No concerns | Some concerns | Low | ["Within-study bias","Incoherence"] |
| CE:TC | 4 | Some concerns | Low risk | No concerns | No concerns | No concerns | No concerns | Moderate | ["Within-study bias"] |
| CE:YG | 2 | Some concerns | Low risk | No concerns | No concerns | Some concerns | No concerns | Low | ["Within-study bias","Heterogeneity"] |
| CE:YJJ | 1 | No concerns | Low risk | No concerns | Some concerns | Some concerns | Some concerns | Very low | ["Imprecision","Heterogeneity","Incoherence"] |
| NEI:TC | 7 | No concerns | Low risk | No concerns | No concerns | Some concerns | No concerns | Moderate | ["Heterogeneity"] |
| NEI:WQX | 3 | No concerns | Low risk | No concerns | No concerns | Some concerns | Some concerns | Low | ["Heterogeneity","Incoherence"] |
| NEI:YG | 2 | Some concerns | Low risk | No concerns | Some concerns | No concerns | No concerns | Low | ["Within-study bias","Imprecision"] |
| BDJ:CE | 0 | No concerns | Low risk | No concerns | No concerns | No concerns | Some concerns | Moderate | ["Incoherence"] |
| BDJ:PIL | 0 | Some concerns | Low risk | No concerns | Some concerns | No concerns | No concerns | Low | ["Within-study bias","Imprecision"] |
| BDJ:TC | 0 | No concerns | Low risk | No concerns | No concerns | Some concerns | No concerns | Moderate | ["Heterogeneity"] |
| BDJ:WQX | 0 | No concerns | Low risk | No concerns | Some concerns | Some concerns | Some concerns | Very low | ["Imprecision","Heterogeneity","Incoherence"] |
| BDJ:YG | 0 | Some concerns | Low risk | No concerns | Some concerns | No concerns | Some concerns | Very low | ["Within-study bias","Imprecision","Incoherence"] |
| BDJ:YJJ | 0 | No concerns | Low risk | No concerns | No concerns | No concerns | No concerns | High | [] |
| CE:NEI | 0 | Some concerns | Low risk | No concerns | No concerns | No concerns | Some concerns | Low | ["Within-study bias","Incoherence"] |
| CE:WQX | 0 | Some concerns | Low risk | No concerns | No concerns | Some concerns | Some concerns | Very low | ["Within-study bias","Heterogeneity","Incoherence"] |
| NEI:PIL | 0 | Some concerns | Low risk | No concerns | Some concerns | No concerns | Some concerns | Very low | ["Within-study bias","Imprecision","Incoherence"] |
| NEI:YJJ | 0 | No concerns | Low risk | No concerns | No concerns | No concerns | Some concerns | Moderate | ["Incoherence"] |
| PIL:TC | 0 | Some concerns | Low risk | No concerns | Some concerns | No concerns | No concerns | Low | ["Within-study bias","Imprecision"] |
| PIL:WQX | 0 | Some concerns | Low risk | No concerns | No concerns | No concerns | Some concerns | Low | ["Within-study bias","Incoherence"] |
| PIL:YG | 0 | Some concerns | Low risk | No concerns | Some concerns | No concerns | Some concerns | Very low | ["Within-study bias","Imprecision","Incoherence"] |
| PIL:YJJ | 0 | No concerns | Low risk | No concerns | Some concerns | No concerns | No concerns | Moderate | ["Imprecision"] |
| TC:WQX | 0 | No concerns | Low risk | No concerns | No concerns | Some concerns | Some concerns | Low | ["Heterogeneity","Incoherence"] |
| TC:YG | 0 | Some concerns | Low risk | No concerns | Some concerns | Some concerns | No concerns | Very low | ["Within-study bias","Imprecision","Heterogeneity"] |
| TC:YJJ | 0 | No concerns | Low risk | No concerns | Some concerns | No concerns | Some concerns | Low | ["Imprecision","Incoherence"] |
| WQX:YG | 0 | Some concerns | Low risk | No concerns | No concerns | No concerns | Some concerns | Low | ["Within-study bias","Incoherence"] |
| WQX:YJJ | 0 | No concerns | Low risk | No concerns | Some concerns | No concerns | No concerns | Moderate | ["Imprecision"] |
| YG:YJJ | 0 | No concerns | Low risk | No concerns | No concerns | No concerns | No concerns | High | [] |

**Table S42** Grade Grading Evaluation Form of WOMAC-Physical function

| **Comparison** | **Number of studies** | **Within-study bias** | **Reporting bias** | **Indirectness** | **Imprecision** | **Heterogeneity** | **Incoherence** | **Confidence rating** | **Reason(s) for downgrading** |
| --- | --- | --- | --- | --- | --- | --- | --- | --- | --- |
| BDJ:NEI | 3 | No concerns | Low risk | No concerns | No concerns | No concerns | Some concerns | Moderate | ["Incoherence"] |
| CE:PIL | 2 | Some concerns | Low risk | No concerns | No concerns | No concerns | Some concerns | Low | ["Within-study bias","Incoherence"] |
| CE:TC | 4 | Some concerns | Low risk | No concerns | No concerns | No concerns | No concerns | Moderate | ["Within-study bias"] |
| CE:YG | 2 | No concerns | Low risk | No concerns | No concerns | No concerns | No concerns | High | [] |
| CE:YJJ | 1 | No concerns | Low risk | No concerns | Some concerns | No concerns | Some concerns | Low | ["Imprecision","Incoherence"] |
| NEI:TC | 7 | No concerns | Low risk | No concerns | No concerns | Some concerns | No concerns | Moderate | ["Heterogeneity"] |
| NEI:WQX | 2 | No concerns | Low risk | No concerns | Some concerns | Some concerns | Some concerns | Very low | ["Imprecision","Heterogeneity","Incoherence"] |
| NEI:YG | 2 | Some concerns | Low risk | No concerns | No concerns | Some concerns | No concerns | Low | ["Within-study bias","Heterogeneity"] |
| BDJ:CE | 0 | No concerns | Low risk | No concerns | No concerns | No concerns | Some concerns | Moderate | ["Incoherence"] |
| BDJ:PIL | 0 | Some concerns | Low risk | No concerns | Some concerns | No concerns | Some concerns | Very low | ["Within-study bias","Imprecision","Incoherence"] |
| BDJ:TC | 0 | No concerns | Low risk | No concerns | Some concerns | No concerns | Some concerns | Low | ["Imprecision","Incoherence"] |
| BDJ:WQX | 0 | No concerns | Low risk | No concerns | No concerns | Some concerns | Some concerns | Low | ["Heterogeneity","Incoherence"] |
| BDJ:YG | 0 | Some concerns | Low risk | No concerns | Some concerns | No concerns | Some concerns | Very low | ["Within-study bias","Imprecision","Incoherence"] |
| BDJ:YJJ | 0 | No concerns | Low risk | No concerns | No concerns | Some concerns | Some concerns | Low | ["Heterogeneity","Incoherence"] |
| CE:NEI | 0 | Some concerns | Low risk | No concerns | Some concerns | No concerns | Some concerns | Very low | ["Within-study bias","Imprecision","Incoherence"] |
| CE:WQX | 0 | No concerns | Low risk | No concerns | Some concerns | No concerns | Some concerns | Low | ["Imprecision","Incoherence"] |
| NEI:PIL | 0 | Some concerns | Low risk | No concerns | No concerns | No concerns | Some concerns | Low | ["Within-study bias","Incoherence"] |
| NEI:YJJ | 0 | No concerns | Low risk | No concerns | Some concerns | No concerns | Some concerns | Low | ["Imprecision","Incoherence"] |
| PIL:TC | 0 | Some concerns | Low risk | No concerns | Some concerns | No concerns | Some concerns | Very low | ["Within-study bias","Imprecision","Incoherence"] |
| PIL:WQX | 0 | No concerns | Low risk | No concerns | No concerns | Some concerns | Some concerns | Low | ["Heterogeneity","Incoherence"] |
| PIL:YG | 0 | Some concerns | Low risk | No concerns | Some concerns | No concerns | Some concerns | Very low | ["Within-study bias","Imprecision","Incoherence"] |
| PIL:YJJ | 0 | No concerns | Low risk | No concerns | No concerns | No concerns | Some concerns | Moderate | ["Incoherence"] |
| TC:WQX | 0 | No concerns | Low risk | No concerns | Some concerns | No concerns | Some concerns | Low | ["Imprecision","Incoherence"] |
| TC:YG | 0 | Some concerns | Low risk | No concerns | No concerns | Some concerns | Some concerns | Very low | ["Within-study bias","Heterogeneity","Incoherence"] |
| TC:YJJ | 0 | No concerns | Low risk | No concerns | Some concerns | No concerns | Some concerns | Low | ["Imprecision","Incoherence"] |
| WQX:YG | 0 | No concerns | Low risk | No concerns | Some concerns | No concerns | Some concerns | Low | ["Imprecision","Incoherence"] |
| WQX:YJJ | 0 | No concerns | Low risk | No concerns | Some concerns | No concerns | Some concerns | Low | ["Imprecision","Incoherence"] |
| YG:YJJ | 0 | No concerns | Low risk | No concerns | Some concerns | No concerns | Some concerns | Low | ["Imprecision","Incoherence"] |

**Table S43** Grade Grading Evaluation Form of WOMAC-Stiffness

| **Comparison** | **Number of studies** | **Within-study bias** | **Reporting bias** | **Indirectness** | **Imprecision** | **Heterogeneity** | **Incoherence** | **Confidence rating** | **Reason(s) for downgrading** |
| --- | --- | --- | --- | --- | --- | --- | --- | --- | --- |
| BDJ:NEI | 3 | No concerns | Low risk | No concerns | No concerns | No concerns | No concerns | High | [] |
| CE:PIL | 1 | No concerns | Low risk | No concerns | No concerns | Some concerns | No concerns | Moderate | ["Heterogeneity"] |
| CE:TC | 4 | Some concerns | Low risk | No concerns | No concerns | Some concerns | No concerns | Low | ["Within-study bias","Heterogeneity"] |
| CE:YG | 2 | Some concerns | Low risk | No concerns | No concerns | No concerns | No concerns | Moderate | ["Within-study bias"] |
| CE:YJJ | 1 | No concerns | Low risk | No concerns | Some concerns | No concerns | No concerns | Moderate | ["Imprecision"] |
| NEI:TC | 6 | No concerns | Low risk | No concerns | No concerns | Some concerns | No concerns | Moderate | ["Heterogeneity"] |
| NEI:WQX | 3 | No concerns | Low risk | No concerns | Some concerns | No concerns | No concerns | Moderate | ["Imprecision"] |
| NEI:YG | 2 | Some concerns | Low risk | No concerns | Some concerns | No concerns | No concerns | Low | ["Within-study bias","Imprecision"] |
| BDJ:CE | 0 | Some concerns | Low risk | No concerns | No concerns | No concerns | No concerns | Moderate | ["Within-study bias"] |
| BDJ:PIL | 0 | No concerns | Low risk | No concerns | Some concerns | No concerns | No concerns | Moderate | ["Imprecision"] |
| BDJ:TC | 0 | No concerns | Low risk | No concerns | No concerns | No concerns | No concerns | High | [] |
| BDJ:WQX | 0 | No concerns | Low risk | No concerns | No concerns | No concerns | No concerns | High | [] |
| BDJ:YG | 0 | Some concerns | Low risk | No concerns | No concerns | No concerns | No concerns | Moderate | ["Within-study bias"] |
| BDJ:YJJ | 0 | No concerns | Low risk | No concerns | No concerns | No concerns | No concerns | High | [] |
| CE:NEI | 0 | Some concerns | Low risk | No concerns | No concerns | Some concerns | No concerns | Low | ["Within-study bias","Heterogeneity"] |
| CE:WQX | 0 | Some concerns | Low risk | No concerns | No concerns | Some concerns | No concerns | Low | ["Within-study bias","Heterogeneity"] |
| NEI:PIL | 0 | No concerns | Low risk | No concerns | Some concerns | Some concerns | No concerns | Low | ["Imprecision","Heterogeneity"] |
| NEI:YJJ | 0 | No concerns | Low risk | No concerns | Some concerns | No concerns | No concerns | Moderate | ["Imprecision"] |
| PIL:TC | 0 | No concerns | Low risk | No concerns | Some concerns | No concerns | No concerns | Moderate | ["Imprecision"] |
| PIL:WQX | 0 | No concerns | Low risk | No concerns | Some concerns | No concerns | No concerns | Moderate | ["Imprecision"] |
| PIL:YG | 0 | Some concerns | Low risk | No concerns | Some concerns | No concerns | No concerns | Low | ["Within-study bias","Imprecision"] |
| PIL:YJJ | 0 | No concerns | Low risk | No concerns | Some concerns | Some concerns | No concerns | Low | ["Imprecision","Heterogeneity"] |
| TC:WQX | 0 | No concerns | Low risk | No concerns | Some concerns | Some concerns | No concerns | Low | ["Imprecision","Heterogeneity"] |
| TC:YG | 0 | Some concerns | Low risk | No concerns | Some concerns | Some concerns | No concerns | Very low | ["Within-study bias","Imprecision","Heterogeneity"] |
| TC:YJJ | 0 | No concerns | Low risk | No concerns | Some concerns | Some concerns | No concerns | Moderate | ["Imprecision","Heterogeneity"] |
| WQX:YG | 0 | Some concerns | Low risk | No concerns | Some concerns | Some concerns | No concerns | Very low | ["Within-study bias","Imprecision","Heterogeneity"] |
| WQX:YJJ | 0 | No concerns | Low risk | No concerns | Some concerns | Some concerns | No concerns | Low | ["Imprecision","Heterogeneity"] |
| YG:YJJ | 0 | Some concerns | Low risk | No concerns | Some concerns | No concerns | No concerns | Low | ["Within-study bias","Imprecision"] |

**Table S44** Grade Grading Evaluation Form of 6MWT(6-min walk test)

| **Comparison** | **Number of studies** | **Within-study bias** | **Reporting bias** | **Indirectness** | **Imprecision** | **Heterogeneity** | **Incoherence** | **Confidence rating** | **Reason(s) for downgrading** |
| --- | --- | --- | --- | --- | --- | --- | --- | --- | --- |
| CE:NEI | 2 | Some concerns | Low risk | No concerns | Some concerns | Some concerns | No concerns | Very low | ["Within-study bias","Imprecision","Heterogeneity"] |
| CE:TC | 4 | Some concerns | Low risk | No concerns | No concerns | Some concerns | No concerns | Low | ["Within-study bias","Heterogeneity"] |
| CE:YG | 1 | No concerns | Low risk | No concerns | Some concerns | No concerns | No concerns | Moderate | ["Imprecision"] |
| NEI:TC | 4 | Some concerns | Low risk | No concerns | Some concerns | No concerns | No concerns | Low | ["Within-study bias","Imprecision"] |
| NEI:WQX | 2 | No concerns | Low risk | No concerns | Some concerns | Some concerns | No concerns | Low | ["Imprecision","Heterogeneity"] |
| NEI:YG | 1 | No concerns | Low risk | No concerns | Some concerns | No concerns | No concerns | Moderate | ["Imprecision"] |
| CE:WQX | 0 | No concerns | Low risk | No concerns | Some concerns | Some concerns | No concerns | Low | ["Imprecision","Heterogeneity"] |
| TC:WQX | 0 | No concerns | Low risk | No concerns | Some concerns | Some concerns | No concerns | Low | ["Imprecision","Heterogeneity"] |
| TC:YG | 0 | No concerns | Low risk | No concerns | Some concerns | No concerns | No concerns | Moderate | ["Imprecision"] |
| WQX:YG | 0 | No concerns | Low risk | No concerns | Some concerns | No concerns | No concerns | Moderate | ["Imprecision"] |

**Table S45** **Baseline and Intervention-Related Characteristics of the Included Studies**

Note. ACR = American College of Rheumatology; K-L = Kellgren-Lawrence; KOA = Knee Osteoarthritis; WOMAC = Western Ontario and McMaster Universities Osteoarthritis Index; KOOS = Knee Injury and Osteoarthritis Outcome Score; VAS = Visual Analog Scale; NRS = Numeric Rating Scale; SF-36 = Short Form-36; LEFS = Lower Extremity Functional Scale; PT = Physical Therapy; NSAIDs = Non-Steroidal Anti-Inflammatory Drugs; TENS = Transcutaneous Electrical Nerve Stimulation; RA = Rheumatoid Arthritis; NICE = National Institute for Health and Care Excellence

| Study (Year) | Comparison | Disease Severity | Pain Level | Supervision Intensity | Exercise Dose | Concurrent Therapy |
| --- | --- | --- | --- | --- | --- | --- |
| Tsai et al. 2013 | Tai Chi vs. Control | ACR criteria, cognitive impairment | Balanced (WOMAC pain baseline, p=0.433) | Full on-site (both groups) | Tai Chi: 2×60min×20wControl: 1×60min×20w education | Routine analgesics |
| An et al. 2008 | Baduanjin vs. Waitlist | ACR criteria | Balanced (WOMAC pain baseline, p=0.401) | Baduanjin: Full on-siteControl: None | Baduanjin: 5×30min×8wControl: None | No medication changes |
| Wang et al. 2016 | Tai Chi vs. PT | ACR criteria, K-L 2-4 | balanced (WOMAC pain baseline, p=0.721) | Tai Chi: Full on-sitePT: Mixed (outpatient + home) | Tai Chi: 2×60min×12wPT: 2×30min×6w + 4×30min×6w | Routine NSAIDs |
| Saleem et al. 2022 | Pilates vs. Isometric | K-L II-III | Balanced (WOMAC pain baseline, p=0.131) | Full on-site (both groups) | Both: 3×60min×8w (incl. hot compress + TENS) | Hot compress + TENS + routine meds |
| Bennell et al. 2022 | Online Yoga vs. Education | NICE criteria | balanced (NRS walking pain baseline, p=0.925; WOMAC pain baseline, p=0.968) | No supervision (both groups) | Yoga: 3×30min×12wControl: Education only | Routine treatments |
| Rêgo et al. 2023 | Pilates vs. Waitlist | K-L II-III | balanced (WOMAC pain baseline, p=0.512) | Pilates: Full on-siteControl: Telephone only | Pilates: 2×60min×7wControl: None | Medication recorded |
| Wortley et al. 2013 | Resistance training vs. Tai Ji vs. Control | ACR criteria, K-L 1-4 | balanced (WOMAC pain baseline, p=0.702) | RT/TJ: Full on-siteControl: Telephone only | RT: 2×60min×10wTJ: 2×60min×10wControl: 1×telephone contact | No medication changes |
| Lee et al. 2017 | Tai Chi vs. PT | ACR criteria, K-L 0-4, WOMAC pain ≥40 | balanced (WOMAC pain baseline, p=0.520) | Tai Chi: Full on-sitePT: Mixed (outpatient + home) | Tai Chi: 2×60min×12w + ≥20min/day homePT: 2×30min×6w + 4×30min×6w home + ≥20min/day home | Routine analgesics; no intra-articular injections in past 3/6 months |
| Zhu et al. 2016 | Tai Ji Quan vs. Education control | ACR criteria, K-L 1-3 | Balanced (WOMAC pain baseline, p=0.699) | Tai Ji: Full on-siteControl: Bi-weekly lectures + weekly check-in | Tai Ji: 3×60min×24wControl: 1×60min×24w (bi-weekly) | Usual medications; no new exercise programs |
| Hu et al. 2020 | Taichi vs. Health education control | ACR criteria, K-L ≥1 | balanced (VAS pain baseline, p=0.317; WOMAC pain baseline, p=0.826) | Taichi: Full on-siteControl: Bi-weekly lectures | Taichi: 3×60min×24wControl: 1×60min×24w (bi-weekly) | Usual lifestyle; no other regular exercise |
| Xiao et al. 2020a | Wu Qin Xi Qigong vs. Conventional PT | ACR criteria, K-L I-II | balanced (WOMAC pain baseline, p=0.847) | Both groups: Full on-site | Both: 4×60min×24w | Usual medications; no other therapies |
| Kuntz et al. 2018 | Biomechanical yoga vs. Traditional exercise vs. Meditation control | ACR criteria, LEFS 30-65 (mild-moderate) | balanced (KOOS pain baseline, p=0.712) | All groups: Full on-site | All: 3×60min×12w | Routine analgesics; no corticosteroid injections during study |
| Xiao et al. 2020b | Wuqinxi home exercise vs. Conventional PT home exercise | ACR criteria, K-L I-II | balanced (WOMAC pain baseline, p=0.847) | Initial on-site training, then home-based (both groups) | Both: 4×60min×12w (home practice) | Usual medications; no other structured exercise |
| Kang et al. 2022 | Tai Chi vs. Education control | ACR criteria for KOA | balanced (VAS pain baseline, p=0.547) | Tai Chi: Full on-site supervisionControl: Bi-weekly on-site lectures | Tai Chi: 3×60min×24wControl: 1×60min×24w (bi-weekly) | Usual lifestyle; no other regular exercise |
| Brismee et al. 2006 | Tai Chi vs. Attention control (health lectures) | ACR criteria for KOA | balanced (VAS pain & WOMAC pain baseline, p=0.780) | Tai Chi: First 6w full on-site, next 6w home video practiceControl: First 6w on-site lectures | Tai Chi: 3×40min×6w (group) + 3×40min×6w (home)Control: 3×40min×6w (lectures) | Usual medications; no dose changes during study |
| Evans et al. 2013 | Iyengar Yoga vs. Waitlist control | ACR criteria for RA, mean disease duration 10.6 years | balanced (Pain baseline, p=0.698) | Yoga: Full on-site supervisionControl: No supervision | Yoga: 2×75min×6w + home practiceControl: No intervention | Usual pharmacotherapy; no medication changes during study |
| Cheung et al. 2016 | Hatha Yoga (HY) vs. Aerobic/strengthening exercise (ASE) vs. Education control | ACR criteria for KOA, symptomatic ≥6 months | balanced (WOMAC pain baseline, p=0.20) | HY/ASE: Full on-site supervisionControl: Weekly telephone follow-up | HY: 1×45min×8w + 4×30min/w home practiceASE: 1×45min×8w + 4×15-30min/w aerobic + 2×30min/w strength home practiceControl: Weekly telephone calls | Usual medications; no intra-articular injections or surgery during study |
| Ye et al. 2020a | Baduanjin + Usual care vs. Usual care control | ACR criteria for KOA, K-L grade 2-3 | balanced (WOMAC pain baseline, p=0.517) | Baduanjin: First 4w full on-site, then home practice + telephone follow-upControl: Usual care only | Baduanjin: 3×40min×12w | Usual acupuncture, massage, moxibustion; no medication changes during study |
| Ni et al. 2010 | Tai Chi vs. Attention control (wellness education + stretching) | ACR criteria for KOA, symptomatic ≥1 year | balanced (WOMAC pain baseline, p=0.787) | Tai Chi: Full on-site supervisionControl: Weekly on-site sessions | Tai Chi: 2-4×30min×24w (frequency gradually increased)Control: 1×45min×24w | Usual diet; no new exercise or medication changes |
| Wang et al. 2009 | Tai Chi vs. Attention control (wellness education + stretching) | ACR criteria for KOA, K-L grade 2-4 | balanced (WOMAC pain baseline, p=0.721) | Both groups: Full on-site supervision | Tai Chi: 2×60min×12w + ≥20min/day home practiceControl: Same frequency and duration | Usual NSAIDs and analgesics; all medication changes recorded |
| Lee et al. 2009 | Tai Chi Qigong vs. Waitlist control | K-L grade 2-4, symptomatic KOA ≥6 months | balanced (WOMAC pain baseline, p=0.623) | Tai Chi Qigong: Full on-site supervisionControl: No intervention | Tai Chi Qigong: 2×60min×8w | No medications or injections in the 6 months prior to study |
| Song et al. 2022 | Modified Tai Chi vs. Wellness education | ACR criteria for KOA, K-L 0-3, elderly women | balanced (WOMAC pain baseline, p=0.970) | Tai Chi: Full on-site; Control: Bi-weekly lectures | Tai Chi: 3×60min×12w; Control: 1×60min×12w | Routine lifestyle; no other regular exercise |
| Zhang et al. 2022 | Yijinjing Qigong vs. Stretching training | ACR criteria for KOA, K-L 1-2 | balanced (WOMAC pain baseline, p=0.38) | Both groups: Full on-site | Both: 2×40min×12w | Routine pain meds, NSAIDs; no new interventions |
| Ye et al. 2020b | Baduanjin vs. Usual lifestyle control | ACR KOA criteria, K-L grade 2–3 | balanced (WOMAC pain baseline, p=0.528) | Baduanjin: First 4 weeks full on-site, then home practice + biweekly telephone follow-up; Control: No supervision | Baduanjin: 3×40min/week ×12 weeks; Control: No structured exercise | No musculoskeletal-affecting medications; no regular exercise during study |
| Nahayatbin et al. 2018 | Tai Chi + Routine physiotherapy vs. CKCE + Routine physiotherapy vs. Routine physiotherapy alone | K-L grade 2–3, Oxford muscle strength grade ≥3 | balanced (KOOS pain baseline, p=0.752) | All groups: Full on-site supervision during treatment sessions | All intervention groups: 3×20min/week ×4 weeks; All groups received routine physiotherapy | Routine physiotherapy (15min infrared + 5min ultrasound per session); no NSAIDs/corticosteroids in 1 month prior |
| Cheung et al. 2014 | Hatha Yoga vs. Wait-list control | ACR KOA criteria, symptomatic duration ≥6 months, community-dwelling older women | balanced (WOMAC pain baseline, p=0.25) | Yoga: Weekly on-site group sessions + home practice; Control: No supervision | Yoga: 1×60min/week ×8 weeks + 4×30min/week home practice; Control: No structured exercise | Routine medications; no intra-articular injections in 6 months prior; no knee surgery in 2 years prior |
| Xiao et al., 2021 | Wuqinxi group vs no regular exercise control | Elderly female KOA, duration >6 months | balanced (WOMAC pain baseline, p=0.86) | Professional guidance | 24 weeks, 6×/week, 60 min/session | Analgesics/cartilage protectants/physiotherapy permitted; identical in both groups |
| Zhu et al. 2025 | Online unsupervised Tai Chi vs. Online OA education | NICE KOA criteria, ≥45 years, symptomatic ≥3 months, walking pain NRS ≥4 | balanced (NRS walking pain & WOMAC pain baseline, p=0.892) | Tai Chi: Unsupervised online + app support; Control: Unsupervised online education | Tai Chi: 3×45min/week ×12 weeks (prerecorded video) | Usual meds & non-pharmacological treatments; all changes recorded |
| Abafita et al. 2025 | Yoga vs. Strengthening exercise | ACR KOA criteria, ≥40 years, knee pain VAS ≥40 mm | balanced (VAS knee pain baseline, p=0.76) | Week 1–12: 2×/week on-site + 1×/week home; Week 13–24: home unsupervised | Both: 2×60min on-site +1×60min home/week ×12w; 3×60min home/week ×12w | Usual pain medications; routine care allowed |

**Table S46 Heterogeneity statistics (I²) for all outcome measures included in the network meta-analysis**

| **outcome measures** | *I^2^* |
| --- | --- |
| TUG | 0.00 |
| Mental health | 64.20 |
| WOMAC-Pain | 68.80 |
| WOMAC-Physical function | 42.29 |
| WOMAC-Stiffness | 40.83 |
| 6MWT | 28.67 |

**
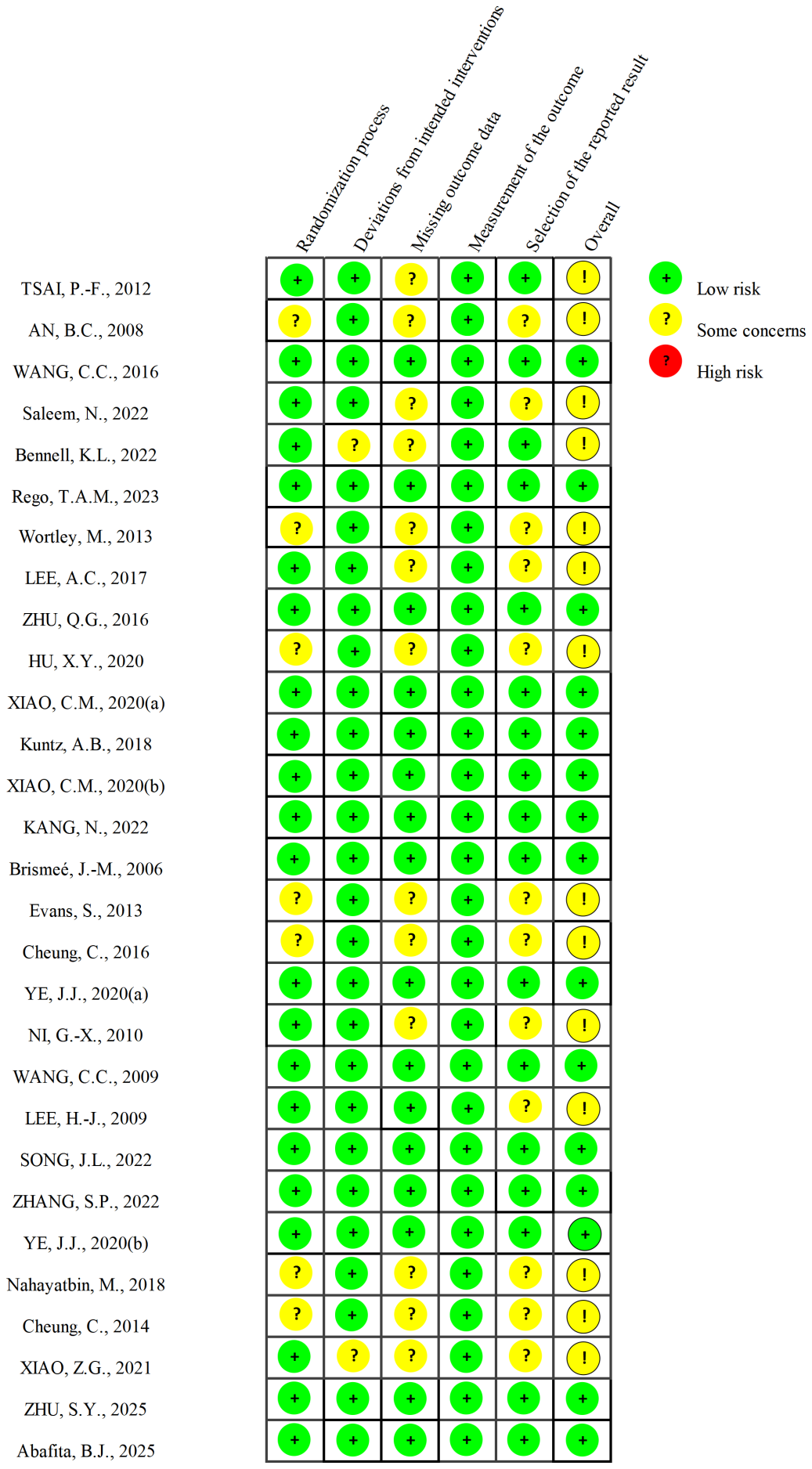
**

**Figure 1** The risk of bias summery graph according to Cochrane risk of bias tool for randomized trials (RoB2).

**Footnote**: XIAO, C.M. (a), XIAO, C.M. (b), YE, J.J. (a), and YE, J.J. (b) refer to two distinct studies, each employing different intervention approaches.

**
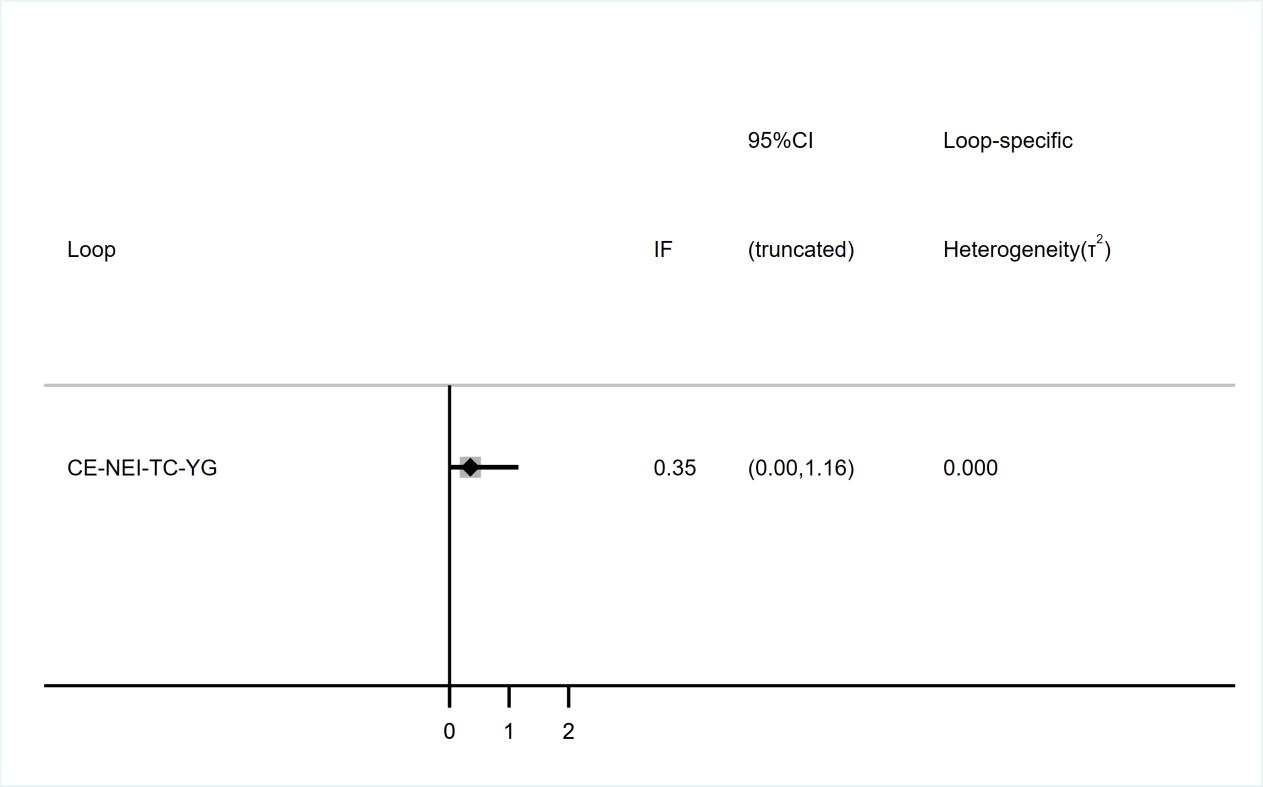
**

**Figure 2** Loop Inconsistency Diagram of the Outcome Measure Mental Health

**
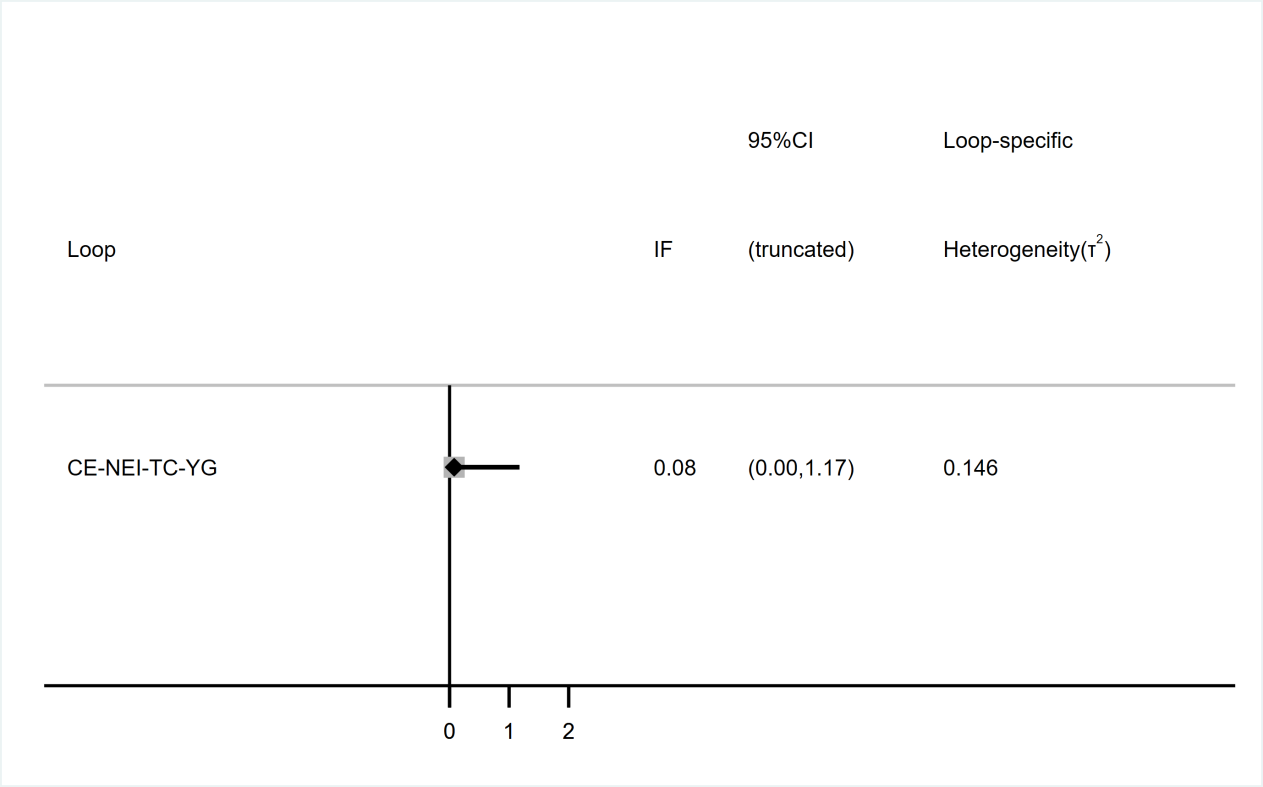
**

**Figure 3** Loop Inconsistency Diagram of the Outcome Measure WOMAC-Pain

**
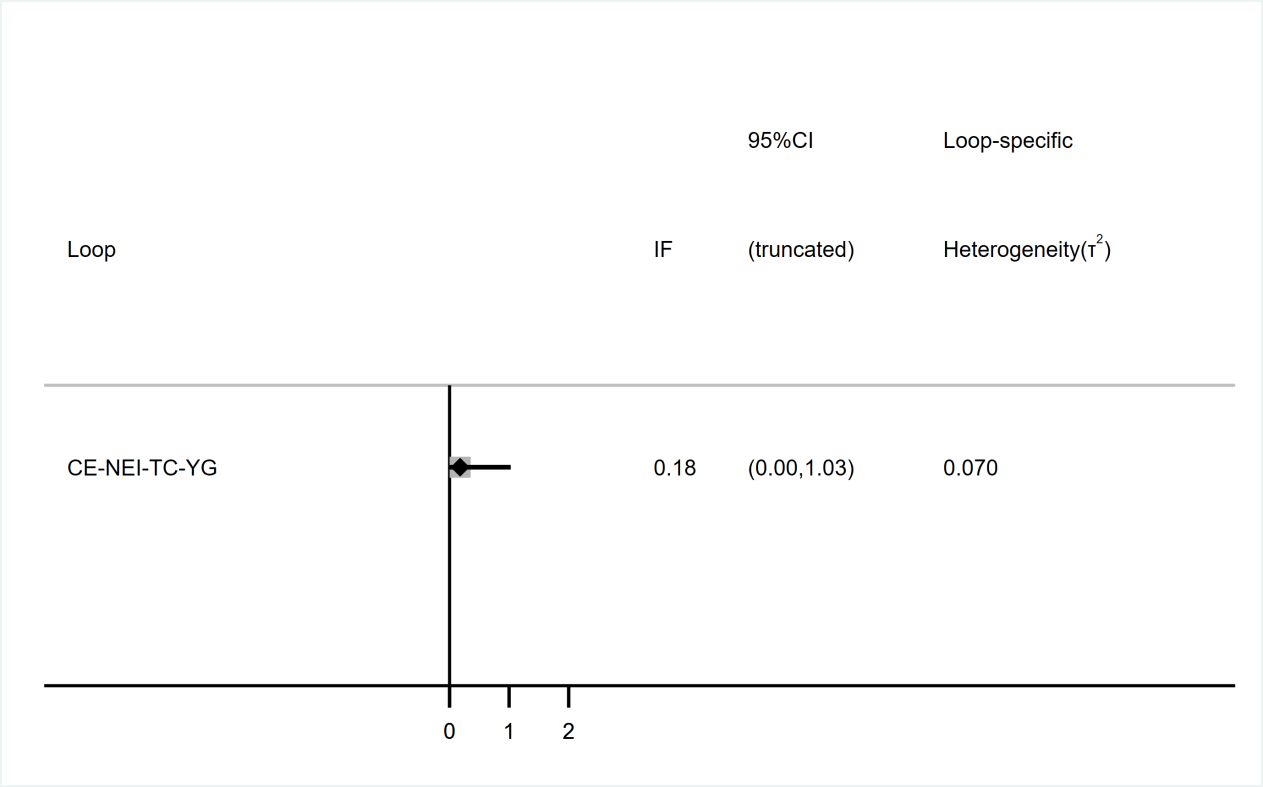
**

**Figure 4** Loop Inconsistency Diagram of the Outcome Measure WOMAC-Physical Function

**
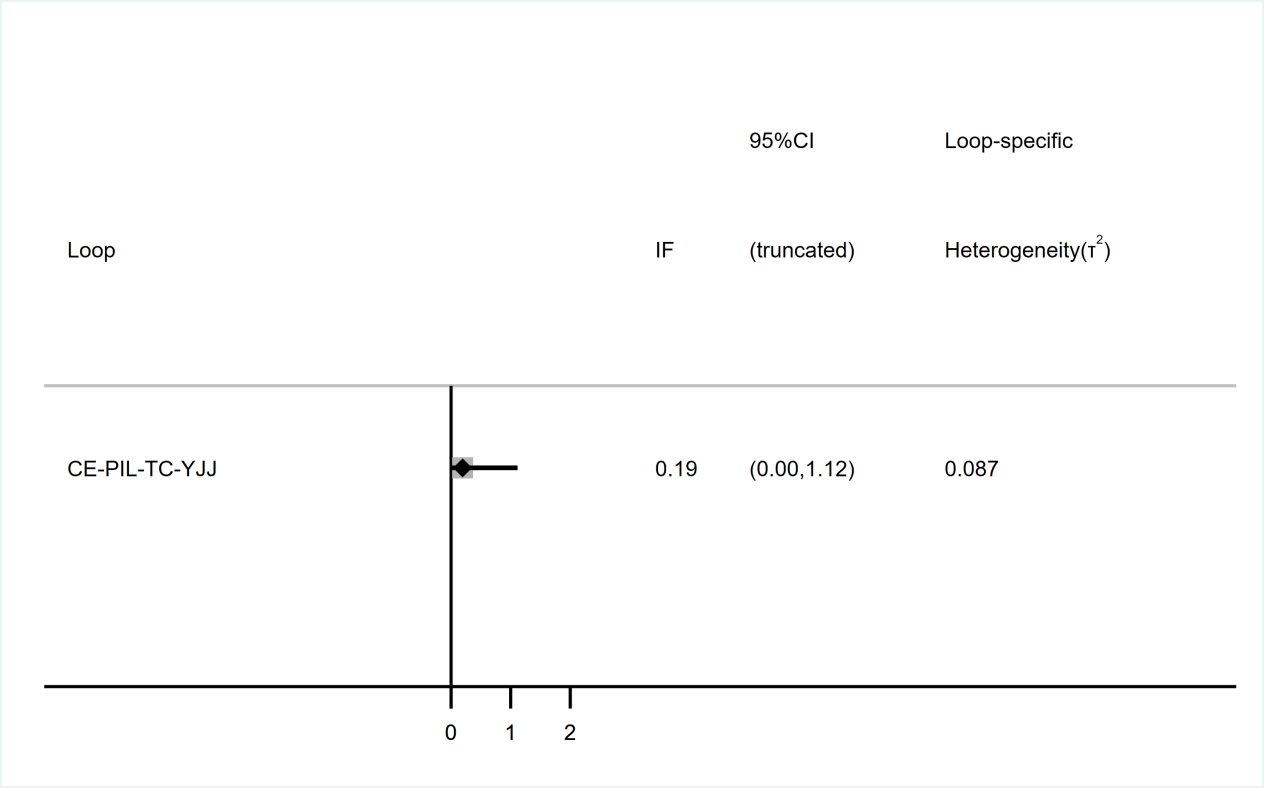
**

**Figure 5** Loop Inconsistency Diagram of the Outcome Measure WOMAC-Stiffness

**
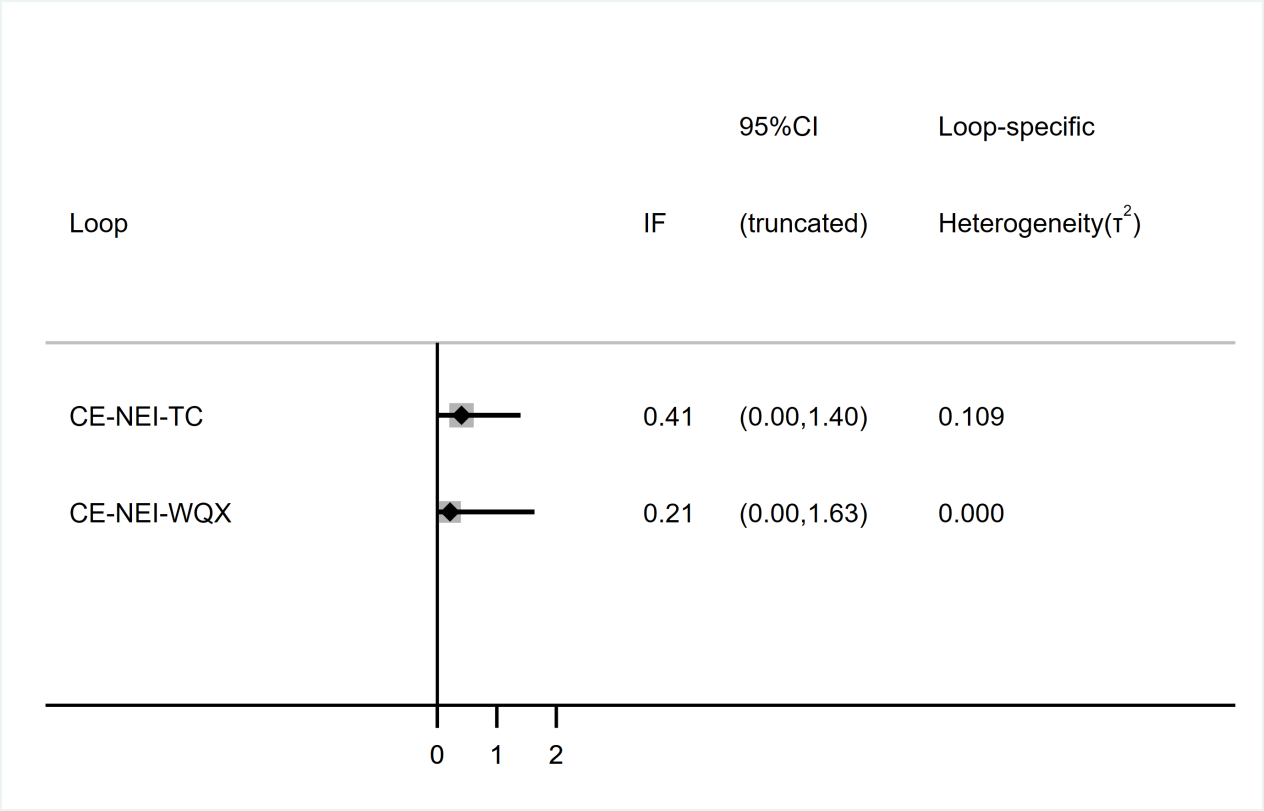
**

**Figure 6** Loop Inconsistency Diagram of the Outcome Measure 6MWT (6-Minute Walk Test)

**
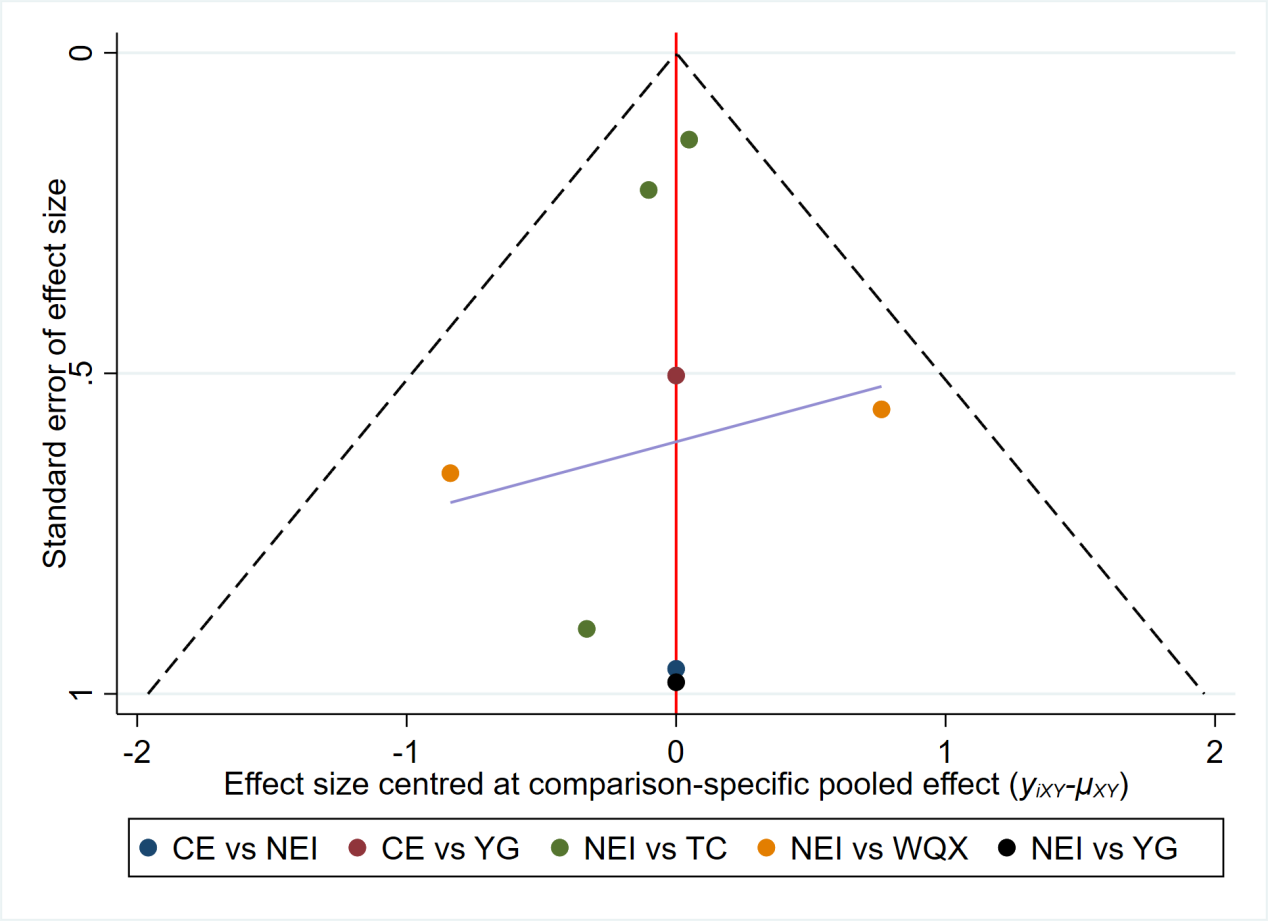
**

**Figure 7** Funnel Plot of the Outcome Measure TUG (Timed Up and Go)

**
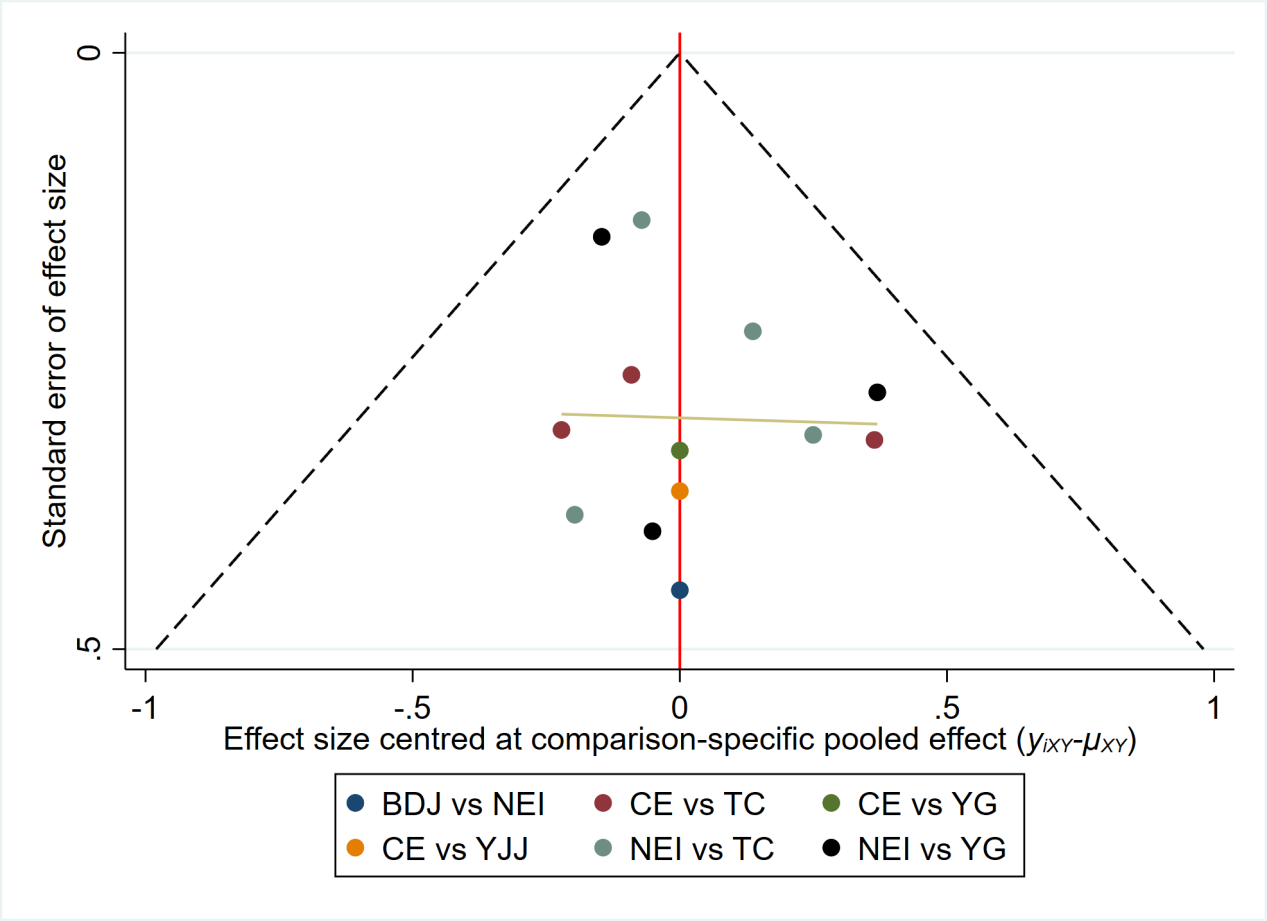
**

**Figure 8** Funnel Plot for the Outcome Mental Health

**
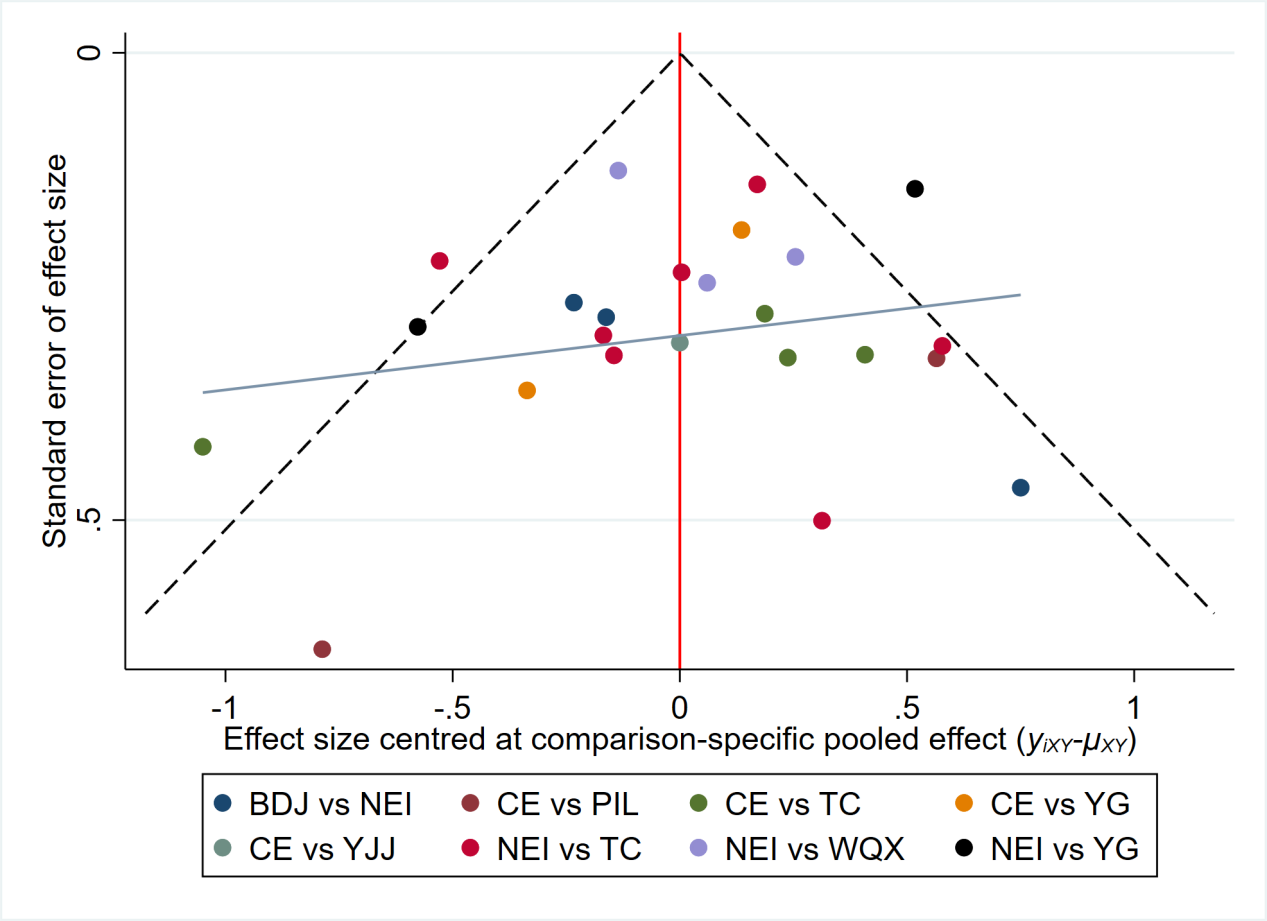
**

**Figure 9** Funnel Plot of the Outcome Measure WOMAC-Pain

**
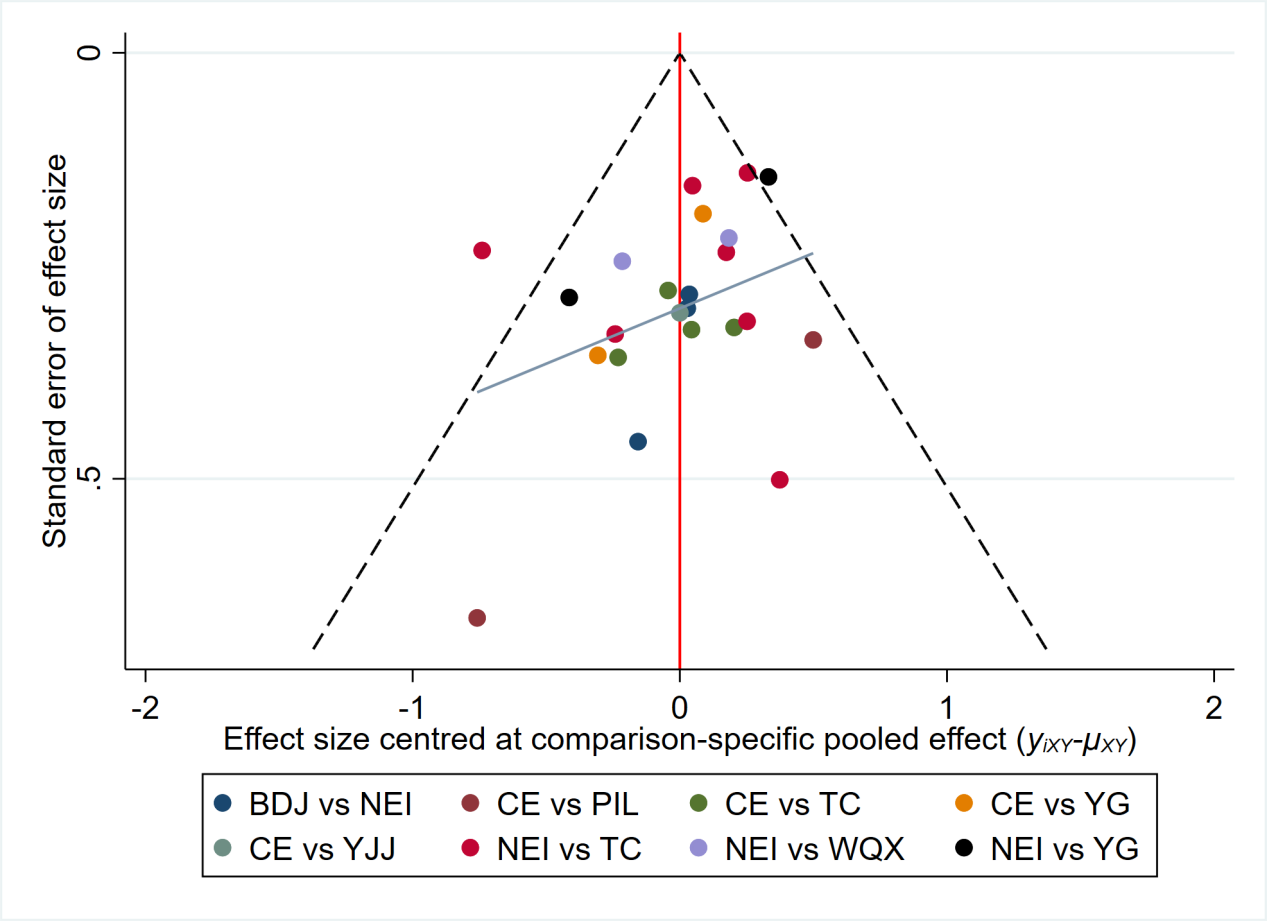
**

**Figure 10** Funnel Plot of the Outcome Measure WOMAC-Physical Function

**
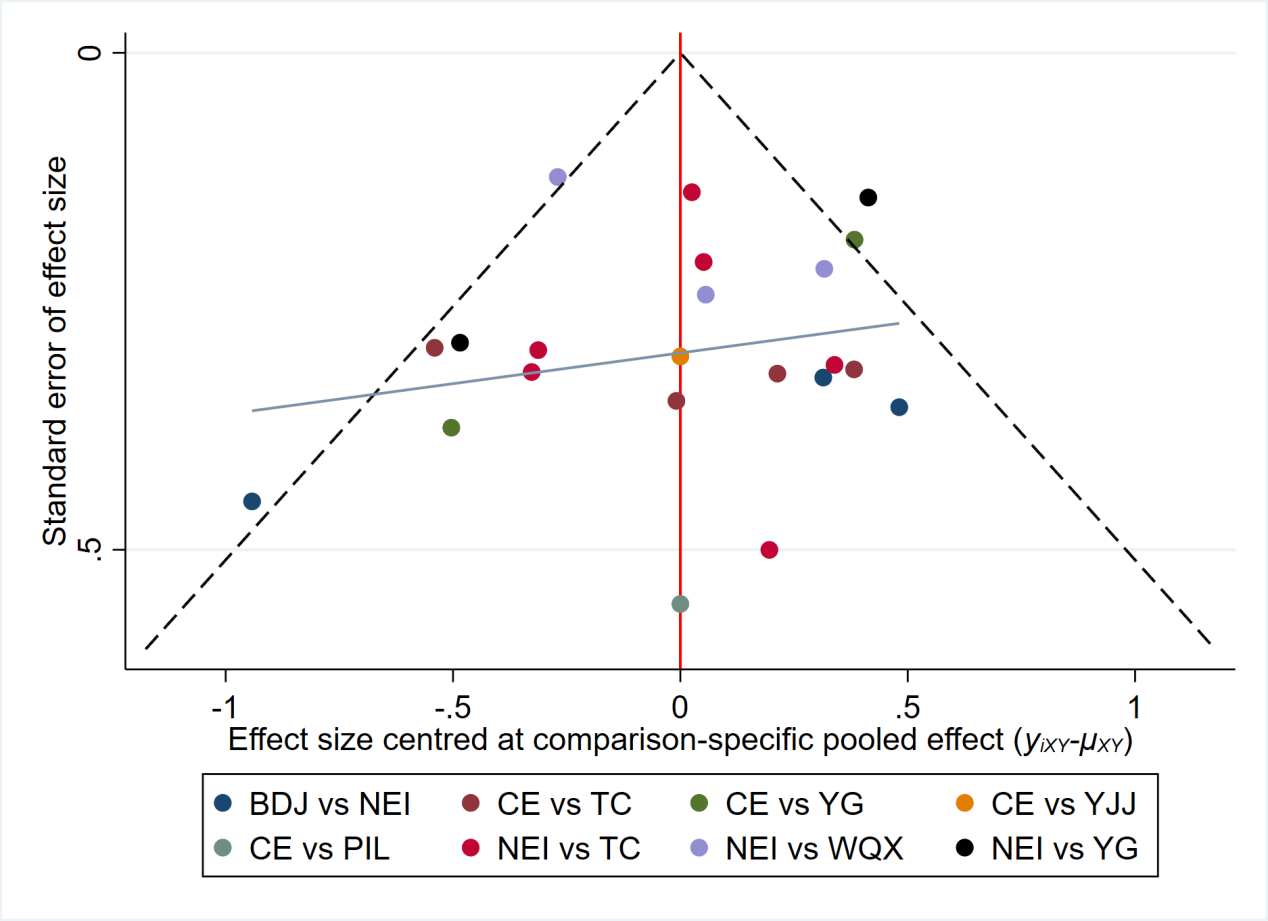
**

**Figure 11** Funnel Plot of the Outcome Measure WOMAC-Stiffness

**
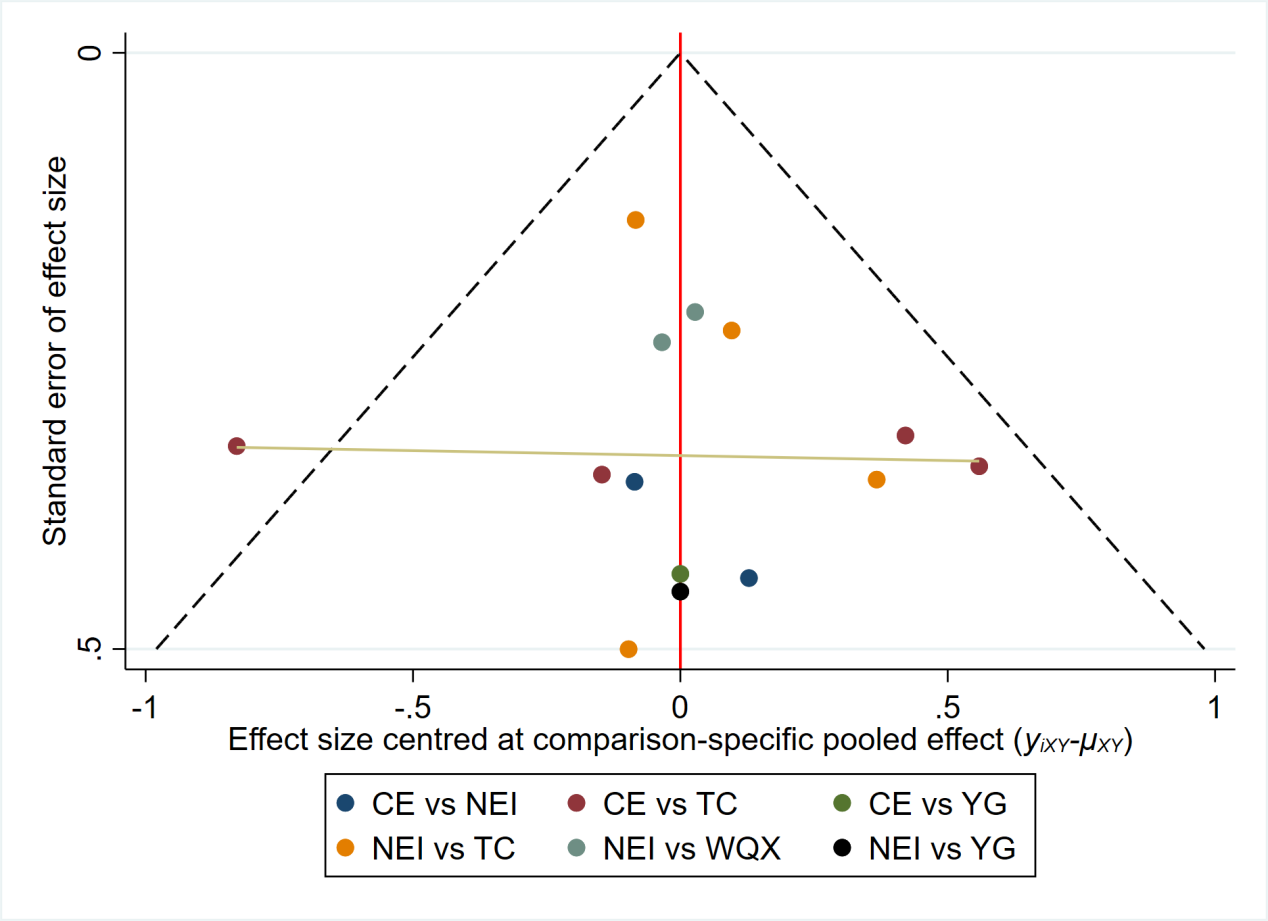
**

**Figure 12** Funnel Plot of the Outcome Measure 6MWT (6-Minute Walk Test)

**
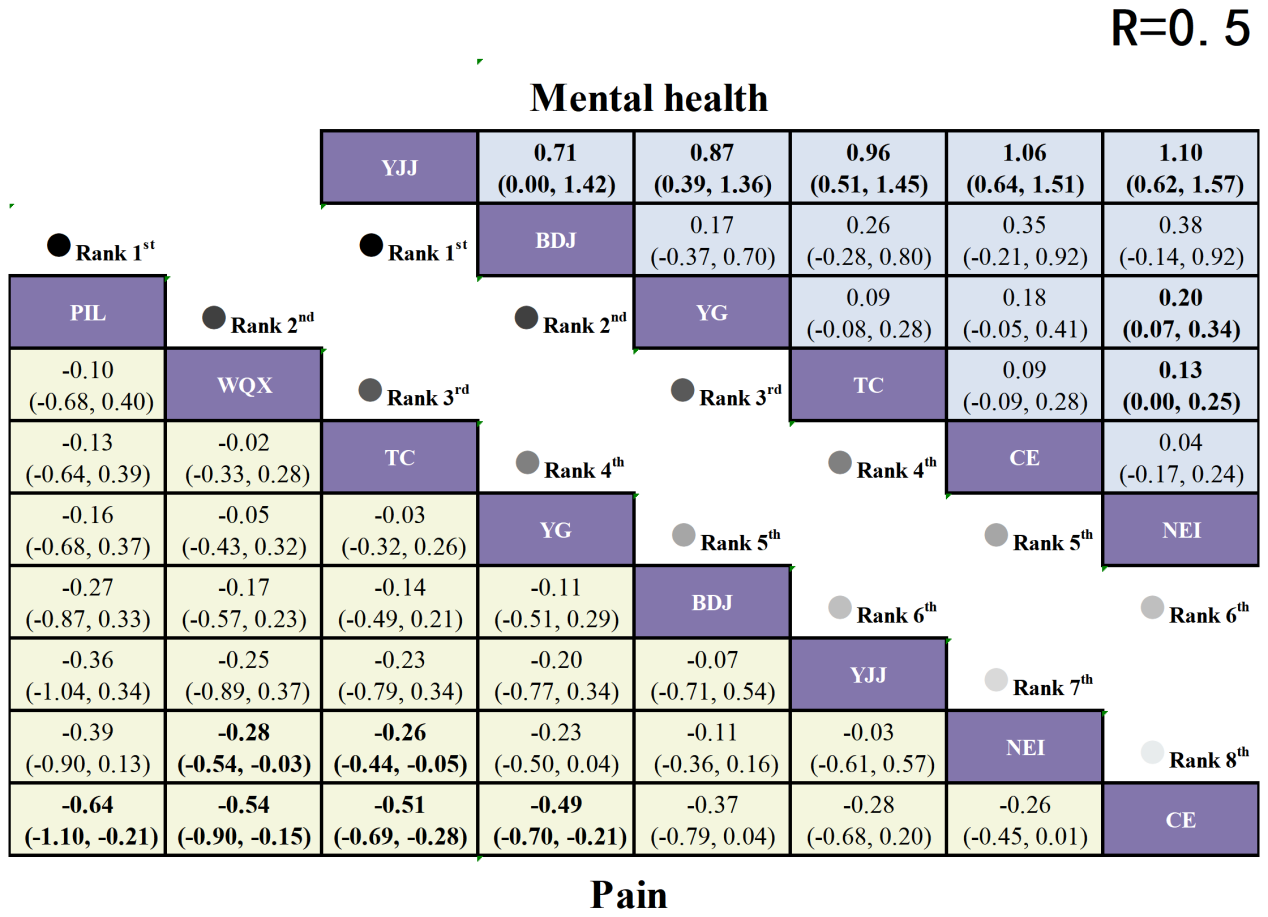
**

**Figure 13** League table of pairwise comparisons for Mental health and Pain outcomes based on correlation coefficient (R = 0.5)


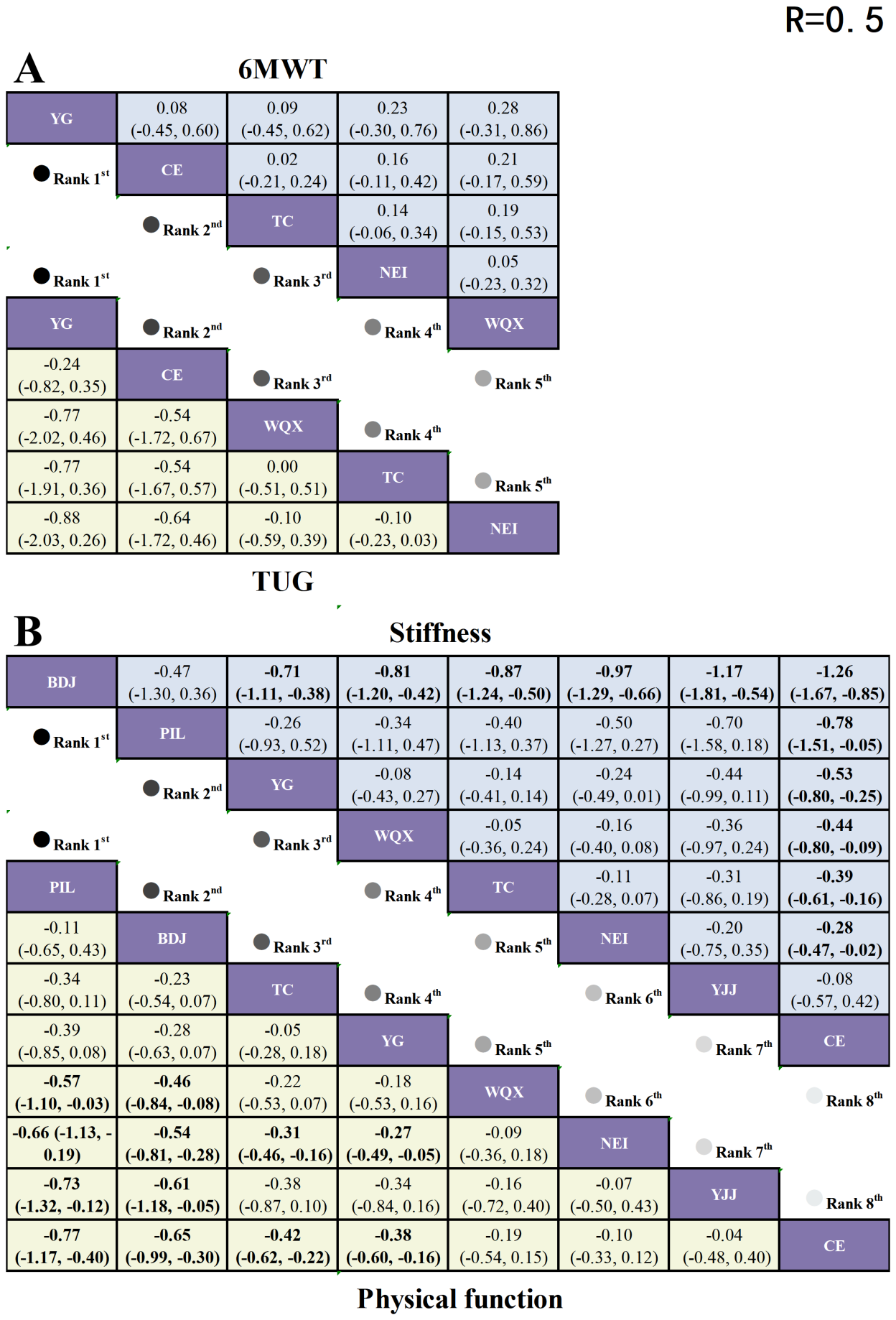


**Figure 14** League table of pairwise comparisons for all outcomes (R = 0.5)

Note: This league table presents pairwise comparisons of interventions across four outcomes (6MWT, TUG, Stiffness, and Physical function) using a correlation coefficient of 0.5 for imputing change-score standard deviations. Panel A displays results for 6MWT and TUG; Panel B displays results for Stiffness and Physical function. Values represent mean differences with 95% confidence intervals.


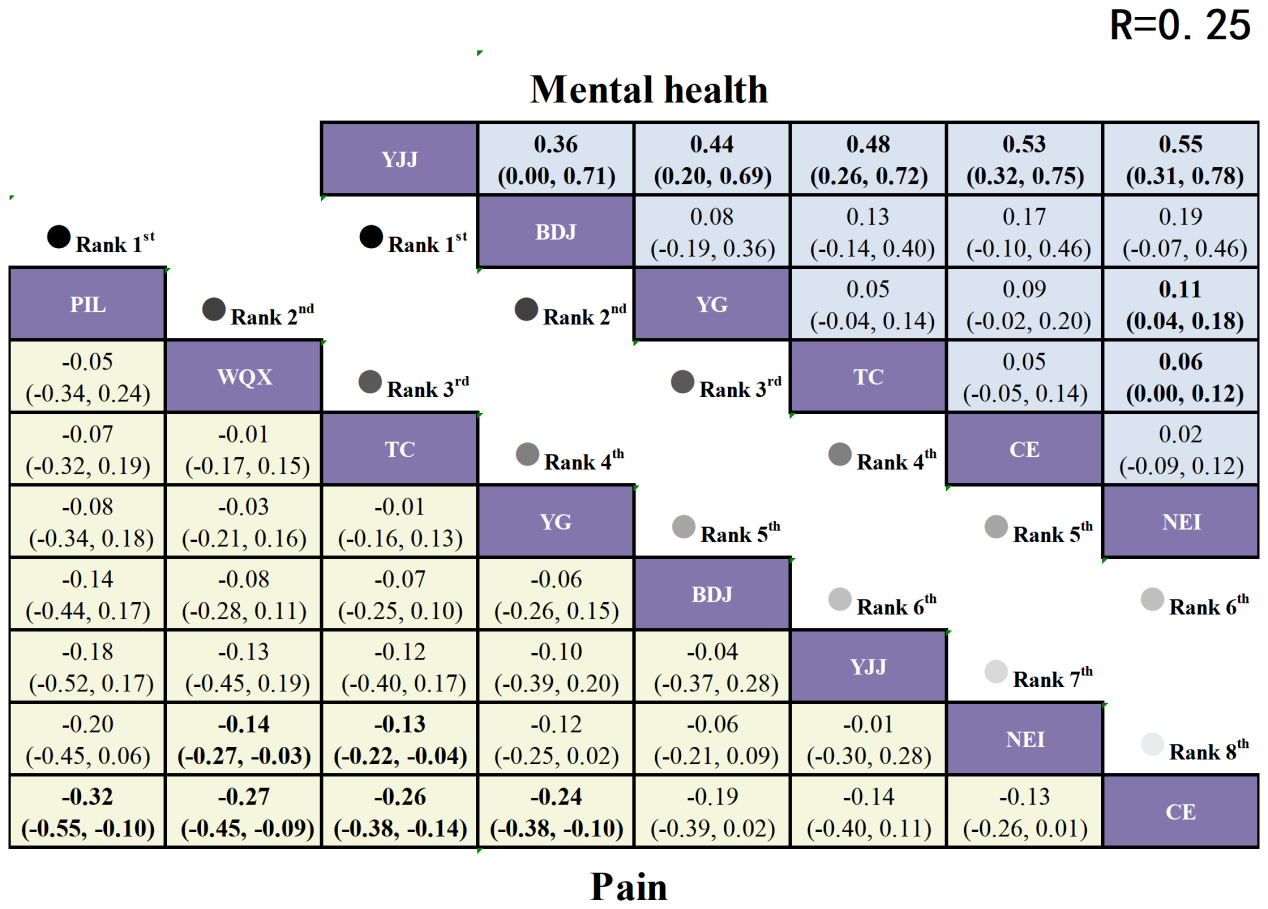


**Figure 15** League table of pairwise comparisons for Mental health and Pain outcomes based on correlation coefficient (R = 0.25)


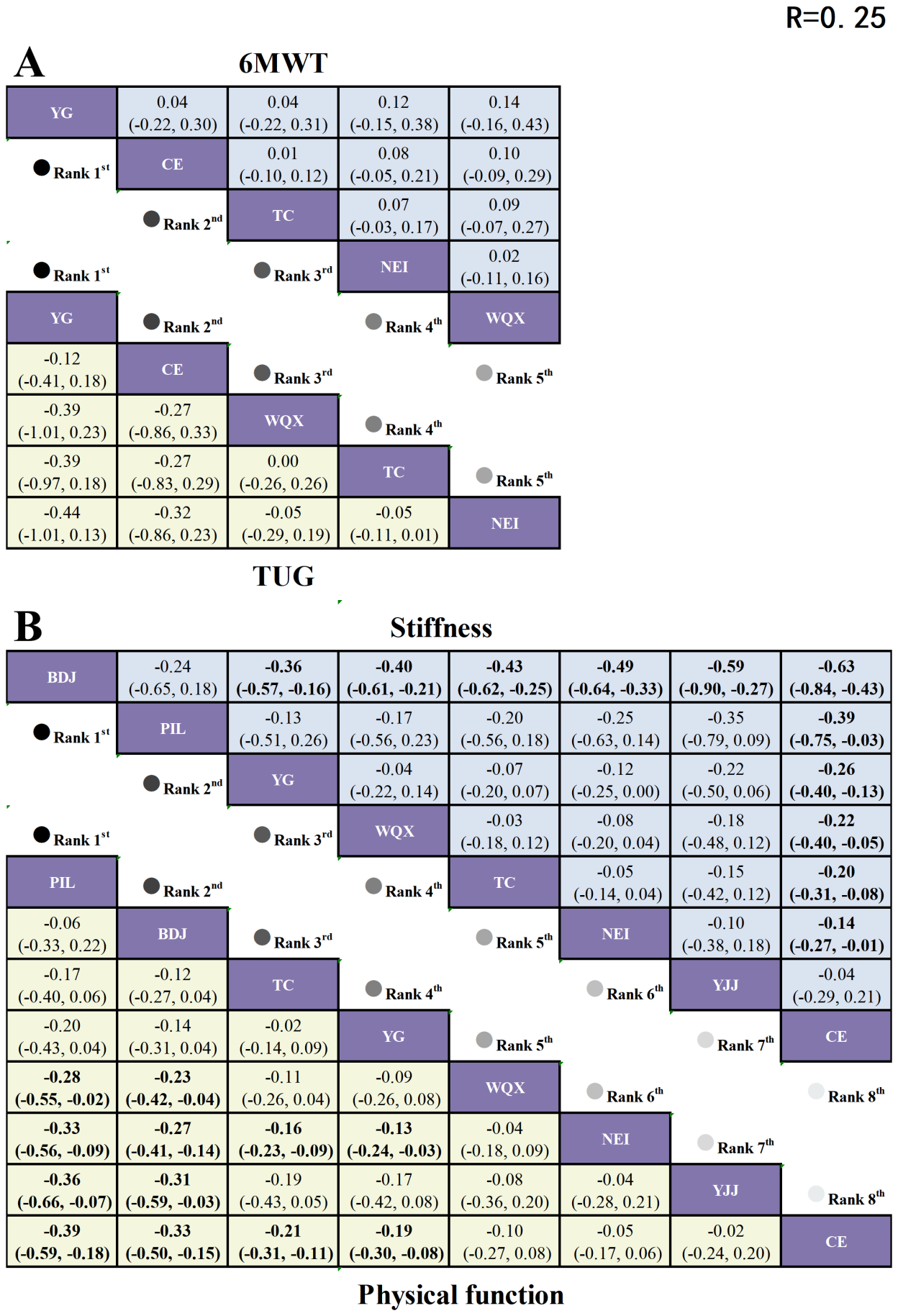


**Figure 16** League table of pairwise comparisons for all outcomes (R = 0.25)

Note: This league table presents pairwise comparisons of interventions across four outcomes (6MWT, TUG, Stiffness, and Physical function) using a correlation coefficient of 0.25 for imputing change-score standard deviations. Panel A displays results for 6MWT and TUG; Panel B displays results for Stiffness and Physical function. Values represent mean differences with 95% confidence intervals.


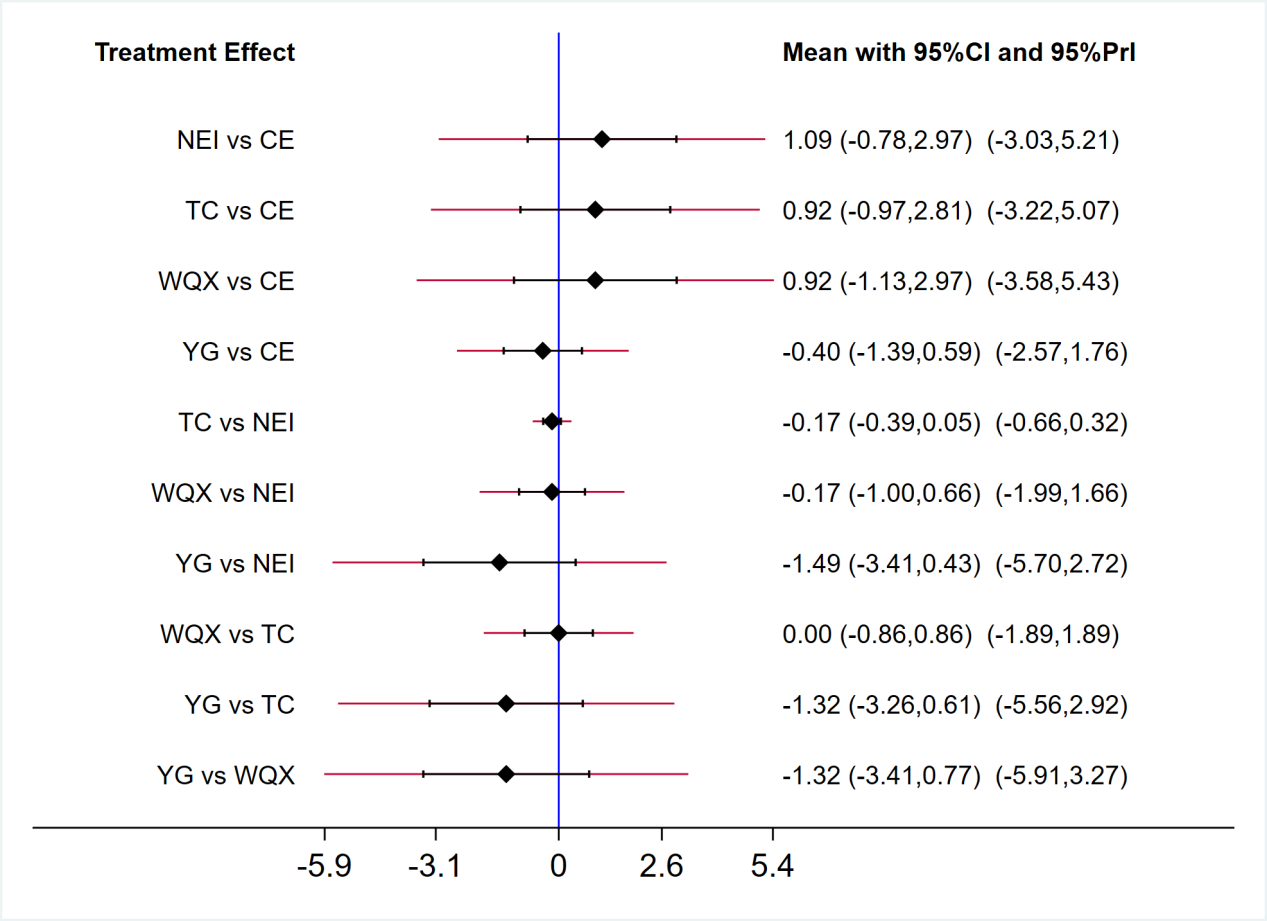


**Figure 17:** 95% prediction intervals for TUG


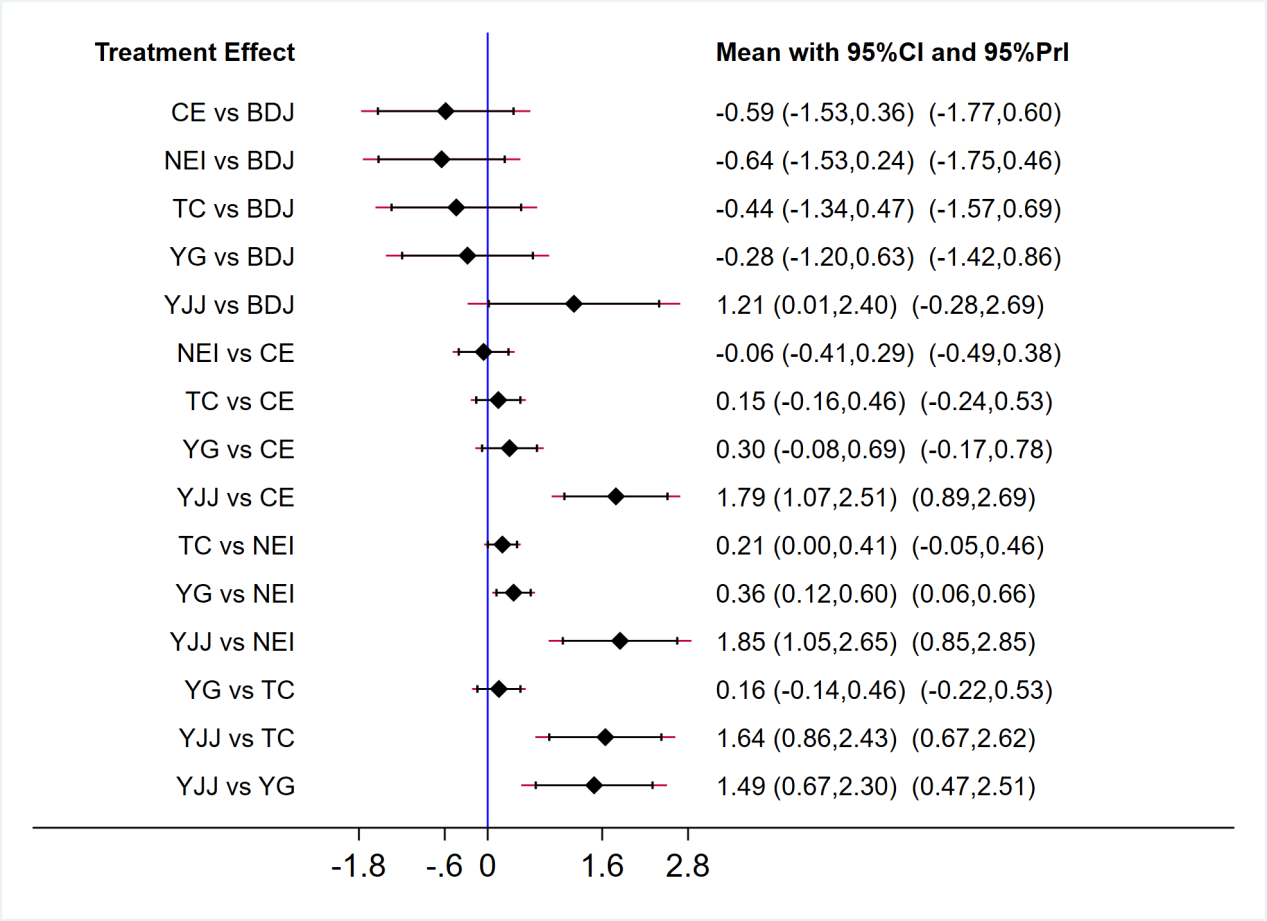


**Figure 18:** 95% prediction intervals for mental health


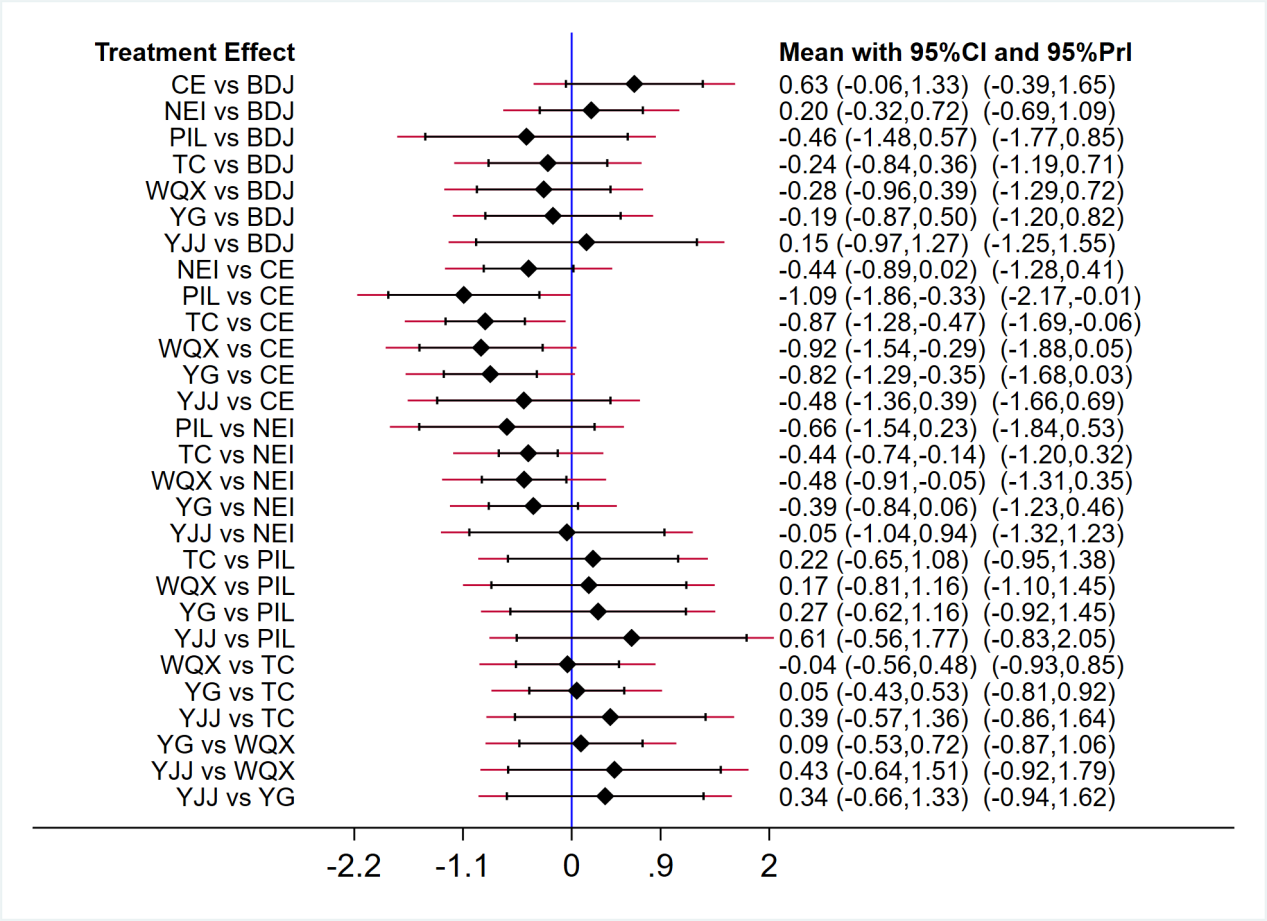


**Figure 19:** 95% prediction intervals for WOMAC pain


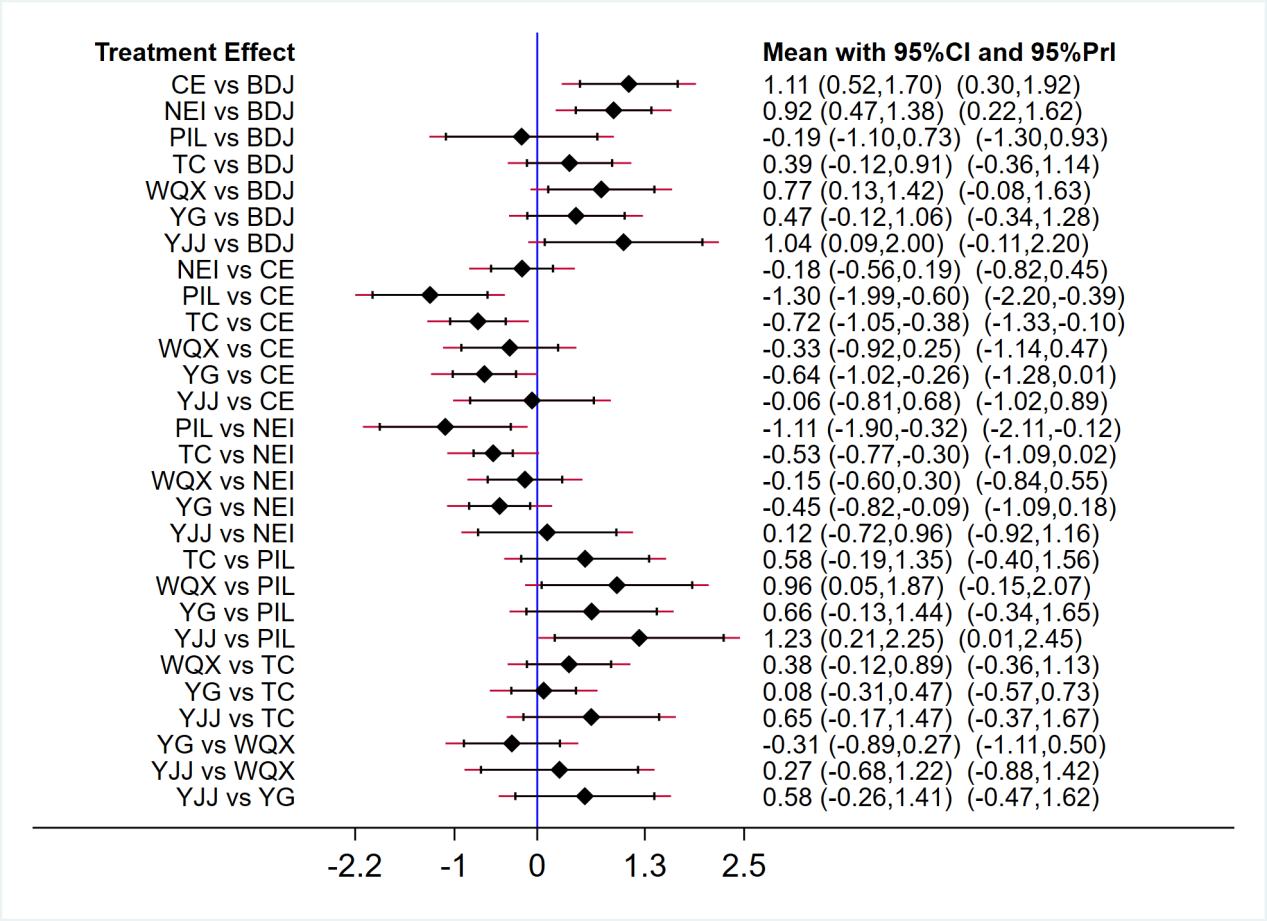


**Figure 20:** 95% prediction intervals for WOMAC physical function


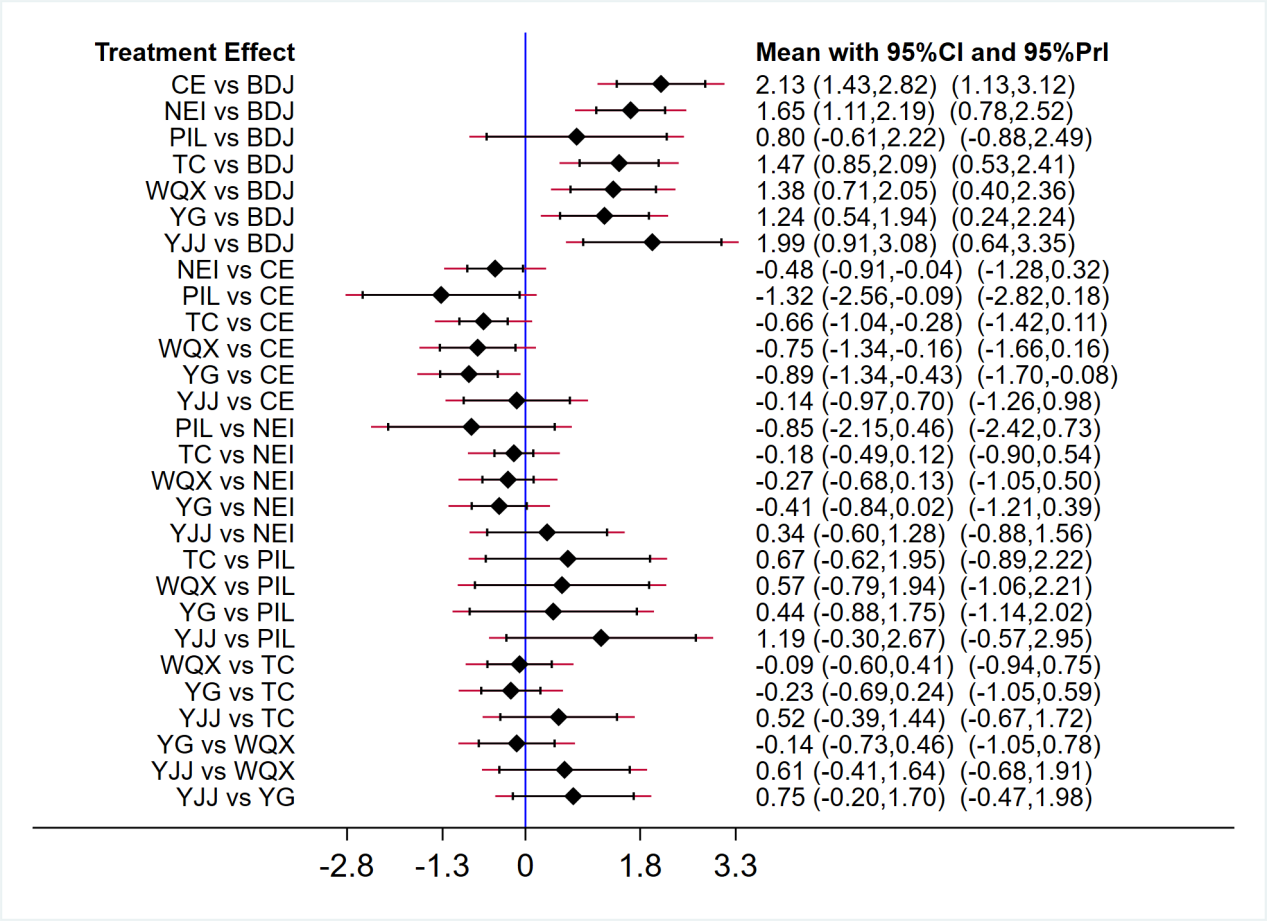


**Figure 21:** 95% prediction intervals for WOMAC stiffness


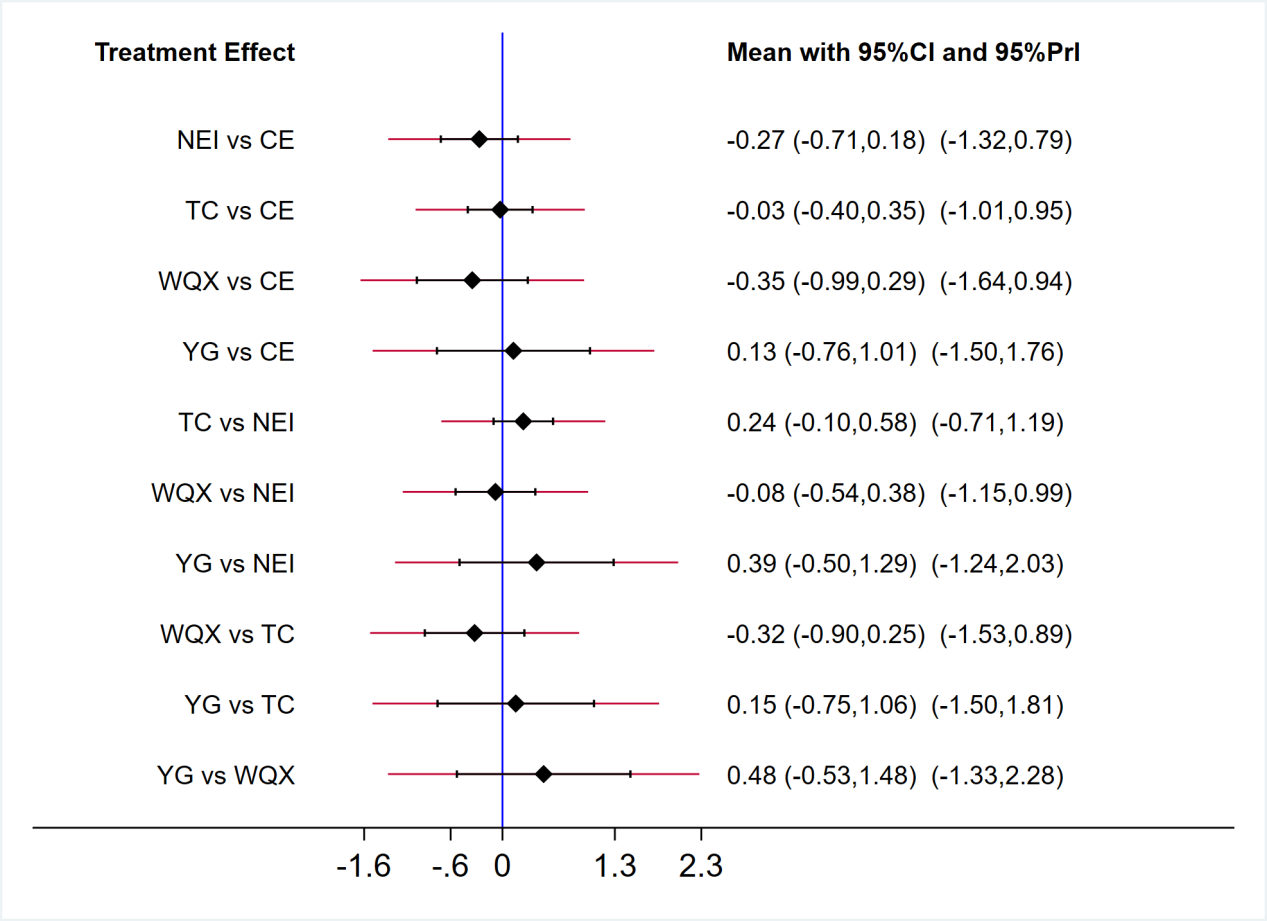


**Figure 22**: 95% prediction intervals for 6MWT
